# Supplementary material for: Hydroxamic acid-modified peptide microarrays for profiling isozyme-selective interactions and inhibition of histone deacetylases
Source: Nat Commun. 2021 Jan 4;12:62. doi: 10.1038/s41467-020-20250-9 (PMC7782793; doi:10.1038/s41467-020-20250-9)
Supplement: Supplementary file 1 — Supplementary Information [file 41467_2020_20250_MOESM1_ESM.pdf]

Supplementary information

Hydroxamic acid-modified peptide microarrays for profiling isozyme-selective interactions and inhibition of histone deacetylases

Moreno-Yruela, C. et al.

Supplementary Figures 1–13	S2
Supplementary Tables 1–4	S13
Supplementary Methods	S23
Supplementary References	S11

Supplementary Figures

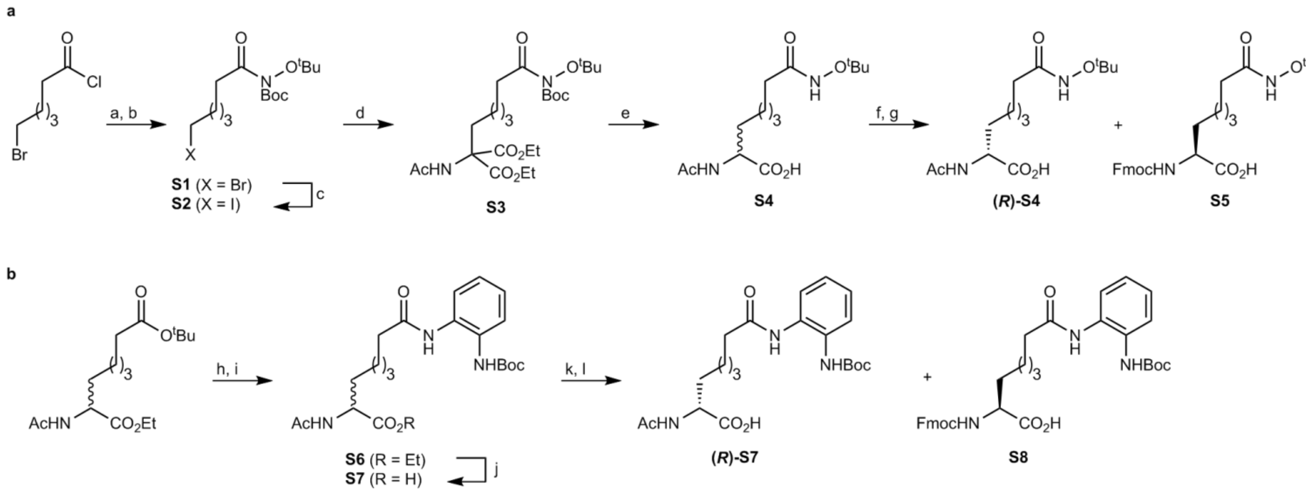

Supplementary Fig. 1. Scalable synthesis of protected (a) Asuha and (b) Asuapa amino acid building blocks. **a** Reagents and conditions: (a) H_2NO^tBu (1.1 equiv), Et_2^iPrN (2.0 equiv), anh. CH_2Cl_2, r.t., 2 h; (b) Boc_2O (1.8 equiv), Et_3N (1 equiv), DMAP (0.2 equiv), THF, r.t., overnight, 74–94%; (c) NaI (1.9 equiv), acetone, reflux, overnight; (d) diethyl acetamidomalonate (1.1 equiv), NaH (1.1 equiv), DMF, r.t., overnight, 61–84%; (e) aq. NaOH (1.8 equiv), ethanol/H_2O (1:1), r.t., 4.5 h, then aq. HCl (to pH 4), reflux, overnight; (f) acylase I from *Aspergillus melleus*, $CoCl_2{\cdot}6H_2O$, phosphate buffer 0.1 M pH 7.2, 40 ºC, overnight; (g) Fmoc-OSu (0.8 equiv), 1,4-dioxane (over previous aq. mixture), pH 10, r.t., overnight, 32–74% of the (*S*)-enantiomer. **b** Reagents and conditions: (h) TFA/CH_2Cl_2 (1:2), r.t., 2.5 h; (i) *N*-Boc-1,2-diphenylenediamine (1.5 equiv), EDC (1.5 equiv), HOBt (1.5 equiv), Et_2^iPrN (3.0 equiv), CH_2Cl_2, r.t., overnight, 62%; (j) aq. LiOH (1.6 equiv), ethanol/H_2O (1:1), r.t., 3 h, 88%; (k) acylase I from *Aspergillus melleus*, $CoCl_2{\cdot}6H_2O$, phosphate buffer 0.1 M pH 7.2, 40 ºC, 2 days; (l) Fmoc-OSu (0.8 equiv), 1,4-dioxane (over previous aq. mixture), pH 9, r.t., overnight, 49% of the (*S*)-enantiomer.

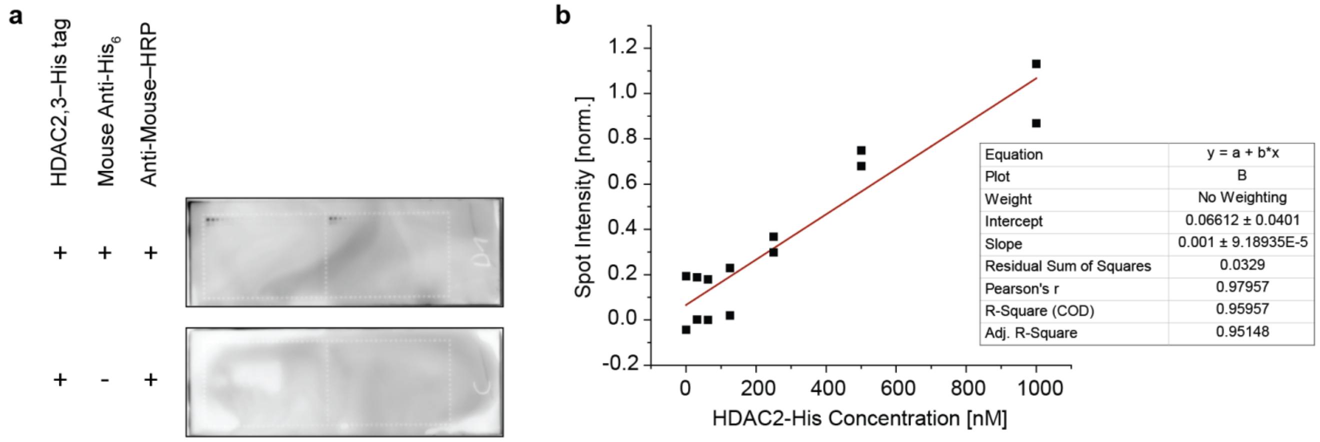

Supplementary Fig. 2. Linearity of the microarray signal. **a** Image obtained upon incubation of spotted HDAC2 (row A) and HDAC3 (row B) with an anti-His tag antibody and corresponding secondary HRP-conjugated antibody, and negative secondary antibody control. **b** Linear dependence of the obtained luminescence signal and the initial concentration of HDAC2 printed on the microarray slide (data represent n = 2 independent experiments).

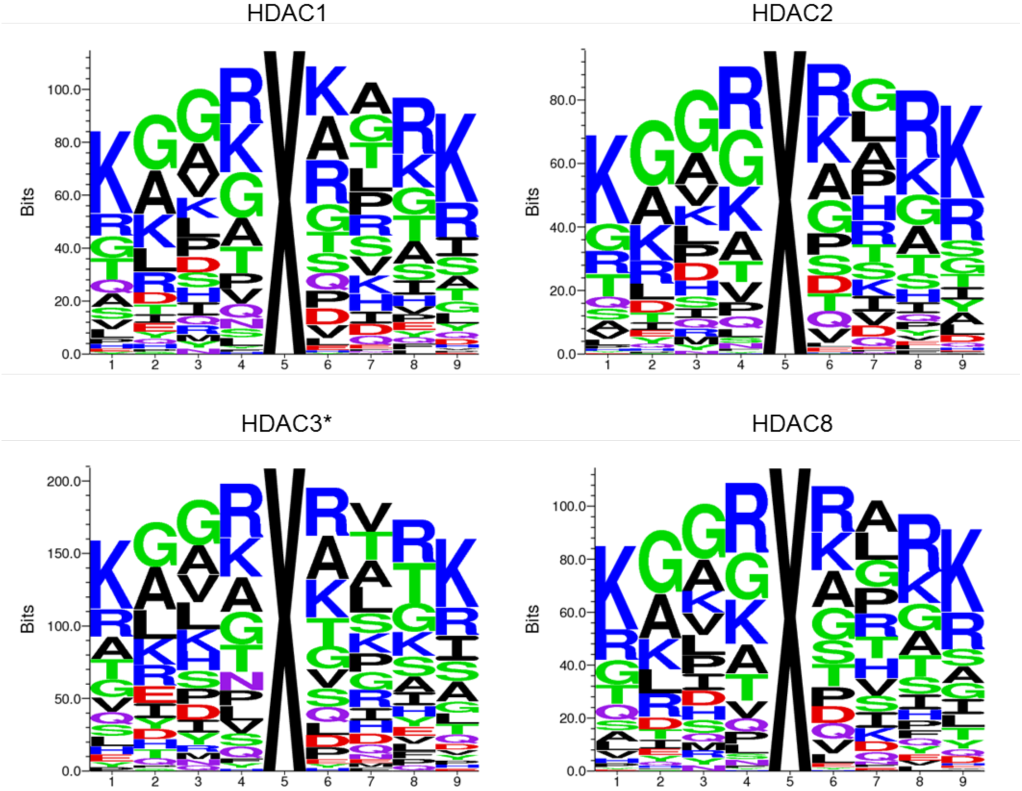

Supplementary Fig. 3. Sequence logos relative to Fig. 3 (from Seq2Logo server, DTU, Denmark)[1] obtained from the relative microarray signal intensity upon incubation with HDAC1 (10 nM), HDAC2 (2 nM), HDAC3/NCoR2 (10 nM) or HDAC8 (10 nM).

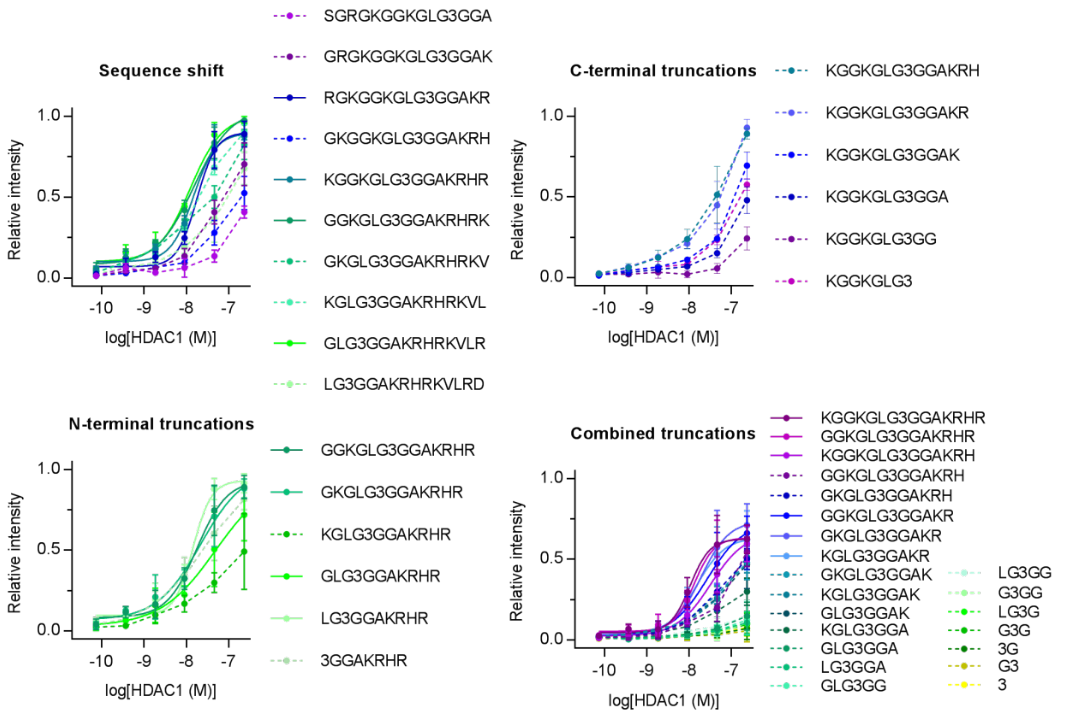

Supplementary Fig. 4. Effect of H4K12Asuha sequence frame shift and truncation on HDAC1 (mean ± SEM, *n* = 4 independent experiments). Source data are provided as a Source Data file.

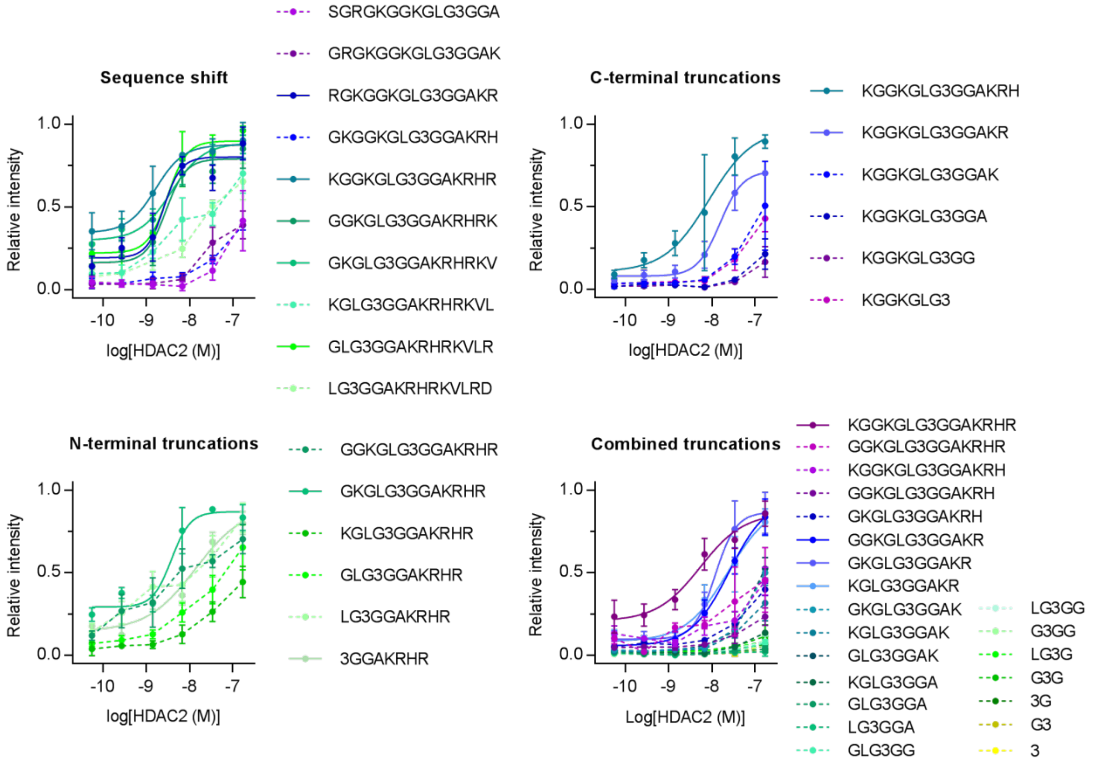

Supplementary Fig. 5. Effect of H4K12Asuha sequence frame shift and truncation on HDAC2 (mean ± SEM, *n* = 4 independent experiments). Source data are provided as a Source Data file.

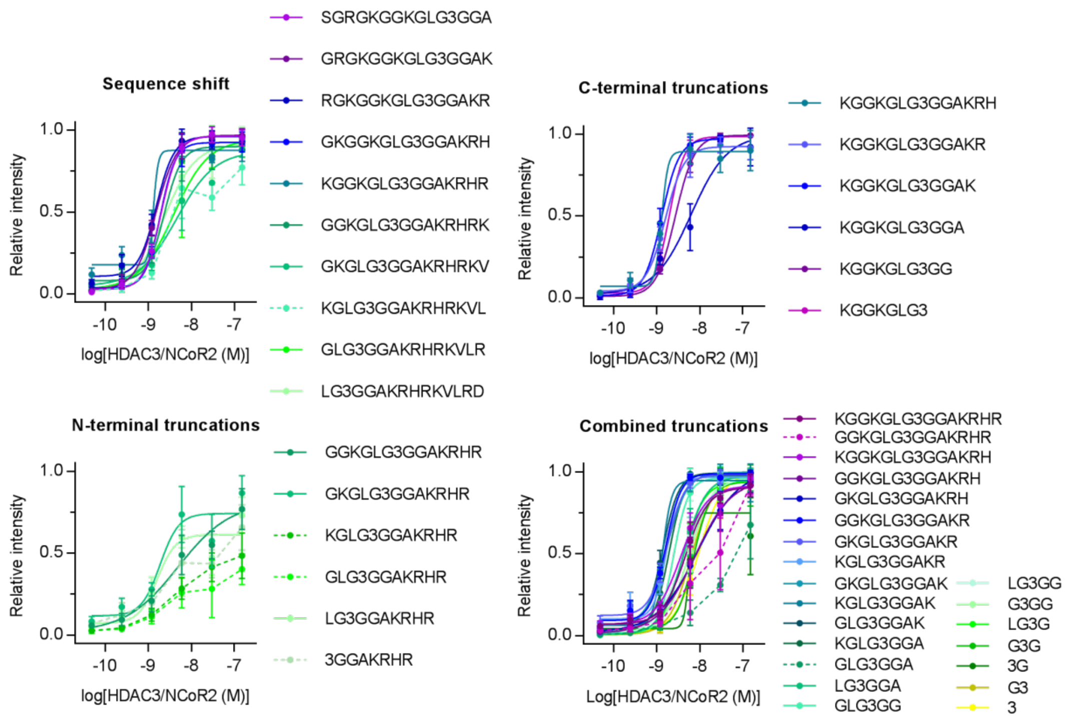

Supplementary Fig. 6. Effect of H4K12Asuha sequence frame shift and truncation on HDAC3/NCoR2 (mean ± SEM, *n* = 4 independent experiments). Source data are provided as a Source Data file.

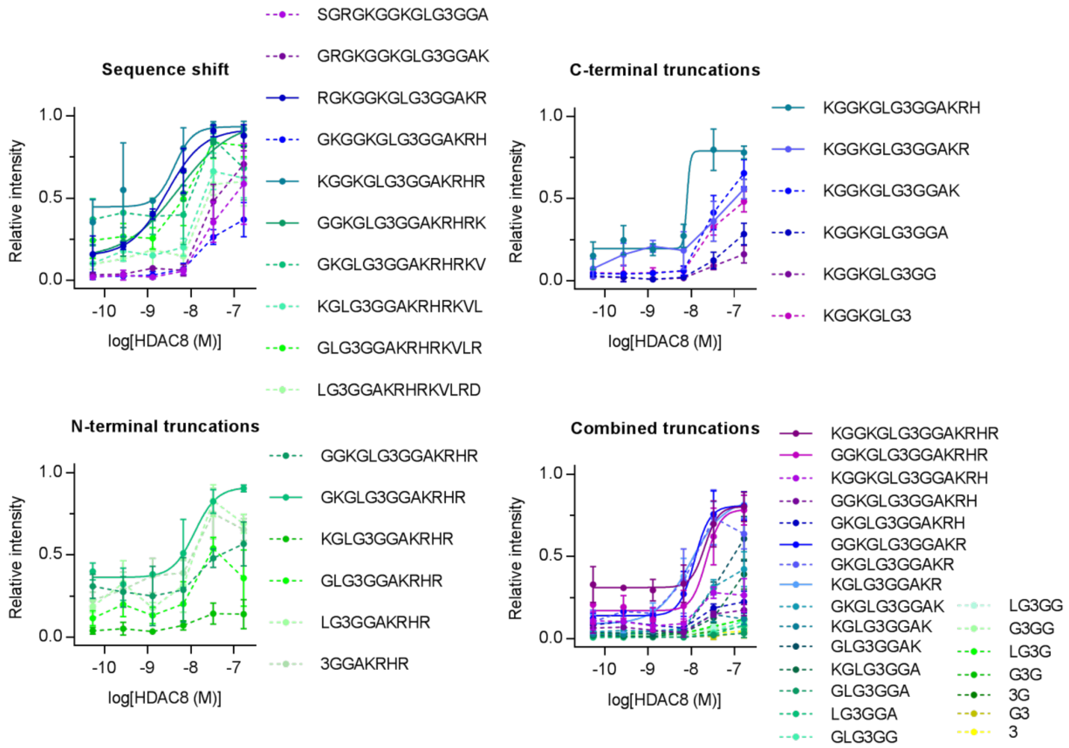

Supplementary Fig. 7. Effect of H4K12Asuha sequence frame shift and truncation on HDAC8 (mean ± SEM, *n* = 4 independent experiments). Source data are provided as a Source Data file.

Peptide	Sequence
**1a**: H4(5–19)K12Asuha	Ac-KGGKGLGXGGAKRHR-W-NH_2
**2a**: H4(1–15)K12Asuha	Ac-SGRGKGGKGLGXGGA-W-NH_2
**3a**: H4(5–14)K12Asuha	Ac-KGGKGLGXGG-W-NH_2
**4a**: H4(9–23)K12Asuha	Ac-GLGXGGAKRHRKVLR-W-NH_2
**5a**: H4(10–19)K12Asuha	Ac-LGXGGAKRHR-W-NH_2

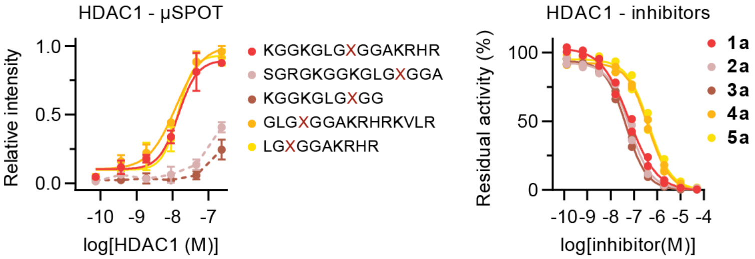

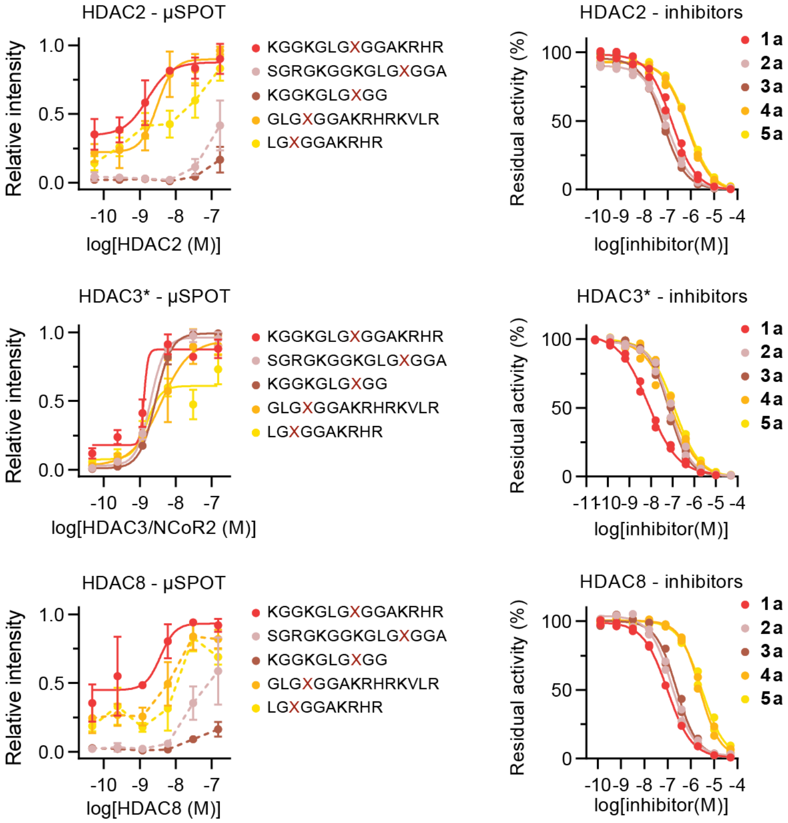

	K_i (nM)			
	HDAC1	HDAC2	HDAC3*	HDAC8
**1a**	14.1 ± 0.1	19.9 ± 0.1	1.76 ± 0.01	64.0 ± 0.1
**2a**	14.7 ± 0.1	13.2 ± 0.1	17.9 ± 0.1	100 ± 1
**3a**	10.5 ± 0.1	8.34 ± 0.04	15.6 ± 0.1	157 ± 1
**4a**	95.8 ± 0.6	90.8 ± 0.7	20.3 ± 0.3	1560 ± 10
**5a**	101 ± 1	101 ± 1	27.5 ± 0.1	1910 ± 20

Supplementary Fig. 8. Comparison of the microarray binding affinity and the inhibitory potency of resynthesized peptides **1a**–**5a** against HDACs 1–3 and 8, and calculated inhibitor constants (microarray data represent mean ± SEM, *n* = 4 independent experiments; inhibition data represent *n* = 2 independent experiments). Source data are provided as a Source Data file.

a

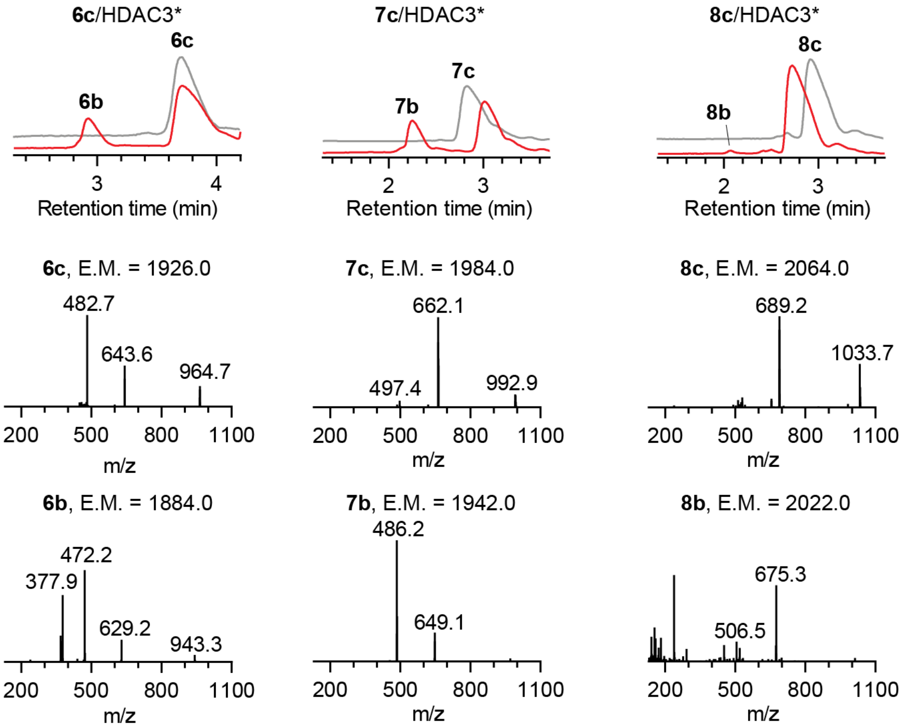

	Conversion (%)			
	HDAC1	HDAC2	HDAC3*	HDAC8
**6c**	50.2 ± 0.1	60.7 ± 0.5	22 ± 3	7.5 ± 0.3
**7c**	47 ± 1	53.7 ± 0.6	32.8 ± 0.4	5.3 ± 0.3
**8c**	1.4 ± 0.1	1.5 ± 0.2	1.5 ± 0.1	1.4 ± 0.1

b

	**K_i (nM)**			
	HDAC1	HDAC2	HDAC3*	HDAC8
**6a**	16.6 ± 0.2	142 ± 4	4.4 ± 0.1	68.8 ± 0.7
**7a**	240 ± 1	287 ± 3	235 ± 1	3830 ± 39
**8a**	1830 ± 30	1460 ± 20	1830 ± 80	8900 ± 500

Supplementary Fig. 9. a Sample traces of LC-MS deacetylation assays performed with Kac-containing (**c**) peptides (gray trace: control, red trace: reaction), and relative conversion by class I HDACs (data represent mean ± SD, n ≥ 2 independent experiments). Differences in retention time relative to the control were derived from instrumental variability. E.M.: exact mass. *HDAC3 tested in combination with the DAD domain of NCoR2. **b** Inhibition of class I HDACs by hydroxamic acid-modified peptides **6a–8a** (data represent mean ± SD, n ≥ 2 independent experiments).

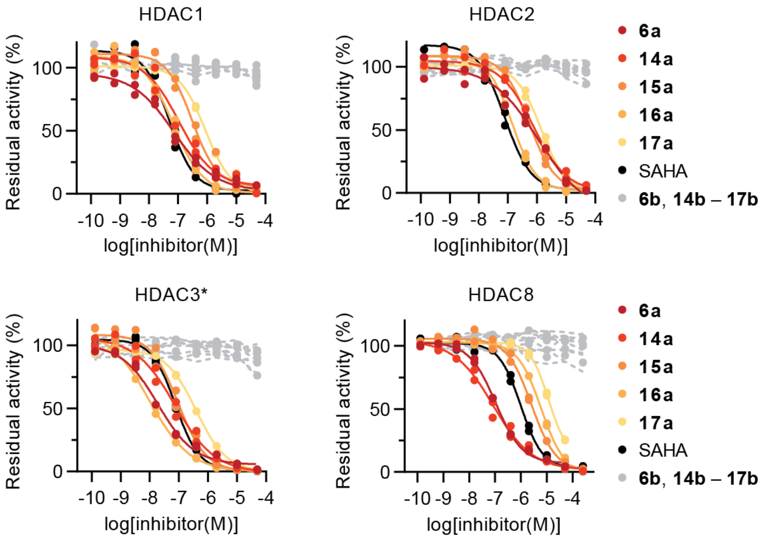

Supplementary Fig. 10. Dose-response HDAC inhibition curves for Asuha (X)-containing peptides (**6a**, **14a–17a**), the HDAC inhibitor SAHA and lysine-containing controls (**6b**, **14b–17b**). Data represent n = 2 independent experiments. Source data are provided as a Source Data file.

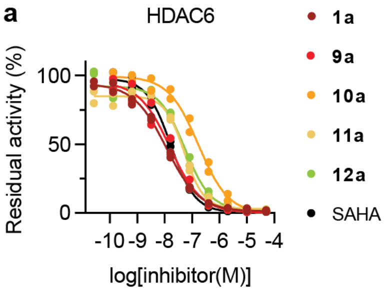

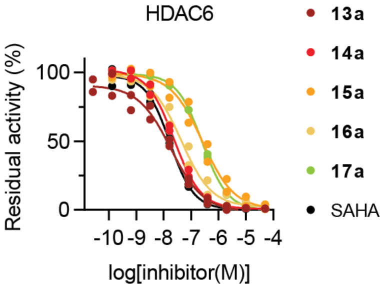

	K_i (nM)
	HDAC6
**1a**	5.31 ± 0.03
**9a**	6.91 ± 0.08
**10a**	76.0 ± 0.6
**11a**	22.7 ± 0.1
**12a**	27.6 ± 0.1
**13a**	8.3 ± 0.1
**14a**	10.5 ± 0.04
**15a**	114 ± 2
**16a**	19.3 ± 0.2
**17a**	113 ± 1
SAHA	7.45 ± 0.04

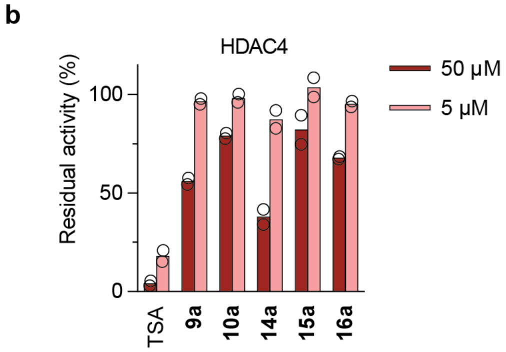

Supplementary Fig. 11. a Dose-response inhibition curves and inhibitor constants for Asuha-containing peptides **1a** and **9a–17a**, and for control inhibitor SAHA against HDAC6 (data represent n = 2 independent experiments). **b** Inhibition of the class IIa member HDAC4, data represent n = 2 internal replicates. TSA: trichostatin A, positive control. Source data are provided as a Source Data file.

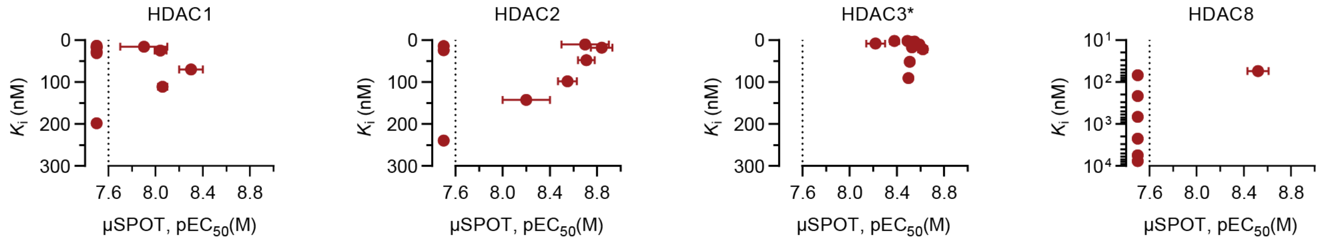

Supplementary Fig. 12. Scatter plot representation of microarray screening data and the corresponding inhibitor constants in solution, associated to Fig. 6d. Data points on the left of the plot are weak microarray binders. Microarray data represent mean ± SEM, *n* = 4 independent experiments; inhibition data represent mean of *n* = 2 independent experiments.

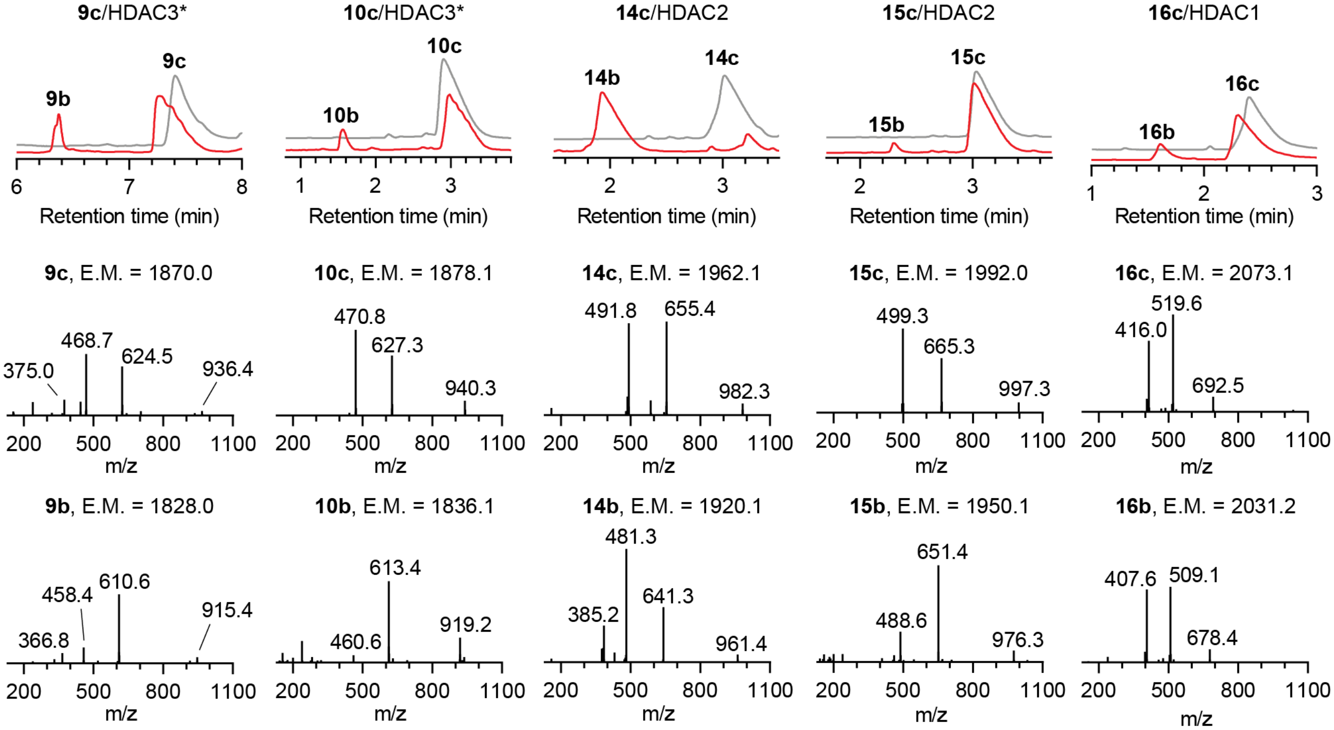

	Conversion (%)			
	HDAC1	HDAC2	HDAC3*	HDAC8
**9c**	16.7 ± 0.7	30 ± 2	16 ± 2	< 0.1
**10c**	7 ± 1	12.2 ± 0.5	11.7 ± 0.6	0.7 ± 0.1
**14c**	66 ± 4	85 ± 1	16.3 ± 0.4	6.0 ± 0.3
**15c**	2.5 ± 0.3	4.4 ± 0.3	3.3 ± 0.4	< 0.1
**16c**	19 ± 4	26.7 ± 0.9	2.9 ± 0.5	< 0.1

Supplementary Fig. 13. Sample traces of LCMS deacetylation assays performed with Kac-containing (**c**) peptides (gray trace: control, red trace: reaction) and relative conversion by class I HDACs (data represent mean ± SD, *n* ≥ 2 independent experiments). Differences in retention time relative to the control were derived from instrumental variability. E.M.: exact mass. *HDAC3 tested in combination with the DAD domain of NCoR2.

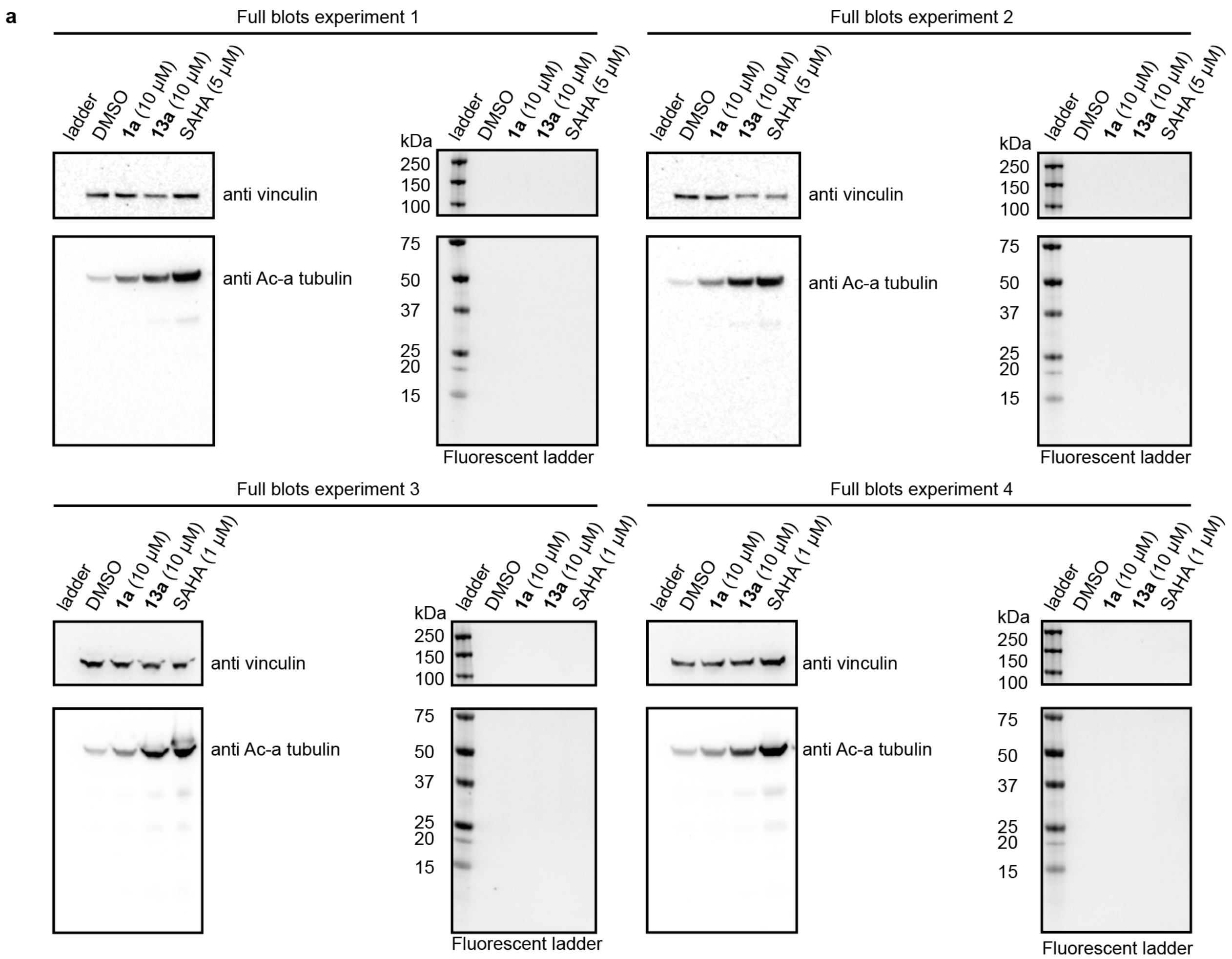

Supplementary Fig. 14. Full Western blots for the upregulation of (**a**) tubulin acetylation, (**b**) H3K27 acetylation and (**c**) H3K36 acetylation upon treatment with DMSO (negative control), compounds **1a** and **13a**, and SAHA (positive control). Vinculin loading controls and fluorescent ladders are shown for each experiment. Source data are provided as a Source Data file.

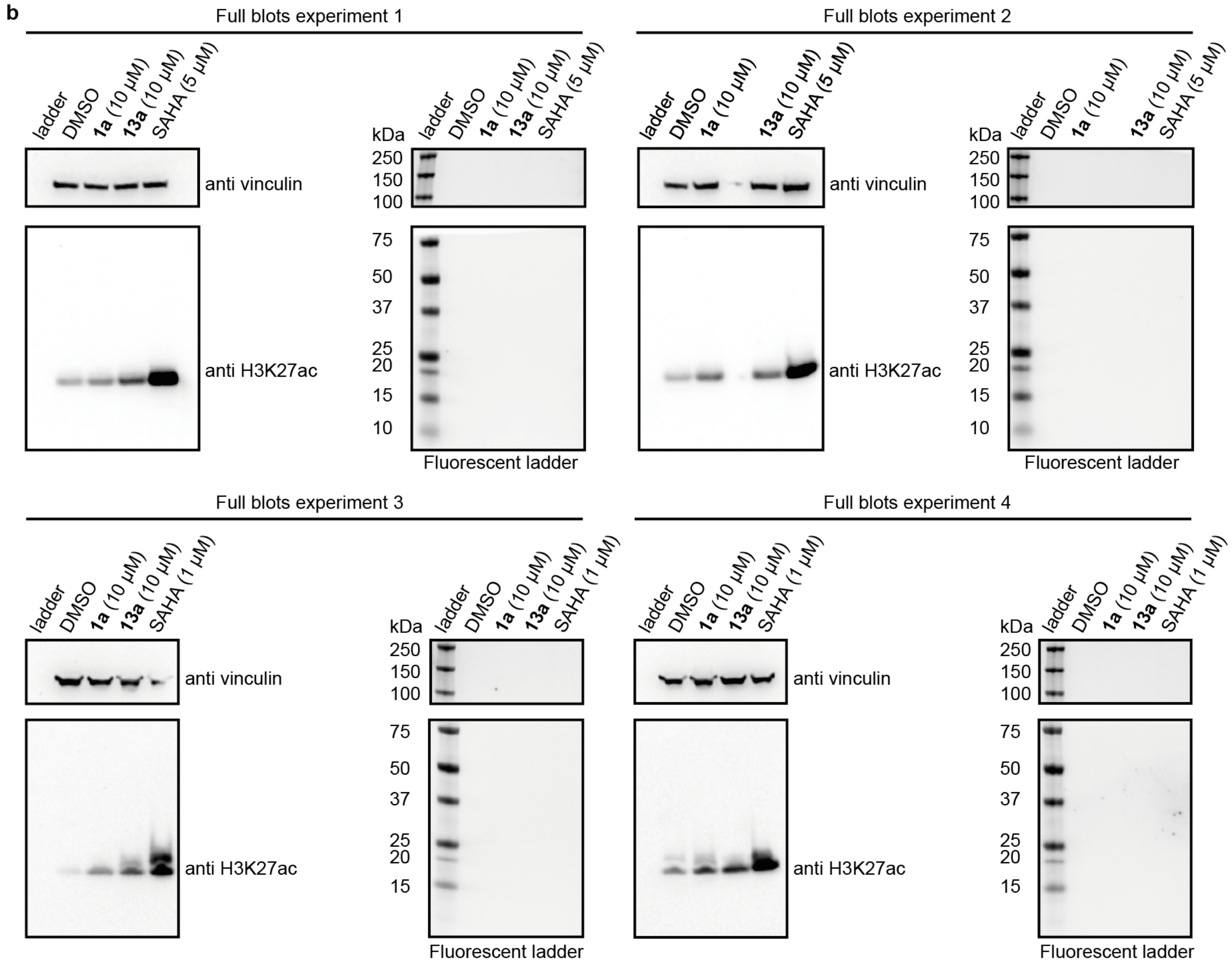

Supplementary Fig. 14 (continued)**.**

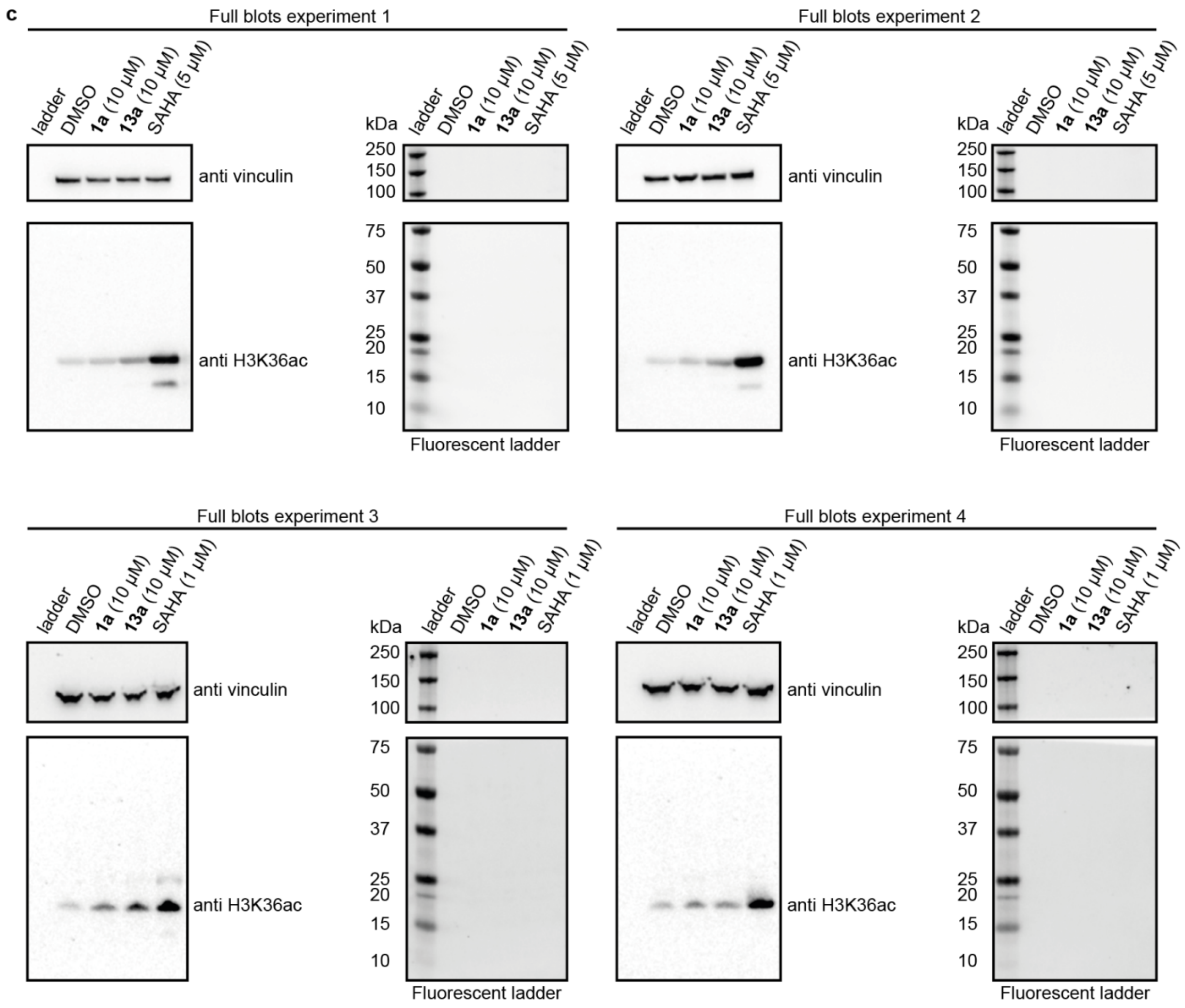

Supplementary Fig. 14 (continued)**.**

Supplementary Tables

Supplementary Table 1. Crude purity of μSPOT peptides synthesized with C-terminal Rink amide linker. Concomitant cleavage and deprotection afforded soluble peptides which were analyzed by MALDI, HPLC and LCMS (data represent mean ± SD, n ≥ 1). HPLC signal was often weak (*) and peaks could not be integrated with accuracy. Protecting group byproducts were not separated prior to analysis, and major impurities were identified by LCMS to be truncated sequences, as well as Met oxidation products when relevant (†). X: Asuha(*t*Bu). N.D.: not determined.

Peptide	Sequence	M.W.	*n*	Purity (%)
H2A(13–28)K13X	Ac-XAKTRSSRAGLQFPVG-NH_2	1857	3	76 ± 1
H2A(76–91)	Ac-TRIIPRHLQLAIRNDE-NH_2	1985	3	45 ± 3
H2A(85–100)K99X	Ac-LAIRNDEELNKLLGXV-NH_2	1979	3	42 ± 1
H2A(109–124)K119X	Ac-PNIQAVLLPKXTESHH-NH_2	1966	3	64 ± 1
H2B(7–22)K11X	Ac-APAPXKGSKKAVTKAQ-NH_2	1764	1	34
H2B(7–22)K12X	Ac-APAPKXGSKKAVTKAQ-NH_2	1764	1	25
H2B(10–25)K12X	Ac-PKXGSKKAVTKAQKKD-NH_2	1896	1	>40*
H2B(19–34)K30X	Ac-TKAQKKDGKKRXRSRK-NH_2	2097	1	>37*
H2B(88–103)	Ac-TITSREIQTAVRLLLP-NH_2	1851	1	N.D.*
H3(7–22)K9X	Ac-ARXSTGGKAPRKQLAT-NH_2	1824	1	34
H3(13–28)K23X	Ac-GKAPRKQLATXAARKS-NH_2	1865	1	>30*
H3(31–46)K36X	Ac-ATGGVXKPHRYRPGTV-NH_2	1878	1	46
H3(55–70)K56X	Ac-QXSTELLIRKLPFQRL-NH_2	2124	1	79
H3(79–94)K79X	Ac-XTDLRFQSSAVMALQE-NH_2	1978	1	18†
H4(1–16)K5X	Ac-SGRGXGGKGLGKGGAK-NH_2	1569	1	>30*
H4(4–19)K16X	Ac-GKGGKGLGKGGAXRHR-NH_2	1718	1	>40*
H4(16–31)K31X	Ac-KRHRKVLRDNIQGITX-NH_2	2116	1	N.D.*
H4(49–64)K59X	Ac-LIYEETRGVLXVFLEN-NH_2	2077	1	25
H4(76–91)K79X	Ac-AKRXTVTAMDVVYALK-NH_2	1949	1	29†

Supplementary Table 2. Microarray peptide sequences based on histones H2A (type 1, P0C0S8), H2B (type 1-C/E/F/G/I, P6207), H3.1 (P68431) and H4 (P62805), with Asuha (X) in exchange of each K residue in the sequence.

Position	Sequence	Name	Position	Sequence	Name
A 1	SGRGXQGGKARAKAKT	H2A 1-16 (K5X)	I 1	-	
A 2	SGRGKQGGXARAKAKT	H2A 1-16 (K9X)	I 2	-	
A 3	SGRGKQGGKARAXAKT	H2A 1-16 (K13X)	I 3	-	
A 4	SGRGKQGGKARAKAXT	H2A 1-16 (K15X)	I 4	-	
A 5	GXQGGKARAKAKTRSS	H2A 4-19 (K5X)	I 5	-	
A 6	GKQGGXARAKAKTRSS	H2A 4-19 (K9X)	I 6	-	
A 7	GKQGGKARAXAKTRSS	H2A 4-19 (K13X)	I 7	-	
A 8	GKQGGKARAKAXTRSS	H2A 4-19 (K15X)	I 8	-	
A 9	GGXARAKAKTRSSRAG	H2A 7-22 (K9X)	I 9	-	
A10	GGKARAXAKTRSSRAG	H2A 7-22 (K13X)	I10	-	
A11	GGKARAKAXTRSSRAG	H2A 7-22 (K15X)	I11	-	
A12	ARAXAKTRSSRAGLQF	H2A 10-25 (K13X)	I12	-	
A13	ARAKAXTRSSRAGLQF	H2A 10-25 (K15X)	I13	-	
A14	XAKTRSSRAGLQFPVG	H2A 13-28 (K13X)	I14	-	
A15	KAXTRSSRAGLQFPVG	H2A 13-28 (K15X)	I15	-	
A16	TRSSRAGLQFPVGRVH	H2A 16-31	I16	-	
A17	SRAGLQFPVGRVHRLL	H2A 19-34	I17	-	
A18	GLQFPVGRVHRLLRXG	H2A 22-37 (K36X)	I18	-	
A19	FPVGRVHRLLRXGNYA	H2A 25-40 (K36X)	I19	-	
A20	GRVHRLLRXGNYAERV	H2A 28-43 (K36X)	I20	-	
A21	HRLLRXGNYAERVGAG	H2A 31-46 (K36X)	I21	-	
A22	LRXGNYAERVGAGAPV	H2A 34-49 (K36X)	I22	-	
A23	GNYAERVGAGAPVYLA	H2A 37-52	I23	-	
A24	AERVGAGAPVYLAAVL	H2A 40-55	I24	-	
B 1	VGAGAPVYLAAVLEYL	H2A 43-58	J 1	SREIQTAVRLLLPGEL	H2B 91-106
B 2	GAPVYLAAVLEYLTAE	H2A 46-61	J 2	IQTAVRLLLPGELAXH	H2B 94-109 (K108X)
B 3	VYLAAVLEYLTAEILE	H2A 49-64	J 3	AVRLLLPGELAXHAVS	H2B 97-112 (K108X)
B 4	AAVLEYLTAEILELAG	H2A 52-67	J 4	LLLPGELAXHAVSEGT	H2B 100-115 (K108X)
B 5	LEYLTAEILELAGNAA	H2A 55-70	J 5	PGELAXHAVSEGTKAV	H2B 103-118 (K108X)
B 6	LTAEILELAGNAARDN	H2A 58-73	J 6	PGELAKHAVSEGTXAV	H2B 103-118 (K116X)
B 7	EILELAGNAARDNXKT	H2A 61-76 (K74X)	J 7	LAXHAVSEGTKAVTKY	H2B 106-121 (K108X)
B 8	EILELAGNAARDNKXT	H2A 61-76 (K75X)	J 8	LAKHAVSEGTXAVTKY	H2B 106-121 (K116X)
B 9	ELAGNAARDNXKTRII	H2A 64-79 (K74X)	J 9	LAKHAVSEGTKAVTXY	H2B 106-121 (K120X)
B10	ELAGNAARDNKXTRII	H2A 64-79 (K75X)	J10	HAVSEGTXAVTKYTSS	H2B 109-124 (K116X)
B11	GNAARDNXKTRIIPRH	H2A 67-82 (K74X)	J11	HAVSEGTKAVTXYTSS	H2B 109-124 (K120X)
B12	GNAARDNKXTRIIPRH	H2A 67-82 (K75X)	J12	AVSEGTXAVTKYTSSK	H2B 110-125 (K116X)
B13	ARDNXKTRIIPRHLQL	H2A 70-85 (K74X)	J13	AVSEGTKAVTXYTSSK	H2B 110-125 (K120X)
B14	ARDNKXTRIIPRHLQL	H2A 70-85 (K75X)	J14	AVSEGTKAVTKYTSSX	H2B 110-125 (K125X)
B15	NXKTRIIPRHLQLAIR	H2A 73-88 (K74X)	J15	ARTXQTARKSTGGKAP	H3 1-16 (K4X)
B16	NKXTRIIPRHLQLAIR	H2A 73-88 (K75X)	J16	ARTKQTARXSTGGKAP	H3 1-16 (K9X)
B17	TRIIPRHLQLAIRNDE	H2A 76-91	J17	ARTKQTARKSTGGXAP	H3 1-16 (K14X)
B18	IPRHLQLAIRNDEELN	H2A 79-94	J18	XQTARKSTGGKAPRKQ	H3 4-19 (K4X)
B19	HLQLAIRNDEELNXLL	H2A 82-97 (K95X)	J19	KQTARXSTGGKAPRKQ	H3 4-19 (K9X)
B20	LAIRNDEELNXLLGKV	H2A 85-100 (K95X)	J20	KQTARKSTGGXAPRKQ	H3 4-19 (K14X)
B21	LAIRNDEELNKLLGXV	H2A 85-100 (K99X)	J21	KQTARKSTGGKAPRXQ	H3 4-19 (K18X)
B22	RNDEELNXLLGKVTIA	H2A 88-103 (K95X)	J22	ARXSTGGKAPRKQLAT	H3 7-22 (K9X)
B23	RNDEELNKLLGXVTIA	H2A 88-103 (K99X)	J23	ARKSTGGXAPRKQLAT	H3 7-22 (K14X)
B24	EELNXLLGKVTIAQGG	H2A 91-106 (K95X)	J24	ARKSTGGKAPRXQLAT	H3 7-22 (K18X)
C 1	EELNKLLGXVTIAQGG	H2A 91-106 (K99X)	K 1	STGGXAPRKQLATKAA	H3 10-25 (K14X)

C 2	NXLLGKVTIAQGGVLP	H2A 94-109 (K95X)	K 2	STGGKAPRXQLATKAA	H3 10-25 (K18X)
C 3	NKLLGXVTIAQGGVLP	H2A 94-109 (K99X)	K 3	STGGKAPRKQLATXAA	H3 10-25 (K23X)
C 4	LGXVTIAQGGVLPNIQ	H2A 97-112 (K99X)	K 4	GXAPRKQLATKAARKS	H3 13-28 (K14X)
C 5	VTIAQGGVLPNIQAVL	H2A 100-115	K 5	GKAPRXQLATKAARKS	H3 13-28 (K18X)
C 6	AQGGVLPNIQAVLLPX	H2A 103-118 (K118X)	K 6	GKAPRKQLATXAARKS	H3 13-28 (K23X)
C 7	GVLPNIQAVLLPXKTE	H2A 106-121 (K118X)	K 7	GKAPRKQLATKAARXS	H3 13-28 (K27X)
C 8	GVLPNIQAVLLPKXTE	H2A 106-121 (K119X)	K 8	PRXQLATKAARKSAPA	H3 16-31 (K18X)
C 9	PNIQAVLLPXKTESHH	H2A 109-124 (K118X)	K 9	PRKQLATXAARKSAPA	H3 16-31 (K23X)
C10	PNIQAVLLPKXTESHH	H2A 109-124 (K119X)	K10	PRKQLATKAARXSAPA	H3 16-31 (K27X)
C11	QAVLLPXKTESHHKAK	H2A 112-127 (K118X)	K11	QLATXAARKSAPATGG	H3 19-34 (K23X)
C12	QAVLLPKXTESHHKAK	H2A 112-127 (K119X)	K12	QLATKAARXSAPATGG	H3 19-34 (K27X)
C13	QAVLLPKKTESHHXAK	H2A 112-127 (K125X)	K13	TXAARKSAPATGGVKK	H3 22-37 (K23X)
C14	QAVLLPKKTESHHKAX	H2A 112-127 (K127X)	K14	TKAARXSAPATGGVKK	H3 22-37 (K27X)
C15	VLLPXKTESHHKAKGK	H2A 114-129 (K118X)	K15	TKAARKSAPATGGVXK	H3 22-37 (K36X)
C16	VLLPKXTESHHKAKGK	H2A 114-129 (K119X)	K16	TKAARKSAPATGGVKX	H3 22-37 (K37X)
C17	VLLPKKTESHHXAKGK	H2A 114-129 (K125X)	K17	ARXSAPATGGVKKPHR	H3 25-40 (K27X)
C18	VLLPKKTESHHKAXGK	H2A 114-129 (K127X)	K18	ARKSAPATGGVXKPHR	H3 25-40 (K36X)
C19	VLLPKKTESHHKAKGX	H2A 114-129 (K129X)	K19	ARKSAPATGGVKXPHR	H3 25-40 (K37X)
C20	PEPAXSAPAPKKGSKK	H2B 1-16 (K5X)	K20	SAPATGGVXKPHRYRP	H3 28-43 (K36X)
C21	PEPAKSAPAPXKGSKK	H2B 1-16 (K11X)	K21	SAPATGGVKXPHRYRP	H3 28-43 (K37X)
C22	PEPAKSAPAPKXGSKK	H2B 1-16 (K12X)	K22	ATGGVXKPHRYRPGTV	H3 31-46 (K36X)
C23	PEPAKSAPAPKKGSXK	H2B 1-16 (K15X)	K23	ATGGVKXPHRYRPGTV	H3 31-46 (K37X)
C24	PEPAKSAPAPKKGSKX	H2B 1-16 (K16X)	K24	GVXKPHRYRPGTVALR	H3 34-49 (K36X)
D 1	AXSAPAPKKGSKKAVT	H2B 4-19 (K5X)	L 1	GVKXPHRYRPGTVALR	H3 34-49 (K37X)
D 2	AKSAPAPXKGSKKAVT	H2B 4-19 (K11X)	L 2	XPHRYRPGTVALREIR	H3 37-52 (K37X)
D 3	AKSAPAPKXGSKKAVT	H2B 4-19 (K12X)	L 3	RYRPGTVALREIRRYQ	H3 40-55
D 4	AKSAPAPKKGSXKAVT	H2B 4-19 (K15X)	L 4	PGTVALREIRRYQXST	H3 43-58 (K56X)
D 5	AKSAPAPKKGSKXAVT	H2B 4-19 (K16X)	L 5	VALREIRRYQXSTELL	H3 46-61 (K56X)
D 6	APAPXKGSKKAVTKAQ	H2B 7-22 (K11X)	L 6	REIRRYQXSTELLIRK	H3 49-64 (K56X)
D 7	APAPKXGSKKAVTKAQ	H2B 7-22 (K12X)	L 7	REIRRYQKSTELLIRX	H3 49-64 (K64X)
D 8	APAPKKGSXKAVTKAQ	H2B 7-22 (K15X)	L 8	RRYQXSTELLIRKLPF	H3 52-67 (K56X)
D 9	APAPKKGSKXAVTKAQ	H2B 7-22 (K16X)	L 9	RRYQKSTELLIRXLPF	H3 52-67 (K64X)
D10	APAPKKGSKKAVTXAQ	H2B 7-22 (K20X)	L10	QXSTELLIRKLPFQRL	H3 55-70 (K56X)
D11	PXKGSKKAVTKAQKKD	H2B 10-25 (K11X)	L11	QKSTELLIRXLPFQRL	H3 55-70 (K64X)
D12	PKXGSKKAVTKAQKKD	H2B 10-25 (K12X)	L12	TELLIRXLPFQRLVRE	H3 58-73 (K64X)
D13	PKKGSXKAVTKAQKKD	H2B 10-25 (K15X)	L13	LIRXLPFQRLVREIAQ	H3 61-76 (K64X)
D14	PKKGSKXAVTKAQKKD	H2B 10-25 (K16X)	L14	XLPFQRLVREIAQDFK	H3 64-79 (K64X)
D15	PKKGSKKAVTXAQKKD	H2B 10-25 (K20X)	L15	KLPFQRLVREIAQDFX	H3 64-79 (K79X)
D16	PKKGSKKAVTKAQXKD	H2B 10-25 (K23X)	L16	FQRLVREIAQDFXTDL	H3 67-82 (K79X)
D17	PKKGSKKAVTKAQKXD	H2B 10-25 (K24X)	L17	LVREIAQDFXTDLRFQ	H3 70-85 (K79X)
D18	GSXKAVTKAQKKDGKK	H2B 13-28 (K15X)	L18	EIAQDFXTDLRFQSSA	H3 73-88 (K79X)
D19	GSKXAVTKAQKKDGKK	H2B 13-28 (K16X)	L19	QDFXTDLRFQSSAVMA	H3 76-91 (K79X)
D20	GSKKAVTXAQKKDGKK	H2B 13-28 (K20X)	L20	XTDLRFQSSAVMALQE	H3 79-94 (K79X)
D21	GSKKAVTKAQXKDGKK	H2B 13-28 (K23X)	L21	LRFQSSAVMALQEACE	H3 82-97
D22	GSKKAVTKAQKXDGKK	H2B 13-28 (K24X)	L22	QSSAVMALQEACEAYL	H3 85-100
D23	GSKKAVTKAQKKDGXK	H2B 13-28 (K27X)	L23	AVMALQEACEAYLVGL	H3 88-103
D24	GSKKAVTKAQKKDGKX	H2B 13-28 (K28X)	L24	ALQEACEAYLVGLFED	H3 91-106
E 1	XAVTKAQKKDGKKRKR	H2B 16-31 (K16X)	M 1	EACEAYLVGLFEDTNL	H3 94-109
E 2	KAVTXAQKKDGKKRKR	H2B 16-31 (K20X)	M 2	EAYLVGLFEDTNLCAI	H3 97-112
E 3	KAVTKAQXKDGKKRKR	H2B 16-31 (K23X)	M 3	LVGLFEDTNLCAIHAX	H3 100-115 (K115X)
E 4	KAVTKAQKXDGKKRKR	H2B 16-31 (K24X)	M 4	LFEDTNLCAIHAXRVT	H3 103-118 (K115X)
E 5	KAVTKAQKKDGXKRKR	H2B 16-31 (K27X)	M 5	DTNLCAIHAXRVTIMP	H3 106-121 (K115X)
E 6	KAVTKAQKKDGKXRKR	H2B 16-31 (K28X)	M 6	LCAIHAXRVTIMPKDI	H3 109-124 (K115X)
E 7	KAVTKAQKKDGKKRXR	H2B 16-31 (K30X)	M 7	LCAIHAKRVTIMPXDI	H3 109-124 (K122X)

E 8	TXAQKKDGKKRKRSRK	H2B 19-34 (K20X)
E 9	TKAQXKDGKKRKRSRK	H2B 19-34 (K23X)
E10	TKAQKXDGKKRKRSRK	H2B 19-34 (K24X)
E11	TKAQKKDGXKRKRSRK	H2B 19-34 (K27X)
E12	TKAQKKDGKXRKRSRK	H2B 19-34 (K28X)
E13	TKAQKKDGKKRXRSRK	H2B 19-34 (K30X)
E14	TKAQKKDGKKRKRSRX	H2B 19-34 (K34X)
E15	QXKDGKKRKRSRKESY	H2B 22-37 (K23X)
E16	QKXDGKKRKRSRKESY	H2B 22-37 (K24X)
E17	QKKDGXKRKRSRKESY	H2B 22-37 (K27X)
E18	QKKDGKXRKRSRKESY	H2B 22-37 (K28X)
E19	QKKDGKKRXRSRKESY	H2B 22-37 (K30X)
E20	QKKDGKKRKRSRXESY	H2B 22-37 (K34X)
E21	DGXKRKRSRKESYSVY	H2B 25-40 (K27X)
E22	DGKXRKRSRKESYSVY	H2B 25-40 (K28X)
E23	DGKKRXRSRKESYSVY	H2B 25-40 (K30X)
E24	DGKKRKRSRXESYSVY	H2B 25-40 (K34X)
F 1	XRKRSRKESYSVYVYK	H2B 28-43 (K28X)
F 2	KRXRSRKESYSVYVYK	H2B 28-43 (K30X)
F 3	KRKRSRXESYSVYVYK	H2B 28-43 (K34X)
F 4	KRKRSRKESYSVYVYX	H2B 28-43 (K43X)
F 5	RSRXESYSVYVYKVLK	H2B 31-46 (K34X)
F 6	RSRKESYSVYVYXVLK	H2B 31-46 (K43X)
F 7	RSRKESYSVYVYKVLX	H2B 31-46 (K46X)
F 8	XESYSVYVYKVLKQVH	H2B 34-49 (K34X)
F 9	KESYSVYVYXVLKQVH	H2B 34-49 (K43X)
F10	KESYSVYVYKVLXQVH	H2B 34-49 (K46X)
F11	YSVYVYXVLKQVHPDT	H2B 37-52 (K43X)
F12	YSVYVYKVLXQVHPDT	H2B 37-52 (K46X)
F13	YVYXVLKQVHPDTGIS	H2B 40-55 (K43X)
F14	YVYKVLXQVHPDTGIS	H2B 40-55 (K46X)
F15	XVLKQVHPDTGISSKA	H2B 43-58 (K43X)
F16	KVLXQVHPDTGISSKA	H2B 43-58 (K46X)
F17	KVLKQVHPDTGISSXA	H2B 43-58 (K57X)
F18	XQVHPDTGISSKAMGI	H2B 46-61 (K46X)
F19	KQVHPDTGISSXAMGI	H2B 46-61 (K57X)
F20	HPDTGISSXAMGIMNS	H2B 49-64 (K57X)
F21	TGISSXAMGIMNSFVN	H2B 52-67 (K57X)
F22	SSXAMGIMNSFVNDIF	H2B 55-70 (K57X)
F23	AMGIMNSFVNDIFERI	H2B 58-73
F24	IMNSFVNDIFERIAGE	H2B 61-76
G 1	SFVNDIFERIAGEASR	H2B 64-79
G 2	NDIFERIAGEASRLAH	H2B 67-82
G 3	FERIAGEASRLAHYNX	H2B 70-85 (K85X)
G 4	IAGEASRLAHYNXRST	H2B 73-88 (K85X)
G 5	EASRLAHYNXRSTITS	H2B 76-91 (K85X)
G 6	RLAHYNXRSTITSREI	H2B 79-94 (K85X)
G 7	HYNXRSTITSREIQTA	H2B 82-97 (K85X)
G 8	XRSTITSREIQTAVRL	H2B 85-100 (K85X)
G 9	TITSREIQTAVRLLLP	H2B 88-103
G10	-	
G11	-	
G12	-	
G13	-	

M 8	IHAXRVTIMPKDIQLA	H3 112-127 (K119X)
M 9	IHAKRVTIMPXDIQLA	H3 112-127 (K122X)
M10	XRVTIMPKDIQLARRI	H3 115-130 (K115X)
M11	KRVTIMPXDIQLARRI	H3 115-130 (K122X)
M12	TIMPXDIQLARRIRGE	H3 118-133 (K122X)
M13	MPXDIQLARRIRGERA	H3 120-135 (K122X)
M14	SGRGXGGKGLGKGGAK	H4 1-16 (K5X)
M15	SGRGKGGXGLGKGGAK	H4 1-16 (K8X)
M16	SGRGKGGKGLGXGGAK	H4 1-16 (K12X)
M17	SGRGKGGKGLGKGGAX	H4 1-16 (K16X)
M18	GXGGKGLGKGGAKRHR	H4 4-19 (K5X)
M19	GKGGXGLGKGGAKRHR	H4 4-19 (K8X)
M20	GKGGKGLGXGGAKRHR	H4 4-19 (K12X)
M21	GKGGKGLGKGGAXRHR	H4 4-19 (K16X)
M22	GXGLGKGGAKRHRKVL	H4 7-22 (K8X)
M23	GKGLGXGGAKRHRKVL	H4 7-22 (K12X)
M24	GKGLGKGGAXRHRKVL	H4 7-22 (K16X)
N 1	GKGLGKGGAKRHRXVL	H4 7-22 (K20X)
N 2	LGXGGAKRHRKVLRDN	H4 10-25 (K12X)
N 3	LGKGGAXRHRKVLRDN	H4 10-25 (K16X)
N 4	LGKGGAKRHRXVLRDN	H4 10-25 (K20X)
N 5	GGAXRHRKVLRDNIQG	H4 13-28 (K16X)
N 6	GGAKRHRXVLRDNIQG	H4 13-28 (K20X)
N 7	XRHRKVLRDNIQGITK	H4 16-31 (K16X)
N 8	KRHRXVLRDNIQGITK	H4 16-31 (K20X)
N 9	KRHRKVLRDNIQGITX	H4 16-31 (K31X)
N10	RXVLRDNIQGITKPAI	H4 19-34 (K20X)
N11	RKVLRDNIQGITXPAI	H4 19-34 (K31X)
N12	LRDNIQGITXPAIRRL	H4 22-37 (K31X)
N13	NIQGITXPAIRRLARR	H4 25-40 (K31X)
N14	GITXPAIRRLARRGGV	H4 28-43 (K31X)
N15	XPAIRRLARRGGVKRI	H4 31-46 (K31X)
N16	KPAIRRLARRGGVXRI	H4 31-46 (K44X)
N17	IRRLARRGGVXRISGL	H4 34-49 (K44X)
N18	LARRGGVXRISGLIYE	H4 37-52 (K44X)
N19	RGGVXRISGLIYEETR	H4 40-55 (K44X)
N20	VXRISGLIYEETRGVL	H4 43-58 (K44X)
N21	ISGLIYEETRGVLXVF	H4 47-61 (K59X)
N22	LIYEETRGVLXVFLEN	H4 49-64 (K59X)
N23	EETRGVLXVFLENVIR	H4 52-67 (K59X)
N24	RGVLXVFLENVIRDAV	H4 55-70 (K59X)
O 1	LXVFLENVIRDAVTYT	H4 58-73 (K59X)
O 2	FLENVIRDAVTYTEHA	H4 61-76
O 3	NVIRDAVTYTEHAXRK	H4 64-79 (K77X)
O 4	NVIRDAVTYTEHAKRX	H4 64-79 (K79X)
O 5	RDAVTYTEHAXRKTVT	H4 67-82 (K77X)
O 6	RDAVTYTEHAKRXTVT	H4 67-82 (K79X)
O 7	VTYTEHAXRKTVTAMD	H4 70-85 (K77X)
O 8	VTYTEHAKRXTVTAMD	H4 70-85 (K79X)
O 9	TEHAXRKTVTAMDVVY	H4 73-88 (K77X)
O10	TEHAKRXTVTAMDVVY	H4 73-88 (K79X)
O11	AXRKTVTAMDVVYALK	H4 76-91 (K77X)
O12	AKRXTVTAMDVVYALK	H4 76-91 (K79X)
O13	AKRKTVTAMDVVYALX	H4 76-91 (K91X)

G14	-	O14	XTVTAMDVVYALKRQG	H4 79-94 (K79X)
G15	-	O15	KTVTAMDVVYALXRQG	H4 79-94 (K91X)
G16	-	O16	TAMDVVYALXRQGRTL	H4 82-97 (K91X)
G17	-	O17	DVVYALXRQGRTLYGF	H4 85-100 (K91X)
G18	-	O18	VYALXRQGRTLYGFGG	H4 87-102 (K91X)
G19	-	O19	-	
G20	-	O20	-	
G21	-	O21	-	
G22	-	O22	-	
G23	-	O23	-	
G24	-	O24	-	
H 1	-	P 1	-	
H 2	-	P 2	-	
H 3	-	P 3	-	
H 4	-	P 4	-	
H 5	-	P 5	-	
H 6	-	P 6	-	
H 7	-	P 7	-	
H 8	-	P 8	-	
H 9	-	P 9	-	
H10	-	P10	-	
H11	-	P11	-	
H12	-	P12	-	
H13	-	P13	-	
H14	-	P14	-	
H15	-	P15	-	
H16	-	P16	-	
H17	-	P17	-	
H18	-	P18	-	
H19	-	P19	-	
H20	-	P20	-	
H21	-	P21	-	
H22	-	P22	-	
H23	-	P23	-	
H24	-	P24	-	

Supplementary Table 3. Peptides included in the fine mapping array, based on the sequence of histone 4 (H4) and including Asuha residues (X) in replacement of K12.

Position	Sequence	Name	Position	Sequence	Name
A 1	SGRGKGGKGLGXGGA	H4 1-15 (K12X)	P 1	KGGKGLGXGGAKRHR	H4 5-19 (K12X)
A 2	GRGKGGKGLGXGGAK	H4 2-16 (K12X)	P 2	GGKGLGXGGAKRHR	H4 6-19 (K12X)
A 3	RGKGGKGLGXGGAKR	H4 3-17 (K12X)	P 3	KGGKGLGXGGAKRH	H4 5-18 (K12X)
A 4	GKGGKGLGXGGAKRH	H4 4-18 (K12X)	P 4	GGKGLGXGGAKRH	H4 6-18 (K12X)
A 5	KGGKGLGXGGAKRHR	H4 5-19 (K12X)	P 5	GKGLGXGGAKRH	H4 7-18 (K12X)
A 6	GGKGLGXGGAKRHRK	H4 6-20 (K12X)	P 6	GGKGLGXGGAKR	H4 6-17 (K12X)
A 7	GKGLGXGGAKRHRKV	H4 7-21 (K12X)	P 7	GKGLGXGGAKR	H4 7-17 (K12X)
A 8	KGLGXGGAKRHRKVL	H4 8-22 (K12X)	P 8	KGLGXGGAKR	H4 8-17 (K12X)
A 9	GLGXGGAKRHRKVLR	H4 9-23 (K12X)	P 9	GKGLGXGGAK	H4 7-16 (K12X)
A10	LGXGGAKRHRKVLRD	H4 10-24 (K12)	P10	KGLGXGGAK	H4 8-16 (K12X)
A11	KGGKGLGXGGAKRH	H4 5-18 (K12X)	P11	GLGXGGAK	H4 9-16 (K12X)
A12	KGGKGLGXGGAKR	H4 5-17 (K12X)	P12	KGLGXGGA	H4 8-15 (K12X)
A13	KGGKGLGXGGAK	H4 5-16 (K12X)	P13	GLGXGGA	H4 9-15 (K12X)
A14	KGGKGLGXGGA	H4 5-15 (K12X)	P14	LGXGGA	H4 10-15 (K12X)
A15	KGGKGLGXGG	H4 5-14 (K12X)	P15	GLGXGG	H4 9-14 (K12X)
A16	KGGKGLGXG	H4 5-13 (K12X)	P16	LGXGG	H4 10-14 (K12X)
A17	KGGKGLGX	H4 5-12 (K12X)	P17	GXGG	H4 11-14 (K12X)
A18	GGKGLGXGGAKRHR	H4 6-19 (K12X)	P18	LGXG	H4 10-13 (K12X)
A19	GKGLGXGGAKRHR	H4 7-19 (K12X)	P19	GXG	H4 11-13 (K12X)
A20	KGLGXGGAKRHR	H4 8-19 (K12X)	P20	XG	H4 12-13 (K12X)
A21	GLGXGGAKRHR	H4 9-19 (K12X)	P21	GX	H4 11-12 (K12X)
A22	LGXGGAKRHR	H4 10-19 (K12X)	P22	X	H4 12 (K12X)
A23	GXGGAKRHR	H4 11-19 (K12X)			
A24	XGGAKRHR	H4 12-19 (K12X)			

Supplementary Table 4. Microarray peptide sequences including Asuha (X) surrogates of Kac and reported neighboring histone post-translational modifications.

Pos.	Sequence	Name
A 1	A R T K Q T A R X S T G G K A P	H3 1-16 (K9X)
A 2	A R pT K Q T A R X S T G G K A P	1 PTM
A 3	A R T Kme1 Q T A R X S T G G K A P	
A 4	A R T Kme2 Q T A R X S T G G K A P	
A 5	A R T Kme3 Q T A R X S T G G K A P	
A 6	A R T Kac Q T A R X S T G G K A P	
A 7	A R T Kthioac Q T A R X S T G G K A P	
A 8	A R T K Q pT A R X S T G G K A P	
A 9	A R T K Q T A R X pS T G G K A P	
A10	A R T K Q T A R X S pT G G K A P	
A11	A R T K Q T A R X S T G G Kac A P	
A12	A R T K Q T A R X S T G G Kthioac A P	
A13	A R pT Kme1 Q T A R X S T G G K A P	2 PTMs
A14	A R pT Kme2 Q T A R X S T G G K A P	
A15	A R pT Kme3 Q T A R X S T G G K A P	
A16	A R pT Kac Q T A R X S T G G K A P	
A17	A R pT Kthioac Q T A R X S T G G K A P	
A18	A R pT K Q pT A R X S T G G K A P	
A19	A R pT K Q T A R X pS T G G K A P	
A20	A R pT K Q T A R X S pT G G K A P	
A21	A R pT K Q T A R X S T G G Kac A P	
A22	A R pT K Q T A R X S T G G Kthioac A P	
A23	A R T Kme1 Q pT A R X S T G G K A P	
A24	A R T Kme1 Q T A R X pS T G G K A P	
B 1	A R T Kme1 Q T A R X S pT G G K A P	
B 2	A R T Kme1 Q T A R X S T G G Kac A P	
B 3	A R T Kme1 Q T A R X S T G G Kthioac A P	
B 4	A R T Kme2 Q pT A R X S T G G K A P	
B 5	A R T Kme2 Q T A R X pS T G G K A P	
B 6	A R T Kme2 Q T A R X S pT G G K A P	
B 7	A R T Kme2 Q T A R X S T G G Kac A P	
B 8	A R T Kme2 Q T A R X S T G G Kthioac A P	
B 9	A R T Kme3 Q pT A R X S T G G K A P	
B10	A R T Kme3 Q T A R X pS T G G K A P	
B11	A R T Kme3 Q T A R X S pT G G K A P	
B12	A R T Kme3 Q T A R X S T G G Kac A P	
B13	A R T Kme3 Q T A R X S T G G Kthioac A P	
B14	A R T Kac Q pT A R X S T G G K A P	
B15	A R T Kac Q T A R X pS T G G K A P	
B16	A R T Kac Q T A R X S pT G G K A P	
B17	A R T Kac Q T A R X S T G G Kac A P	
B18	A R T Kac Q T A R X S T G G Kthioac A P	
B19	A R T Kthioac Q pT A R X S T G G K A P	
B20	A R T Kthioac Q T A R X pS T G G K A P	
B21	A R T Kthioac Q T A R X S pT G G K A P	
B22	A R T Kthioac Q T A R X S T G G Kac A P	
B23	A R T Kthioac Q T A R X S T G G Kthioac A P	
B24	A R T K Q pT A R X pS T G G K A P	
C 1	A R T K Q pT A R X S pT G G K A P	
C 2	A R T K Q pT A R X S T G G Kac A P	
I 1	A R pT K Q T A R X pS pT G G Kac A P	
I 2	A R pT K Q T A R X pS pT G G Kthioac A P	
I 3	A R T Kme1 Q pT A R X pS pT G G K A P	
I 4	A R T Kme1 Q pT A R X pS T G G Kac A P	
I 5	A R T Kme1 Q pT A R X pS T G G Kthioac A P	
I 6	A R T Kme1 Q pT A R X S pT G G Kac A P	
I 7	A R T Kme1 Q pT A R X S pT G G Kthioac A P	
I 8	A R T Kme1 Q T A R X pS pT G G Kac A P	
I 9	A R T Kme1 Q T A R X pS pT G G Kthioac A P	
I10	A R T Kme2 Q pT A R X pS pT G G K A P	
I11	A R T Kme2 Q pT A R X pS T G G Kac A P	
I12	A R T Kme2 Q pT A R X pS T G G Kthioac A P	
I13	A R T Kme2 Q pT A R X S pT G G Kac A P	
I14	A R T Kme2 Q pT A R X S pT G G Kthioac A P	
I15	A R T Kme2 Q T A R X pS pT G G Kac A P	
I16	A R T Kme2 Q T A R X pS pT G G Kthioac A P	
I17	A R T Kme3 Q pT A R X pS pT G G K A P	
I18	A R T Kme3 Q pT A R X pS T G G Kac A P	
I19	A R T Kme3 Q pT A R X pS T G G Kthioac A P	
I20	A R T Kme3 Q pT A R X S pT G G Kac A P	
I21	A R T Kme3 Q pT A R X S pT G G Kthioac A P	
I22	A R T Kme3 Q T A R X pS pT G G Kac A P	
I23	A R T Kme3 Q T A R X pS pT G G Kthioac A P	
I24	A R T Kac Q pT A R X pS pT G G K A P	
J 1	A R T Kac Q pT A R X pS T G G Kac A P	
J 2	A R T Kac Q pT A R X pS T G G Kthioac A P	
J 3	A R T Kac Q pT A R X S pT G G Kac A P	
J 4	A R T Kac Q pT A R X S pT G G Kthioac A P	
J 5	A R T Kac Q T A R X pS pT G G Kac A P	
J 6	A R T Kac Q T A R X pS pT G G Kthioac A P	
J 7	A R T Kthioac Q pT A R X pS pT G G K A P	
J 8	A R T Kthioac Q pT A R X pS T G G Kac A P	
J 9	A R T Kthioac Q pT A R X pS T G G Kthioac A P	
J10	A R T Kthioac Q pT A R X S pT G G Kac A P	
J11	A R T Kthioac Q pT A R X S pT G G Kthioac A P	
J12	A R T Kthioac Q T A R X pS pT G G Kac A P	
J13	A R T Kthioac Q T A R X pS pT G G Kthioac A P	
J14	A R T K Q pT A R X pS pT G G Kac A P	
J15	A R T K Q pT A R X pS pT G G Kthioac A P	
J16	A R pT Kme1 Q pT A R X pS pT G G K A P	5 PTMs
J17	A R pT Kme1 Q pT A R X pS T G G Kac A P	
J18	A R pT Kme1 Q pT A R X pS T G G Kthioac A P	
J19	A R pT Kme1 Q pT A R X S pT G G Kac A P	
J20	A R pT Kme1 Q pT A R X S pT G G Kthioac A P	
J21	A R pT Kme1 Q T A R X pS pT G G Kac A P	
J22	A R pT Kme1 Q T A R X pS pT G G Kthioac A P	
J23	A R pT Kme2 Q pT A R X pS pT G G K A P	
J24	A R pT Kme2 Q pT A R X pS T G G Kac A P	
K 1	A R pT Kme2 Q pT A R X pS T G G Kthioac A P	
K 2	A R pT Kme2 Q pT A R X S pT G G Kac A P	

C 3	A R T K Q pT A R X S T G G Kthioac A P	
C 4	A R T K Q T A R X pS pT G G K A P	
C 5	A R T K Q T A R X pS T G G Kac A P	
C 6	A R T K Q T A R X pS T G G Kthioac A P	
C 7	A R T K Q T A R X S pT G G Kac A P	
C 8	A R T K Q T A R X S pT G G Kthioac A P	
C 9	A R pT Kme1 Q pT A R X S T G G K A P	3 PTMs
C10	A R pT Kme1 Q T A R X pS T G G K A P	
C11	A R pT Kme1 Q T A R X S pT G G K A P	
C12	A R pT Kme1 Q T A R X S T G G Kac A P	
C13	A R pT Kme1 Q T A R X S T G G Kthioac A P	
C14	A R pT Kme2 Q pT A R X S T G G K A P	
C15	A R pT Kme2 Q T A R X pS T G G K A P	
C16	A R pT Kme2 Q T A R X S pT G G K A P	
C17	A R pT Kme2 Q T A R X S T G G Kac A P	
C18	A R pT Kme2 Q T A R X S T G G Kthioac A P	
C19	A R pT Kme3 Q pT A R X S T G G K A P	
C20	A R pT Kme3 Q T A R X pS T G G K A P	
C21	A R pT Kme3 Q T A R X S pT G G K A P	
C22	A R pT Kme3 Q T A R X S T G G Kac A P	
C23	A R pT Kme3 Q T A R X S T G G Kthioac A P	
C24	A R pT Kac Q pT A R X S T G G K A P	
D 1	A R pT Kac Q T A R X pS T G G K A P	
D 2	A R pT Kac Q T A R X S pT G G K A P	
D 3	A R pT Kac Q T A R X S T G G Kac A P	
D 4	A R pT Kac Q T A R X S T G G Kthioac A P	
D 5	A R pT Kthioac Q pT A R X S T G G K A P	
D 6	A R pT Kthioac Q T A R X pS T G G K A P	
D 7	A R pT Kthioac Q T A R X S pT G G K A P	
D 8	A R pT Kthioac Q T A R X S T G G Kac A P	
D 9	A R pT Kthioac Q T A R X S T G G Kthioac A P	
D10	A R pT K Q pT A R X pS T G G K A P	
D11	A R pT K Q pT A R X S pT G G K A P	
D12	A R pT K Q pT A R X S T G G Kac A P	
D13	A R pT K Q pT A R X S T G G Kthioac A P	
D14	A R pT K Q T A R X pS pT G G K A P	
D15	A R pT K Q T A R X pS T G G Kac A P	
D16	A R pT K Q T A R X pS T G G Kthioac A P	
D17	A R pT K Q T A R X S pT G G Kac A P	
D18	A R pT K Q T A R X S pT G G Kthioac A P	
D19	A R T Kme1 Q pT A R X pS T G G K A P	
D20	A R T Kme1 Q pT A R X S pT G G K A P	
D21	A R T Kme1 Q pT A R X S T G G Kac A P	
D22	A R T Kme1 Q pT A R X S T G G Kthioac A P	
D23	A R T Kme1 Q T A R X pS pT G G K A P	
D24	A R T Kme1 Q T A R X pS T G G Kac A P	
E 1	A R T Kme1 Q T A R X pS T G G Kthioac A P	
E 2	A R T Kme1 Q T A R X S pT G G Kac A P	
E 3	A R T Kme1 Q T A R X S pT G G Kthioac A P	
E 4	A R T Kme2 Q pT A R X pS T G G K A P	
E 5	A R T Kme2 Q pT A R X S pT G G K A P	
E 6	A R T Kme2 Q pT A R X S T G G Kac A P	
E 7	A R T Kme2 Q pT A R X S T G G Kthioac A P	
E 8	A R T Kme2 Q T A R X pS pT G G K A P	
K 3	A R pT Kme2 Q pT A R X S pT G G Kthioac A P	
K 4	A R pT Kme2 Q T A R X pS pT G G Kac A P	
K 5	A R pT Kme2 Q T A R X pS pT G G Kthioac A P	
K 6	A R pT Kme3 Q pT A R X pS pT G G K A P	
K 7	A R pT Kme3 Q pT A R X pS T G G Kac A P	
K 8	A R pT Kme3 Q pT A R X pS T G G Kthioac A P	
K 9	A R pT Kme3 Q pT A R X S pT G G Kac A P	
K10	A R pT Kme3 Q pT A R X S pT G G Kthioac A P	
K11	A R pT Kme3 Q T A R X pS pT G G Kac A P	
K12	A R pT Kme3 Q T A R X pS pT G G Kthioac A P	
K13	A R pT Kac Q pT A R X pS pT G G K A P	
K14	A R pT Kac Q pT A R X pS T G G Kac A P	
K15	A R pT Kac Q pT A R X pS T G G Kthioac A P	
K16	A R pT Kac Q pT A R X S pT G G Kac A P	
K17	A R pT Kac Q pT A R X S pT G G Kthioac A P	
K18	A R pT Kac Q T A R X pS pT G G Kac A P	
K19	A R pT Kac Q T A R X pS pT G G Kthioac A P	
K20	A R pT Kthioac Q pT A R X pS pT G G K A P	
K21	A R pT Kthioac Q pT A R X pS T G G Kac A P	
K22	A R pT Kthioac Q pT A R X pS T G G Kthioac A P	
K23	A R pT Kthioac Q pT A R X S pT G G Kac A P	
K24	A R pT Kthioac Q pT A R X S pT G G Kthioac A P	
L 1	A R pT Kthioac Q T A R X pS pT G G Kac A P	
L 2	A R pT Kthioac Q T A R X pS pT G G Kthioac A P	
L 3	A R pT K Q pT A R X pS pT G G Kac A P	
L 4	A R pT K Q pT A R X pS pT G G Kthioac A P	
L 5	A R T Kme1 Q pT A R X pS pT G G Kac A P	
L 6	A R T Kme1 Q pT A R X pS pT G G Kthioac A P	
L 7	A R T Kme2 Q pT A R X pS pT G G Kac A P	
L 8	A R T Kme2 Q pT A R X pS pT G G Kthioac A P	
L 9	A R T Kme3 Q pT A R X pS pT G G Kac A P	
L10	A R T Kme3 Q pT A R X pS pT G G Kthioac A P	
L11	A R T Kac Q pT A R X pS pT G G Kac A P	
L12	A R T Kac Q pT A R X pS pT G G Kthioac A P	
L13	A R T Kthioac Q pT A R X pS pT G G Kac A P	
L14	A R T Kthioac Q pT A R X pS pT G G Kthioac A P	
L15	A R pT Kme1 Q pT A R X pS pT G G Kac A P	6 PTMs
L16	A R pT Kme1 Q pT A R X pS pT G G Kthioac A P	
L17	A R pT Kme2 Q pT A R X pS pT G G Kac A P	
L18	A R pT Kme2 Q pT A R X pS pT G G Kthioac A P	
L19	A R pT Kme3 Q pT A R X pS pT G G Kac A P	
L20	A R pT Kme3 Q pT A R X pS pT G G Kthioac A P	
L21	A R pT Kac Q pT A R X pS pT G G Kac A P	
L22	A R pT Kac Q pT A R X pS pT G G Kthioac A P	
L23	A R pT Kthioac Q pT A R X pS pT G G Kac A P	
L24	A R pT Kthioac Q pT A R X pS pT G G Kthioac A P	
M 1	S T G G K A P R X Q L A T K A A	H3 10-25 (K18X)
M 2	pS T G G K A P R X Q L A T K A A	1 PTM
M 3	S pT G G K A P R X Q L A T K A A	
M 4	S T G G Kac A P R X Q L A T K A A	
M 5	S T G G Kthioac A P R X Q L A T K A A	
M 6	S T G G K A P R X Q L A T Kme1 A A	
M 7	S T G G K A P R X Q L A T Kac A A	
M 8	S T G G K A P R X Q L A T Kthioac A A	

E 9	A R T Kme2 Q T A R X pS T G G Kac A P	
E10	A R T Kme2 Q T A R X pS T G G Kthioac A P	
E11	A R T Kme2 Q T A R X S pT G G Kac A P	
E12	A R T Kme2 Q T A R X S pT G G Kthioac A P	
E13	A R T Kme3 Q pT A R X pS T G G K A P	
E14	A R T Kme3 Q pT A R X S pT G G K A P	
E15	A R T Kme3 Q pT A R X S T G G Kac A P	
E16	A R T Kme3 Q pT A R X S T G G Kthioac A P	
E17	A R T Kme3 Q T A R X pS pT G G K A P	
E18	A R T Kme3 Q T A R X pS T G G Kac A P	
E19	A R T Kme3 Q T A R X pS T G G Kthioac A P	
E20	A R T Kme3 Q T A R X S pT G G Kac A P	
E21	A R T Kme3 Q T A R X S pT G G Kthioac A P	
E22	A R T Kac Q pT A R X pS T G G K A P	
E23	A R T Kac Q pT A R X S pT G G K A P	
E24	A R T Kac Q pT A R X S T G G Kac A P	
F 1	A R T Kac Q pT A R X S T G G Kthioac A P	
F 2	A R T Kac Q T A R X pS pT G G K A P	
F 3	A R T Kac Q T A R X pS T G G Kac A P	
F 4	A R T Kac Q T A R X pS T G G Kthioac A P	
F 5	A R T Kac Q T A R X S pT G G Kac A P	
F 6	A R T Kac Q T A R X S pT G G Kthioac A P	
F 7	A R T Kthioac Q pT A R X pS T G G K A P	
F 8	A R T Kthioac Q pT A R X S pT G G K A P	
F 9	A R T Kthioac Q pT A R X S T G G Kac A P	
F10	A R T Kthioac Q pT A R X S T G G Kthioac A P	
F11	A R T Kthioac Q T A R X pS pT G G K A P	
F12	A R T Kthioac Q T A R X pS T G G Kac A P	
F13	A R T Kthioac Q T A R X pS T G G Kthioac A P	
F14	A R T Kthioac Q T A R X S pT G G Kac A P	
F15	A R T Kthioac Q T A R X S pT G G Kthioac A P	
F16	A R T K Q pT A R X pS pT G G K A P	
F17	A R T K Q pT A R X pS T G G Kac A P	
F18	A R T K Q pT A R X pS T G G Kthioac A P	
F19	A R T K Q pT A R X S pT G G Kac A P	
F20	A R T K Q pT A R X S pT G G Kthioac A P	
F21	A R T K Q T A R X pS pT G G Kac A P	
F22	A R T K Q T A R X pS pT G G Kthioac A P	
F23	A R pT Kme1 Q pT A R X pS T G G K A P	4 PTMs
F24	A R pT Kme1 Q pT A R X S pT G G K A P	
G 1	A R pT Kme1 Q pT A R X S T G G Kac A P	
G 2	A R pT Kme1 Q pT A R X S T G G Kthioac A P	
G 3	A R pT Kme1 Q T A R X pS pT G G K A P	
G 4	A R pT Kme1 Q T A R X pS T G G Kac A P	
G 5	A R pT Kme1 Q T A R X pS T G G Kthioac A P	
G 6	A R pT Kme1 Q T A R X S pT G G Kac A P	
G 7	A R pT Kme1 Q T A R X S pT G G Kthioac A P	
G 8	A R pT Kme2 Q pT A R X pS T G G K A P	
G 9	A R pT Kme2 Q pT A R X S pT G G K A P	
G10	A R pT Kme2 Q pT A R X S T G G Kac A P	
G11	A R pT Kme2 Q pT A R X S T G G Kthioac A P	
G12	A R pT Kme2 Q T A R X pS pT G G K A P	
G13	A R pT Kme2 Q T A R X pS T G G Kac A P	
G14	A R pT Kme2 Q T A R X pS T G G Kthioac A P	

M 9	pS pT G G K A P R X Q L A T K A A	2 PTMs
M10	pS T G G Kac A P R X Q L A T K A A	
M11	pS T G G Kthioac A P R X Q L A T K A A	
M12	pS T G G K A P R X Q L A T Kme1 A A	
M13	pS T G G K A P R X Q L A T Kac A A	
M14	pS T G G K A P R X Q L A T Kthioac A A	
M15	S pT G G Kac A P R X Q L A T K A A	
M16	S pT G G Kthioac A P R X Q L A T K A A	
M17	S pT G G K A P R X Q L A T Kme1 A A	
M18	S pT G G K A P R X Q L A T Kac A A	
M19	S pT G G K A P R X Q L A T Kthioac A A	
M20	S T G G Kac A P R X Q L A T Kme1 A A	
M21	S T G G Kac A P R X Q L A T Kac A A	
M22	S T G G Kac A P R X Q L A T Kthioac A A	
M23	S T G G Kthioac A P R X Q L A T Kme1 A A	
M24	S T G G Kthioac A P R X Q L A T Kac A A	
N 1	S T G G Kthioac A P R X Q L A T Kthioac A A	
N 2	pS pT G G Kac A P R X Q L A T K A A	3 PTMs
N 3	pS pT G G Kthioac A P R X Q L A T K A A	
N 4	pS pT G G K A P R X Q L A T Kme1 A A	
N 5	pS pT G G K A P R X Q L A T Kac A A	
N 6	pS pT G G K A P R X Q L A T Kthioac A A	
N 7	pS T G G Kac A P R X Q L A T Kme1 A A	
N 8	pS T G G Kac A P R X Q L A T Kac A A	
N 9	pS T G G Kac A P R X Q L A T Kthioac A A	
N10	pS T G G Kthioac A P R X Q L A T Kme1 A A	
N11	pS T G G Kthioac A P R X Q L A T Kac A A	
N12	pS T G G Kthioac A P R X Q L A T Kthioac A A	
N13	S pT G G Kac A P R X Q L A T Kme1 A A	
N14	S pT G G Kac A P R X Q L A T Kac A A	
N15	S pT G G Kac A P R X Q L A T Kthioac A A	
N16	S pT G G Kthioac A P R X Q L A T Kme1 A A	
N17	S pT G G Kthioac A P R X Q L A T Kac A A	
N18	S pT G G Kthioac A P R X Q L A T Kthioac A A	
N19	pS pT G G Kac A P R X Q L A T Kme1 A A	4 PTMs
N20	pS pT G G Kac A P R X Q L A T Kac A A	
N21	pS pT G G Kac A P R X Q L A T Kthioac A A	
N22	pS pT G G Kthioac A P R X Q L A T Kme1 A A	
N23	pS pT G G Kthioac A P R X Q L A T Kac A A	
N24	pS pT G G Kthioac A P R X Q L A T Kthioac A A	
O 1	G K G G K G L G X G G A K R H R	H4 4-19 (K12X)
O 2	G Kac G G K G L G X G G A K R H R	1 PTM
O 3	G Kthioac G G K G L G X G G A K R H R	
O 4	G K G G Kac G L G X G G A K R H R	
O 5	G K G G Kthioac G L G X G G A K R H R	
O 6	G K G G K G L G X G G A Kac R H R	
O 7	G K G G K G L G X G G A Kthioac R H R	
O 8	G Kac G G Kac G L G X G G A K R H R	2 PTMs
O 9	G Kac G G Kthioac G L G X G G A K R H R	
O10	G Kac G G K G L G X G G A Kac R H R	
O11	G Kac G G K G L G X G G A Kthioac R H R	
O12	G Kthioac G G Kac G L G X G G A K R H R	
O13	G Kthioac G G Kthioac G L G X G G A K R H R	
O14	G Kthioac G G K G L G X G G A Kac R H R	

G15	A R pT Kme2 Q T A R X S pT G G Kac A P	O15	G Kthioac G G K G L G X G G A Kthioac R H R	
G16	A R pT Kme2 Q T A R X S pT G G Kthioac A P	O16	G K G G Kac G L G X G G A Kac R H R	
G17	A R pT Kme3 Q pT A R X pS T G G K A P	O17	G K G G Kac G L G X G G A Kthioac R H R	
G18	A R pT Kme3 Q pT A R X S pT G G K A P	O18	G K G G Kthioac G L G X G G A Kac R H R	
G19	A R pT Kme3 Q pT A R X S T G G Kac A P	O19	G K G G Kthioac G L G X G G A Kthioac R H R	
G20	A R pT Kme3 Q pT A R X S T G G Kthioac A P	O20	G Kac G G Kac G L G X G G A Kac R H R	3 PTMs
G21	A R pT Kme3 Q T A R X pS pT G G K A P	O21	G Kac G G Kac G L G X G G A Kthioac R H R	
G22	A R pT Kme3 Q T A R X pS T G G Kac A P	O22	G Kac G G Kthioac G L G X G G A Kac R H R	
G23	A R pT Kme3 Q T A R X pS T G G Kthioac A P	O23	G Kac G G Kthioac G L G X G G A Kthioac R H R	
G24	A R pT Kme3 Q T A R X S pT G G Kac A P	O24	G Kthioac G G Kac G L G X G G A Kac R H R	
H 1	A R pT Kme3 Q T A R X S pT G G Kthioac A P	P 1	G Kthioac G G Kac G L G X G G A Kthioac R H R	
H 2	A R pT Kac Q pT A R X pS T G G K A P	P 2	G Kthioac G G Kthioac G L G X G G A Kac R H R	
H 3	A R pT Kac Q pT A R X S pT G G K A P	P 3	G Kthioac G G Kthioac G L G X G G A Kthioac R H R	
H 4	A R pT Kac Q pT A R X S T G G Kac A P	P 4	L G K G G A X R H R K V L R D N	H4 10-25 (K16X)
H 5	A R pT Kac Q pT A R X S T G G Kthioac A P	P 5	L G Kac G G A X R H R K V L R D N	1 PTM
H 6	A R pT Kac Q T A R X pS pT G G K A P	P 6	L G Kthioac G G A X R H R K V L R D N	
H 7	A R pT Kac Q T A R X pS T G G Kac A P	P 7	L G K G G A X R H R Kme1 V L R D N	
H 8	A R pT Kac Q T A R X pS T G G Kthioac A P	P 8	L G K G G A X R H R Kme2 V L R D N	
H 9	A R pT Kac Q T A R X S pT G G Kac A P	P 9	L G K G G A X R H R Kme3 V L R D N	
H10	A R pT Kac Q T A R X S pT G G Kthioac A P	P10	L G Kac G G A X R H R Kme1 V L R D N	2 PTMs
H11	A R pT Kthioac Q pT A R X pS T G G K A P	P11	L G Kac G G A X R H R Kme2 V L R D N	
H12	A R pT Kthioac Q pT A R X S pT G G K A P	P12	L G Kac G G A X R H R Kme3 V L R D N	
H13	A R pT Kthioac Q pT A R X S T G G Kac A P	P13	L G Kthioac G G A X R H R Kme1 V L R D N	
H14	A R pT Kthioac Q pT A R X S T G G Kthioac A P	P14	L G Kthioac G G A X R H R Kme2 V L R D N	
H15	A R pT Kthioac Q T A R X pS pT G G K A P	P15	L G Kthioac G G A X R H R Kme3 V L R D N	
H16	A R pT Kthioac Q T A R X pS T G G Kac A P	P16	-	
H17	A R pT Kthioac Q T A R X pS T G G Kthioac A P	P17	-	
H18	A R pT Kthioac Q T A R X S pT G G Kac A P	P18	-	
H19	A R pT Kthioac Q T A R X S pT G G Kthioac A P	P19	-	
H20	A R pT K Q pT A R X pS pT G G K A P	P20	-	
H21	A R pT K Q pT A R X pS T G G Kac A P	P21	-	
H22	A R pT K Q pT A R X pS T G G Kthioac A P	P22	-	
H23	A R pT K Q pT A R X S pT G G Kac A P	P23	-	
H24	A R pT K Q pT A R X S pT G G Kthioac A P	P24	-	

Supplementary Methods

All reagents were of analytical grade and they were used as obtained from commercial suppliers without further purification. Anhydrous CH_2Cl_2 was obtained from an in-house PureSolv system and other anhydrous solvents were purchased. Reactions were monitored by HPLC-MS analysis or by thin-layer chromatography (TLC) using silica gel-coated plates (analytical SiO_2-60, F-254) and UV light or standard $KMnO_4$ visualization. Column chromatography purification was performed on a dry column vacuum chromatography setup using granular silica gel (60 Å pore size, 15–40 μm) as stationary phase and EtOAc/heptane mixtures as eluent. ^{1}H NMR and ^{13}C NMR were recorded on a Bruker Avance III HD equipped with a cryogenically cooled probe, at 600 MHz and 151 MHz, respectively. Chemical shifts are reported in ppm relative to deuterated solvent as internal standard, and NMR spectra assignments are based on correlation spectroscopy (i.e. COSY, HSQC, HMBC). Peptides were purified by preparative HPLC on an Agilent 1260 LC system equipped with a C18 Phenomenex Luna column [5 μm, 100 Å, 250 × 20 mm] and a diode array UV detector. Flow rate was 20 mL/min, linear gradients of eluent B (0.1% TFA in CH_3CN) in eluent A [0.1% TFA in MilliQ H_2O/CH_3CN, 95:5 (v/v)] over 30 min were applied, and fractions containing the desired peptide were identified by HPLC-MS or MALDI-TOF, lyophilized, and analyzed by analytical HPLC or UPLC. HPLC-MS analyses were performed on a Waters Acquity ultra high-performance liquid chromatography system equipped with a C18 Phenomenex Kinetex column [50 × 2.1 mm, 1.7 μm, 100 Å] and PDA and SQ detectors, using a 0–95% linear gradient of eluent II (0.1% HCOOH in MeCN) in eluent I (0.1% HCOOH in MilliQ H_2O) during t = 0.20−4.80 min at 0.6 mL/min flow rate unless otherwise stated. MALDI-TOF mass spectra were recorded on a Bruker Microflex bench-top MALDI using alpha-cyano-4-hydroxycinnamic acid (CHCA) in CH_3CN/H_2O [50:50 with 0.1% TFA (v/v)] as matrix. Analytical reversed-phase HPLC/UPLC was performed on either (1) an Agilent 1100 HPLC system equipped with a C18 Phenomenex Kinetex column [150 × 4.60 mm, 2.6 μm, 100 Å] or a C8 Phenomenex Kinetex column [250 × 4.6 mm, 5 μm, 100 Å] and a diode array UV detector, using a 0–95% linear gradient of eluent B in eluent A during t = 5−35 min, with a flow rate of 1 mL/min; or (2) on an Agilent 1260 Infinity II series 'UPLC' system equipped with a C18 Infinity Poroshell 120 column [100 × 3.0 mm, 2.7 μm] and a diode array UV detector, using a 0–50% linear gradient of eluent B in eluent A during t = 1–11 min, with a flow rate of 1.2 mL/min at 40 °C. High-resolution mass spectrometry (HRMS) was performed on a Bruker SolariX ESI instrument or on a QExactive Orbitrap mass spectrometer equipped with a SMALDI5 ion source. Assays were performed in 4-(2-hydroxyethyl)-1-piperazineethanesulfonic acid (HEPES) buffer [50 mM HEPES/Na, 100 mM KCl, 0.001% (v/v) tween-20, 0.2 mM tris(2-carboxyethyl)phosphine (TCEP), 0.5 mg/mL bovine serum albumin (BSA), pH 7.4] unless otherwise stated. Phosphate buffered saline (PBS) was prepared from soluble tablets (Sigma-Aldrich, cat. #: 79382-50TAB).

Asuha Synthesis. *6-Bromo-N-(tert-butoxy)-N-(tert-butoxycarbonyl)hexanamide* (**S1**). *Step 1.* O-*tert*-butylhydroxylamine hydrochloride (2.58 g, 20.6 mmol, 1.1 equiv) and *N*,*N*-diisopropylethylamine (6.5 mL, 37.4 mmol, 2.0 equiv) were mixed in anhydrous CH_2Cl_2 (20 mL) at 0 °C under N_2 atm, and 6-bromohexanoyl chloride (2.9 mL, 18.7 mmol, 1.0 equiv) was added dropwise during 5 min (gas development was observed). The reaction was allowed to stir at room temperature for 2 h and, thereafter, the orange mixture was diluted with CH_2Cl_2 (150 mL) and washed with aqueous HCl (0.1 M, 100 mL), sat. aqueous $NaHCO_3$ (100 mL), and brine (100 mL). The resulting organic layer was

dried over Na_2SO_4, filtered, and concentrated *in vacuo*. The resulting orange oil (5.00 g) was tentatively assigned to a non-separable mixture of 6-bromo-*N*-(*tert*-butoxy)hexanamide, 6-chloro-*N*-(*tert*-butoxy)hexanamide and CH_2Cl_2 (1:0.26:0.19), and used without further purification. *6-Bromo-N-(tert-butoxy)hexanamide*: ^{1}H NMR (400 MHz, $CDCl_3$): δ 7.61 (s, 1H, NH), 3.40 (t, J = 6.7 Hz, 2H, $BrCH_2$), 2.52–2.29 (m, 1H, $CH_{2,a}CO$), 2.29–1.98 (m, 1H, $CH_{2,b}CO$), 1.87 (p, J = 6.3 Hz, 2H, $BrCH_2C\underline{H_2}$), 1.68 (p, J = 7.2 Hz, 2H, $C\underline{H_2}CH_2CO$), 1.60–1.43 (m, 2H, $C\underline{H_2}CH_2CH_2CO$), 1.26 (s, 9H, $C(CH_3)_3$). HRMS (ESI$^+$): *m*/*z* = 288.05775 [M+Na]$^+$ (calcd. $C_{10}H_{20}BrNNaO_2^+$: 288.05696). *6-Chloro-N-(tert-butoxy)hexanamide*: ^{1}H NMR (400 MHz, $CDCl_3$): δ 7.61 (s, 1H, NH), 3.53 (t, J = 6.6 Hz, 2H, $ClCH_2$), 2.52–2.29 (m, 1H, $CH_{2,a}CO$), 2.29–1.98 (m, 1H, $CH_{2,b}CO$), 1.79 (p, J = 6.8 Hz, 2H, $ClCH_2C\underline{H_2}$), 1.68 (p, J = 7.2 Hz, 2H, $C\underline{H_2}CH_2CO$), 1.60–1.43 (m, 2H, $C\underline{H_2}CH_2CH_2CO$), 1.26 (s, 9H, $C(CH_3)_3$). HRMS (ESI$^+$): *m*/*z* = 222.12613 [M+H]$^+$ (calcd. $C_{10}H_{21}ClNO_2^+$: 222.12553).

Step 2. The previous mixture (<18.7 mmol, 1.0 equiv) and di-*tert*-butyl dicarbonate (7.19 g, 33.0 mmol, 1.8 equiv) were dissolved in THF (50 mL) at room temperature under N_2 atm. 4-Dimethylaminopyridine (DMAP, 457 mg, 3.74 mmol, 0.2 equiv) was added as a THF solution (15 mL), followed by Et_3N (2.6 mL, 18.7 mmol, 1.0 equiv). The mixture was allowed to react overnight, after which the mixture was concentrated *in vacuo*. The resulting orange oil was purified by column chromatography (0–60% EtOAc in heptane), affording a non-separable mixture of the title compound and 6-chloro-*N*-(*tert*-butoxy)-*N*-(*tert*-butoxycarbonyl)hexanamide as clear oil (7.94 g, 58% and 16% respectively). Duplicated NMR peaks are tentatively assigned to cis/trans amide rotamers. Compound **S1**: ^{1}H NMR (600 MHz, $CDCl_3$): δ 3.40 (t, J = 6.8 Hz, 2H, $BrCH_2$), 2.55 (t, J = 7.3 Hz, 0.6H, $CH_{2,rot1}CO$), 2.32 (t, J = 7.3 Hz, 1.4H, $CH_{2,rot2}CO$), 1.88 (p, J = 7.0 Hz, 2H, $BrCH_2C\underline{H_2}$), 1.64–1.58 (m, 2H, $C\underline{H_2}CH_2CO$), 1.55–1.45 (m, 2H, $C\underline{H_2}CH_2CH_2CO$), 1.51 (s, 9H, $COC(CH_3)_3$), 1.26 (s, 9H, $NOC(CH_3)_3$); ^{13}C NMR (151 MHz, $CDCl_3$): δ 157.5, 150.7 (NCO_2), 148.5, 148.2 ($CH_2\underline{C}O$), 84.0, 83.7 ($CO\underline{C}(CH_3)_3$), 79.5, 79.4 ($NO\underline{C}(CH_3)_3$), 33.7 ($BrCH_2$), 32.6 ($BrCH_2\underline{C}H_2$), 31.1, 27.1 ($\underline{C}H_2CO$), 27.8, 27.7, 27.5, 27.5, 27.4 ($COC(\underline{C}H_3)_3$/$NOC(\underline{C}H_3)_3$), 24.5, 24.4, 23.9 ($\underline{C}H_2CH_2CH_2CO$/$\underline{C}H_2CH_2CO$). HRMS (ESI$^+$): *m*/*z* = 388.11126 [M+Na]$^+$ (calcd. $C_{15}H_{28}BrNNaO_4^+$: 388.10939). *6-Chloro-N-(tert-butoxy)-N-(tert-butoxycarbonyl)hexanamide*: ^{1}H NMR (600 MHz, $CDCl_3$): δ 3.53 (t, J = 6.7 Hz, 2H, $ClCH_2$), 2.55 (t, J = 7.3 Hz, 0.6H, $CH_{2,rot1}CO$), 2.32 (t, J = 7.3 Hz, 1.4H, $CH_{2,rot2}CO$), 1.79 (p, J = 7.1 Hz, 2H, $ClCH_2C\underline{H_2}$), 1.64–1.58 (m, 2H, $C\underline{H_2}CH_2CO$), 1.55–1.45 (m, 2H, $C\underline{H_2}CH_2CH_2CO$), 1.51 (s, 9H, $COC(CH_3)_3$), 1.26 (s, 9H, $NOC(CH_3)_3$); ^{13}C NMR (151 MHz, $CDCl_3$): δ 157.5, 150.7 (NCO_2), 148.5, 148.2 ($CH_2\underline{C}O$), 84.0, 83.7 ($CO\underline{C}(CH_3)_3$), 79.5, 79.4 ($NO\underline{C}(CH_3)_3$), 45.0 ($ClCH_2$), 32.4 ($ClCH_2\underline{C}H_2$), 31.1, 27.1 ($\underline{C}H_2CO$), 27.8, 27.7, 27.5, 27.5, 27.4 ($COC(\underline{C}H_3)_3$/$NOC(\underline{C}H_3)_3$), 24.5, 24.4, 23.9 ($\underline{C}H_2CH_2CH_2CO$/$\underline{C}H_2CH_2CO$). HRMS (ESI$^+$): *m*/*z* = 344.16167 [M+Na]$^+$ (calcd. $C_{15}H_{28}ClNNaO_4^+$: 344.15991).

Diethyl 2-acetamido-2-(6-(tert-butoxy(tert-butoxycarbonyl)amino)-6-oxohexyl)malonate (**S3**). *Step 1.* Compound **S1** (7.94 g of the previous mixture, 13.8 mmol, 1.0 equiv) and NaI (3.90 g, 40.0 mmol, 1.9 equiv) were suspended in acetone (50 mL), and the reaction mixture was stirred at reflux under N_2 atm overnight. The resulting orange solution was concentrated *in vacuo*, dissolved in diethyl ether (250 mL) and washed with H_2O (2 × 100 mL). The aqueous layers were extracted with diethyl ether (20 mL), and the combined organic layer was washed with sat aqueous $Na_2S_2O_3$ (100 mL) and brine (150 mL), dried over Na_2SO_4, filtered, and concentrated *in vacuo* to afford 6-iodo-*N*-(*tert*-butoxy)-*N*-(*tert*-butoxycarbonyl)hexanamide (**S2**) as yellow oil (5.51 g).

Step 2. A mixture of NaH (60% dispersion in mineral oil, 0.59 g, 14.7 mmol, 1.1 equiv) in anhydrous DMF (10 mL) was cooled to 0 °C under N_2 atm, and a solution of diethyl acetamidomalonate (3.19 g, 14.7 mmol, 1.1 equiv) in anhydrous DMF (23 mL) was added dropwise during 10 min. Then, the mixture was allowed to stir for 30 min at room temperature, after which compound **S2** (5.51 g, 13.3 mmol, 1.0 equiv) was added in DMF (15 mL). The reaction was stirred overnight at room temperature, and a color change from yellow to orange was observed. Then, the mixture was concentrated *in vacuo* and purified by column chromatography (0–40% EtOAc in heptane), to afford the title compound as orange oil (5.83 g, 84%). ^{1}H NMR (600 MHz, $CDCl_3$): δ 6.75 (s, 1H, NH), 4.24 (q, J = 7.1 Hz, 4H, CO_2CH_2), 2.36–2.29 (m, 2H, CCH_2), 2.26 (t, J = 7.4 Hz, 2H, CH_2CON), 2.03 (s, 3H, $C\underline{H_3}CONH$), 1.59–1.52 (m, 2H, $CCH_2C\underline{H_2}$), 1.51 (m, 9H, $COC(CH_3)_3$), 1.42–1.33 (m, 2H, $CCH_2CH_2C\underline{H_2}$), 1.27–1.21 (m, 6H, $CH_2C\underline{H_3}$), 1.25 (s, 9H, $NOC(CH_3)_3$), 1.14–1.08 (m, 2H, $C\underline{H_2}CH_2CON$); ^{13}C NMR (151 MHz, $CDCl_3$) δ 169.0 ($CH_3\underline{C}ONH$), 168.4 ($\underline{C}O_2CH_2CH_3$), 148.5 ($CH_2\underline{C}ON$/NCOO), 148.4 ($CH_2\underline{C}ON$/NCOO), 83.6 ($CO\underline{C}(CH_3)_3$), 79.3 ($NO\underline{C}(CH_3)_3$), 66.7 ($\underline{C}CH_2$), 62.6 ($CO_2\underline{C}H_2$), 32.1 ($C\underline{C}H_2$), 31.1 ($\underline{C}H_2CON$), 28.6 ($CCH_2CH_2\underline{C}H_2$), 27.7 ($COC(\underline{C}H_3)_3$), 27.4 ($NOC(\underline{C}H_3)_3$), 25.0 ($CCH_2\underline{C}H_2$), 23.5 ($\underline{C}H_2CH_2CO$), 23.2 ($\underline{C}H_3CON$), 14.1 ($CH_2\underline{C}H_3$). HRMS (ESI$^+$): m/z = 503.29754 $[M+H]^+$ (calcd. $C_{24}H_{43}N_2O_9^+$: 503.29631).

(S)-2-((((9H-Fluoren-9-yl)methoxy)carbonyl)amino)-8-(tert-butoxyamino)-8-oxooctanoic acid (**S5**). *Step 1*. Compound **S3** (5.70 g, 11.3 mmol, 1.0 equiv) was suspended in ethanol/H_2O (2:1, 45 mL), and aqueous NaOH (2.0 M, 10 mL) was added to the mixture, which was stirred for 4.5 h until consumption of the starting material (as judged by HPLC-MS). Thereafter, the solution was acidified to pH 4 with aqueous HCl (35%) and stirred at reflux overnight. Then, the mixture was basified with aqueous NaOH (2.0 M, 10 mL), stirred at room temperature for additional 1 h until no ethyl ester remained, and concentrated *in vacuo* to afford 2-acetamido-8-(*tert*-butoxyamino)-8-oxooctanoic acid (**S4**, tentatively identified by HPLC-MS), which was used without further purification.

Step 2. The previous mixture was diluted in phosphate buffer (0.1 M, pH 7.2, 150 mL), adjusted to pH 7.2 with aqueous HCl (2.0 M), and warmed up to 40 °C. A control NMR sample was collected (50 µL crude solution in 400 µL of CD_3OD), Acylase I from *Aspergillus melleus* (426 mg, >0.5 U/mg, Sigma-Aldrich cat. # 01818) and $CoCl_2{\cdot}6H_2O$ (38 mg, final concentration: 10^{-3} M) were added, and the mixture was stirred at 40 °C overnight. The reaction was followed by ^{1}H NMR with water suppression of aliquots of the reaction mixed in CD_3OD as before. Resolution was considered complete when the integration ratio between the signals corresponding to CH (δ: 4.22) and CH_2CO (δ: 2.13) was 50% relative to control. Then, pH was adjusted to 10 with aqueous NaOH (2.0 M) and Fmoc *N*-hydroxysuccinimide ester (FmocOSu, 2.86 g, 8.5 mmol, 0.8 equiv) in 1,4-dioxane (20 mL) was added. The mixture (which turned white) was allowed to react overnight at room temperature, and then the 1,4-dioxane was removed *in vacuo*. The mixture was acidified to pH 3.0 with aqueous HCl (2.0 M) and extracted with EtOAc (2 × 100 mL + 2 × 50 mL). The combined organic layer was washed with brine (150 mL), dried over Na_2SO_4, filtered, and concentrated *in vacuo*. Column chromatography (0–100% EtOAc in heptane, with 0.25% acetic acid) and co-evaporation in toluene/heptane (1:1) afforded the title compound as an off-white solid (2.02 g, 32% relative to the desired enantiomer). ^{1}H NMR (600 MHz, DMSO-d_6): δ 12.49 (s, 1H, CO_2H), 10.20 (s, 1H, $CH_2CON\underline{H}$), 7.89 (d, J = 7.6 Hz, 2H, C4H, C5H), 7.72 (dd, J = 7.4, 2.6 Hz, 2H, C1H, C8H), 7.61 (d, J = 8.1 Hz, 1H, N$\underline{H}$CH), 7.42 (t, J = 7.4 Hz, 2H, C3H, C6H), 7.33 (t, J = 7.4 Hz, 2H, C2H, C7H), 4.34–4.25 (m, 2H, $CHC\underline{H_2}O$), 4.24–4.20 (m, 1H, $C\underline{H}CH_2O$), 3.91 (td, J = 9.3, 4.7 Hz, 1H, $NHC\underline{H}$), 1.99 (t, J = 7.2 Hz, 2H, CH_2CON), 1.72–1.64 (m, 1H,

NHCHC$\underline{\mathrm{H}}_{2,a}$), 1.63–1.55 (m, 1H, NHCHC$\underline{\mathrm{H}}_{2,b}$), 1.53–1.44 (m, 2H, C$\underline{\mathrm{H}}_2CH_2$CON), 1.36–1.28 (m, 2H, CHCH$_2$C$\underline{\mathrm{H}}_2$), 1.28–1.20 (m, 2H, CHCH$_2$CH$_2$C$\underline{\mathrm{H}}_2$), 1.13 (s, 9H, C(CH$_3$)$_3$); ^{13}C NMR (151 MHz, DMSO-*d*$_6$): *δ* 174.0 (CO$_2$H), 170.4 (CH$_2\underline{\mathrm{C}}$ONH), 156.1 (OCONH), 143.8 (C8a or C9a), 143.8 (C8a or C9a), 140.7 (C4a or C4b), 140.7 (C4a or C4b), 127.6 (C3, C6), 127.0 (C2, C7), 125.3 (C1, C8), 120.1 (C4, C5), 80.1 ($\underline{\mathrm{C}}$(CH$_3$)$_3$), 65.6 (CH$\underline{\mathrm{C}}$H$_2$O), 53.7 (NHCH), 46.7 ($\underline{\mathrm{C}}$HCH$_2$O), 32.4 ($\underline{\mathrm{C}}$H$_2$CON), 30.6 (NHCH$\underline{\mathrm{C}}$H$_2$), 28.1 (CHCH$_2$CH$_2\underline{\mathrm{C}}$H$_2$), 26.4 (C($\underline{\mathrm{C}}$H$_3$)$_3$), 25.3 (CHCH$_2\underline{\mathrm{C}}$H$_2$), 25.0 ($\underline{\mathrm{C}}$H$_2$CH$_2$CON). HRMS (ESI$^+$): *m*/*z* = 483.24999 [M+H]$^+$ (calcd. $C_{27}H_{35}N_2O_6^+$: 483.24896), 505.23197 [M+Na]$^+$ (calcd. $C_{27}H_{34}N_2NaO_6^+$: 505.23091.

Asuapa Synthesis. *Ethyl 2-acetamido-8-((2-((tert-butoxycarbonyl)amino)phenyl)amino)-8-oxooctanoate* (**S6**). 8-(*tert*-Butyl) 1-ethyl 2-acetamidooctanedioate[2] (4.96 g, 15.7 mmol, 1.0 equiv) was dissolved in CH_2Cl_2 (40 mL) at 0 °C and treated with TFA (20 mL) under N_2 atmosphere, allowing the mixture to reach room temperature and stir for 2.5 hours. Then, crude reaction was concentrated *in vacuo* and residual TFA was removed by co-evaporation with toluene (3 × 100 mL). The resulting orange oil (5.52 g) was dissolved in CH_2Cl_2 (100 mL) together with *N*-Boc-1,2-phenylenediamine (4.92 g, 23.6 mmol, 1.5 eq) and HOBt hydrate (3.19 g, 23.6 mmol, 1.5 eq), cooled down to 0 °C under N_2 atmosphere, and supplemented with *N*,*N*-diisopropylethylamine (8.2 mL, 47.2 mmol, 3.0 eq) and EDC (4.52 g, 23.6 mmol, 1.5 eq). The mixture was then allowed to warm to room temperature and stir overnight, thereafter concentrated *in vacuo*, dissolved in EtOAc (200 mL) and washed with aqueous $KHSO_4$ (5% w/v, 200 mL) and saturated aqueous $NaHCO_3$ (200 mL). Both aqueous layers were extracted with EtOAc, and the combined organic layer was washed with brine (100 mL), dried over Na_2SO_4, filtered, and concentrated *in vacuo*. Column chromatography (35–100% EtOAc in heptane) afforded the title compound as off-white solid (4.36 g, 62% over 2 steps). ^{1}H NMR (600 MHz, CDCl$_3$): *δ* = 8.47 (s, 1H, CH$_2$CON$\underline{\mathrm{H}}$), 7.44 (d, *J* = 7.6 Hz, 1H, C3H or C6H), 7.41 (d, *J* = 7.6 Hz, 1H, C3H or C6H), 7.21 (s, 1H, OCONH), 7.14 (ddd, *J* = 7.6, 7.6, 1.3, 1H, C4H or C5H), 7.11 (ddd, *J* = 7.6, 7.6, 1.3 Hz, 1H, C4H or C5H), 6.48 (s, 1H, N$\underline{\mathrm{H}}$CH), 4.53 (q, *J* = 7.6 Hz, 1H, CH), 4.17 (q, *J* = 7.2 Hz, 2H, C$\underline{\mathrm{H}}_2$CH$_3$), 2.35 (td, *J* = 7.2, 2.7 Hz, 2H, CH$_2$CO), 2.00 (s, 3H, CH$_3$CO), 1.84–1.76 (m, 1H, CHC$\underline{\mathrm{H}}_{2,a}$), 1.76–1.61 (m, 3H, CHC$\underline{\mathrm{H}}_{2,b}$, C$\underline{\mathrm{H}}_2CH_2$CO), 1.50 (s, 9H, C(CH$_3$)$_3$), 1.44–1.29 (m, 4H, CHCH$_2$C$\underline{\mathrm{H}}_2$, C$\underline{\mathrm{H}}_2$CH$_2$CH$_2$CO), 1.26 (t, *J* = 7.2 Hz, 1H, CH$_2$C$\underline{\mathrm{H}}_3$). ^{13}C NMR (151 MHz, CDCl$_3$): *δ* = 172.7 (CH$\underline{\mathrm{C}}$O), 172.7 (CH$_2\underline{\mathrm{C}}$O), 172.4 (CH$_3\underline{\mathrm{C}}$O), 171.0 (OCON), 131.0 (C1 or C2), 130.1 (C1 or C2), 126.3 (C4 or C5), 125.6 (C3 or C6), 125.4 (C4 or C5), 124.7 (C3 or C6), 80.9 ($\underline{\mathrm{C}}$(CH$_3$)$_3$), 61.6 ($\underline{\mathrm{C}}$H$_2$CH$_3$), 52.5 (CH), 36.8 ($\underline{\mathrm{C}}$H$_2$CO), 32.2 (CH$\underline{\mathrm{C}}$H$_2$), 28.5 ($\underline{\mathrm{C}}$H$_2$CH$_2$CH$_2$CO), 28.4 (C($\underline{\mathrm{C}}$H$_3$)$_3$), 25.2 ($\underline{\mathrm{C}}$H$_2$CH$_2$CO), 24.9 (CHCH$_2\underline{\mathrm{C}}$H$_2$), 22.9 ($\underline{\mathrm{C}}$H$_3$CO), 14.3 CH$_2\underline{\mathrm{C}}$H$_3$). HRMS: *m*/*z* [M+H]$^+$ calcd for $C_{23}H_{36}N_3O_6^+$: 450.25986, found: 450.26094; [M+Na]$^+$ calcd for $C_{23}H_{35}N_3NaO_6^+$: 472.24181, found: 472.24292.

2-Acetamido-8-((2-((tert-butoxycarbonyl)amino)phenyl)amino)-8-oxooctanoic acid (**S7**). Compound **S6** (4.30 g, 9.56 mmol, 1.0 eq) was suspended in ethanol (40 mL) and water (25 mL), and stirred with aqueous LiOH (1.0 M, 15.3 mL, 15.3 mmol, 1.6 eq) at room temperature for 3 h. Then, the mixture was concentrated *in vacuo*, acidified to pH 2 with aq HCl (2 M) and extracted with EtOAc (3 × 150 mL). The aqueous layer was re-acidified after each extraction. The combined organic layer was washed with brine (150 mL), dried over Na_2SO_4, filtered, and concentrated *in vacuo* to the title compound as white solid (3.55 g, 88%). ^{1}H NMR (600 MHz, CD$_3$OD): *δ* = 7.51 (d, *J* = 7.8 Hz, 1H, C3H or C6H), 7.37 (dd, *J* = 7.8, 1.5 Hz, 1H, C3H or C6H), 7.20 (td, *J* = 7.8, 1.5 Hz, 1H, C4H or C5H), 7.13 (td, *J* = 7.8, 1.5 Hz, 1H, C4H or C5H), 4.37 (dd, *J* = 8.9, 4.9 Hz, 1H, CH), 2.43 (t, *J* = 7.4 Hz, 1H,

CH_2CO), 1.98 (s, 3H, CH_3CO), 1.92–1.82 (m, 1H, $CHC\underline{H}_{2,a}$), 1.78–1.66 (m, 3H, $CHC\underline{H}_{2,b}$, $C\underline{H}_2CH_2CO$), 1.51 (s, 9H, $C(CH_3)_3$), 1.51–1.41 (m, 4H, $CHCH_2C\underline{H}_2$, $C\underline{H}_2CH_2CH_2CO$). ^{13}C NMR (151 MHz, CD_3OD): δ = 175.6 (COOH), 175.1 ($CH_2\underline{C}O$), 173.3 ($CH_3\underline{C}O$), 155.8 (OCON), 133.1 (C1 or C2), 131.1 (C1 or C2), 127.3 (C4 or C5), 126.6 (C3 or C6), 125.9 (C4 or C5), 125.5 (C3 or C6), 81.5 ($\underline{C}(CH_3)_3$), 53.7 (CH), 37.4 ($\underline{C}H_2CO$), 32.6 ($CH\underline{C}H_2$), 29.8 ($\underline{C}H_2CH_2CH_2CO$), 28.7 ($C(\underline{C}H_3)_3$), 26.7 ($CHCH_2\underline{C}H_2$, $\underline{C}H_2CH_2CO$), 22.3 ($\underline{C}H_3CO$). HRMS: *m*/*z* $[M+H]^+$ calcd for $C_{21}H_{32}N_3O_6^+$: 422.22856, found: 422.22953; $[M+Na]^+$, calcd for $C_{21}H_{31}N_3NaO_6^+$: 444.21051, found: 444.21150.

(S)-2-((((9H-Fluoren-9-yl)methoxy)carbonyl)amino)-8-((2-((tert-butoxycarbonyl)amino)phenyl)amino)-8-oxooctanoic acid (**S8**). Compound **S7** (3.55 g, 8.42 mmol, 1.0 eq) was suspended in phosphate buffer (0.1 M, pH 7.6, 200 mL), pH was adjusted to 7.2 with aqueous NaOH (2.0 M), and the mixture was warmed to 40 °C. Acylase I from *Aspergillus Melleus* (202 mg, >0.5 U/mg) and $CoCl_2 \cdot 6H_2O$ (48 mg, to reach 10^{-3} M) were added, and the reaction was stirred at 40 °C overnight. Deacetylation was followed both by LCMS with UV detection (258 nm), and by NMR. For the latter, a control NMR sample was taken prior to addition of the enzyme and at selected reaction time points (50 µL reaction mixture in 450 µL CD_3OD). ^1H NMR experiments with water suppression were used to measure the relative intensity of the $NHC\underline{H}$ signal, and the reaction was judged complete upon reaching 50% of the initial intensity. A second addition of Acylase I (302 mg) and $CoCl_2 \cdot 6H_2O$ (48 mg) at 24 h reaction time and overnight stirring were necessary in order to reach completion. Then, the pH of the mixture was increased to 9.0 with aq NaOH (2.0 M), and a solution of Fmoc *N*-hydroxysuccinimide ester (2.13 g, 6.31 mmol, 0.8 eq) in 1,4-dioxane (20 mL) was added. The mixture was stirred overnight at room temperature, and then it was concentrated *in vacuo*, acidified to pH 2.0 with aq HCl (2.0 M) and extracted with EtOAc (200 mL + 100 mL + 70 mL + 50 mL). The combined organic layer was washed with brine (150 mL), dried over Na_2SO_4, filtered, and concentrated *in vacuo*. Column chromatography (50–80% EtOAc in heptane, with 0.25% acetic acid) afforded the title compound as off-white solid (1.24 g, 49% relative to the desired enantiomer). ^1H NMR (600 MHz, DMSO-d_6): δ = 12.53 (s, 1H, COOH), 9.43 (s, 1H, $CH_2CON\underline{H}$), 8.30 (s, 1H, OCONH), 7.89 (d, J = 7.5 Hz, 2H, $C4_{Fmoc}H$, $C5_{Fmoc}H$), 7.73 (dd, J = 7.4, 3.3 Hz, 2H, $C1_{Fmoc}H$, $C8_{Fmoc}H$), 7.63 (d, J = 8.1 Hz, 1H, $N\underline{H}CH$), 7.53 (d, J = 7.9 Hz, 1H, $C3_{Ph}H$), 7.41 (m, 3H, $C6_{Ph}H$, $C3_{Fmoc}H$, $C6_{Fmoc}H$), 7.33 (t, J = 7.4 Hz, 2H, $C2_{Fmoc}H$, $C7_{Fmoc}H$), 7.15–7.11 (m, 1H, $C4_{Ph}H$), 7.07 (td, J = 7.8, 1.4 Hz, 1H, $C5_{Ph}H$), 4.28 (d, J = 7.5 Hz, 2H, $CHC\underline{H}_2O$), 4.24–4.20 (m, 1H, $C\underline{H}CH_2O$), 3.94 (td, J = 9.1, 4.8 Hz, 1H, $NHC\underline{H}$), 2.34 (t, J = 7.2 Hz, 2H, CH_2CO), 1.76–1.67 (m, 1H, $NHCHC\underline{H}_{2,a}$), 1.65–1.58 (m, 3H, $NHCHC\underline{H}_{2,b}$, $C\underline{H}_2CH_2CO$), 1.44 (s, 9H, $C(CH_3)_3$), 1.40–1.27 (m, 4H, $CHCH_2C\underline{H}_2$, $C\underline{H}_2CH_2CH_2CO$). ^{13}C NMR (151 MHz, DMSO): δ = 173.9 (COOH), 171.7 ($CH_2\underline{C}O$), 156.1 ($\underline{C}ONHCH$), 153.0 ($\underline{C}OOC(CH_3)_3$), 143.8 ($C8a_{Fmoc}$ or $C9a_{Fmoc}$), 143.8 ($C8a_{Fmoc}$ or $C9a_{Fmoc}$), 140.7 ($C4a_{Fmoc}$ or $C4b_{Fmoc}$), 140.7 ($C4a_{Fmoc}$ or $C4b_{Fmoc}$), 131.1 ($C2_{Ph}$), 129.6 ($C1_{Ph}$), 127.6 ($C3_{Fmoc}$, $C6_{Fmoc}$), 127.0 ($C2_{Fmoc}$, $C7_{Fmoc}$), 125.3 ($C1_{Fmoc}$, $C8_{Fmoc}$), 125.0 ($C4_{Ph}$), 124.8 ($C6_{Ph}$), 123.8 ($C5_{Ph}$), 123.6 ($C3_{Ph}$), 120.1 ($C4_{Fmoc}$, $C5_{Fmoc}$), 79.3 ($\underline{C}(CH_3)_3$), 65.6 ($CH\underline{C}H_2O$), 53.8 (NHCH), 46.7 ($\underline{C}HCH_2O$), 35.9 ($\underline{C}H_2CO$), 30.7 ($NHCH\underline{C}H_2$), 28.1 ($\underline{C}H_2CH_2CH_2CO$), 28.0 ($C(\underline{C}H_3)_3$), 25.4 ($NHCHCH_2\underline{C}H_2$), 25.1 ($\underline{C}H_2CH_2CO$). HRMS: *m*/*z* $[M+H]^+$ calcd for $C_{34}H_{40}N_3O_7^+$: 602.28608, found: 602.28710; $[M+Na]^+$ calcd for $C_{34}H_{39}N_3NaO_7^+$: 624.26802, found: 624.26893.

Peptide Synthesis. Standard Fmoc/^{t}Bu SPPS of Asuha- and acetyl-lysine-containing peptides for validation was performed on a Biotage SyroWave™ automated synthesizer with protected amino acids

as before. The synthesis was executed on 20 or 40 µmol scale using pre-loaded TentaGel S RAM Resin (0.24 mmol/g, Rapp Polymere). Fmoc deprotection steps were performed twice with piperidine/DMF (first: 2:3 (v/v), 3 min; then: 1:4 (v/v), 12 min), followed by washing of the resin with DMF (2 × 45 s), CH_2Cl_2 (45 s) and DMF (2 × 45 s). Coupling reactions were performed with Fmoc-Xaa-OH (200 µL; 0.4 M in DMF with 0.4 M HOAt, 4.0 equiv; or 0.5 M in DMF, 5.0 equiv), HBTU (210 µL; 0.39 M in DMF, 4.0 equiv; or 0.48 M in DMF, 5.0 equiv), and $^{i}Pr_2NEt$ (100 µL; 1.6 M in NMP, 8.0 equiv; or 2.0 M in NMP, 10.0 equiv) for 40 min, followed by washing with DMF (3 × 45 s). Final capping was performed with acetic acid in a similar manner. Peptides were cleaved under TFA–DODT–H_2O–$^{i}Pr_3SiH$, [2 mL, 92.5:2.5:2.5:2.5 (v/v)] for 2 h at room temperature, followed by concentration under N_2 stream, trituration with ice cold diethyl ether (7 mL) and centrifugation for at 1600 rcf (3 min, room temperature). Supernatants were discarded, and pellets were washed with ice cold diethyl ether (2 × 7 mL), dissolved in H_2O/CH_3CN [95:5 with 0.1% TFA (v/v)] and lyophilized. Asuha-containing peptides were treated for additional 2 h at room temperature with TFA–CF_3SO_3H–H_2O–$^{i}Pr_3SiH$ [1.5 mL, 88.5:4:2.5:5 (v/v)] to achieve deprotection of the Asuha side chain, followed by trituration, centrifugation, washing and lyophilization as before. Peptide purification was achieved by preparative HPLC as described (see Supplementary Fig. 13 for purity traces).

H4(5–19)K12Asuha (**1a**): t_R = 4.62 min (UPLC), 99% purity.
HRMS: *m*/*z* $[M + H]^+$ calcd for $C_{78}H_{131}N_{30}O_{19}^+$: 1792.0201, found: 1792.0221.

H4(1–15)K12Asuha (**2a**): t_R = 4.99 min (UPLC), 97% purity.
HRMS: *m*/*z* $[M + H]^+$ calcd for $C_{67}H_{111}N_{24}O_{20}^+$: 1571.8401, found: 1571.8421.

H4(5–14)K12Asuha (**3a**): t_R = 5.04 min (UPLC), 98% purity.
HRMS: *m*/*z* $[M + H]^+$ calcd for $C_{51}H_{83}N_{16}O_{14}^+$: 1143.6269, found: 1143.6257.

H4(9–23)K12Asuha (**4a**): t_R = 5.84 min (UPLC), 98% purity.
HRMS: *m*/*z* $[M + H]^+$ calcd for $C_{85}H_{145}N_{32}O_{19}^+$: 1918.1358, found: 1918.1397.

H4(10–19)K12Asuha (**5a**): t_R = 5.15 min (UPLC), 97% purity.
HRMS: *m*/*z* $[M + H]^+$ calcd for $C_{60}H_{98}N_{23}O_{14}^+$: 1364.7658, found: 1364.7673.

H3(1–16)K9Asuha (**6a**): t_R = 9.9 min (C8 column), 99% purity.
HRMS: *m*/*z* $[M + 3H]^{3+}$ calcd for $C_{83}H_{142}N_{29}O_{25}^{3+}$: 648.69163; found: 648.69084; $[M + 4H]^{4+}$ calcd for $C_{83}H_{143}N_{29}O_{25}^{4+}$: 486.77054; found: 486.76988.

H3(1–16) (**6b**): t_R = 9.3 min, 97% purity.
HRMS: *m*/*z* $[M + 2H]^{2+}$ calcd for $C_{81}H_{139}N_{29}O_{23}^{2+}$: 943.02938; found: 943.02885; $[M + 3H]^{3+}$ calcd for $C_{81}H_{140}N_{29}O_{23}^{3+}$: 629.02201; found: 629.02161.

H3(1–16)K9ac (**6c**): t_R = 9.9 min, 97% purity.
HRMS: *m*/*z* $[M + 3H]^{3+}$ calcd for $C_{83}H_{142}N_{29}O_{24}^{3+}$: 643.02554; found: 643.02456; $[M + 4H]^{4+}$ calcd for $C_{83}H_{143}N_{29}O_{24}^{4+}$: 482.52097; found: 482.52039.

H3(1–16)K4thioac, K9Asuha (**7a**): t_R = 11.4 min (C8 column), 98% purity.

HRMS: m/z $[M + 2H]^{2+}$ calcd for $C_{85}H_{143}N_{29}O_{25}S^{2+}$: 1001.02598; found: 1001.02892; $[M + 3H]^{3+}$ calcd for $C_{85}H_{144}N_{29}O_{25}S^{3+}$: 667.68641; found: 667.68670.

H3(1–16)K4thioac (**7b**): t_R = 11.0 min, 97% purity.
HRMS: m/z $[M + 3H]^{3+}$ calcd for $C_{83}H_{142}N_{29}O_{23}S^{3+}$: 648.35125; found: 648.35001; $[M + 4H]^{4+}$ calcd for $C_{83}H_{143}N_{29}O_{23}S^{4+}$: 486.51526; found: 486.51448.

H3(1–16)K4thioac, K9ac (**7c**): t_R = 11.8 min, 98% purity.
HRMS: m/z $[M + 2H]^{2+}$ calcd for $C_{85}H_{143}N_{29}O_{24}S^{2+}$: 993.02853; found: 993.02707; $[M + 3H]^{3+}$ calcd for $C_{85}H_{144}N_{29}O_{24}S^{3+}$: 662.35478; found: 662.35397.

H3(1–16)K4thioac, K9Asuha, pS10 (**8a**): t_R = 10.8 min, 97% purity.
MALDI: m/z $[M + H]^{+}$ calcd for $C_{85}H_{143}N_{29}O_{28}PS^{+}$: 2081.01102; found: 2081.06300.

H3(1–16)K4thioac, pS10 (**8b**): t_R = 10.6 min, 97% purity.
HRMS: m/z $[M + 2H]^{2+}$ calcd for $C_{83}H_{142}N_{29}O_{26}PS^{2+}$: 1012.00641; found: 1012.00483; $[M + 3H]^{3+}$ calcd for $C_{83}H_{143}N_{29}O_{26}PS^{3+}$: 675.00670; found: 675.00586. MALDI: m/z $[M + H]^{+}$ calcd for $C_{83}H_{141}N_{29}O_{26}PS^{+}$: 2023.00554; found: 2023.28400.

H3(1–16)K4thioac, K9ac, pS10 (**8c**): t_R = 11.2 min, 97% purity.
HRMS: m/z $[M + 2H]^{2+}$ calcd for $C_{85}H_{144}N_{29}O_{27}PS^{2+}$: 1033.01169; found: 1033.01063; $[M + 3H]^{3+}$ calcd for $C_{85}H_{145}N_{29}O_{27}PS^{3+}$: 689.01022; found: 689.00964. MALDI: m/z $[M + H]^{+}$ calcd for $C_{85}H_{143}N_{29}O_{27}PS^{+}$: 2065.01610; found: 2064.93100.

H2A(7–22)K9Asuha (**9a**): t_R = 12.6 min, 95% purity.
HRMS: m/z $[M + 2H]^{2+}$ calcd for $C_{79}H_{137}N_{31}O_{23}{}^{2+}$: 944.02463, found: 944.02332; $[M + 3H]^{3+}$ calcd for $C_{79}H_{138}N_{31}O_{23}{}^{3+}$: 629.68551, found: 629.68481.

H2A(7–22) (**9b**): t_R = 9.4 min (C8 column), 96% purity.
HRMS: m/z $[M + 3H]^{3+}$ calcd for $C_{77}H_{136}N_{31}O_{21}{}^{3+}$: 610.35035; found: 610.34950; $[M + 4H]^{4+}$ calcd for $C_{77}H_{137}N_{31}O_{21}{}^{4+}$: 458.01458; found: 458.01400.

H2A(7–22)K9ac (**9c**): t_R = 12.7 min, 95% purity.
HRMS: m/z $[M + 3H]^{3+}$ calcd for $C_{79}H_{138}N_{31}O_{22}{}^{3+}$: 624.35387, found: 624.35275; $[M + 4H]^{4+}$ calcd for $C_{79}H_{139}N_{31}O_{22}{}^{4+}$: 468.51723, found: 468.51649.

H2B(7–22)K12Asuha (**10a**): t_R = 13.7 min, 97% purity.
HRMS: m/z $[M + 2H]^{2+}$ calcd for $C_{86}H_{145}N_{25}O_{23}{}^{2+}$: 948.04671, found: 948.04500; $[M + 3H]^{3+}$ calcd for $C_{86}H_{146}N_{25}O_{23}{}^{3+}$: 632.36690, found: 632.36596.

H2B(7–22) (**10b**): t_R = 10.1 min (C8 column), 99% purity.
HRMS: m/z $[M + 3H]^{3+}$ calcd for $C_{84}H_{144}N_{25}O_{21}{}^{3+}$: 613.03174; found: 613.03067; found:; $[M + 4H]^{4+}$ calcd for $C_{84}H_{145}N_{25}O_{21}{}^{4+}$: 460.02562; found: 460.02492.

H2B(7–22)K12ac (**10c**): t_R = 13.0 min, 99% purity.
HRMS: m/z $[M + 2H]^{2+}$ calcd for $C_{86}H_{145}N_{25}O_{22}{}^{2+}$: 940.04925, found: 940.04856; $[M + 3H]^{3+}$ calcd for $C_{86}H_{146}N_{25}O_{22}{}^{3+}$: 627.03526, found: 627.03470.

H2B(76–91)K85Asuha (**11a**): t_R = 6.17 min (UPLC), 99% purity.

HRMS: *m*/*z* [M + H]$^+$ calcd for $C_{92}H_{144}N_{29}O_{29}^+$: 2119.0679, found: 2119.0686.

H2B(76–91) (**11b**): t_R = 5.84 min (UPLC), 96% purity.
HRMS: *m*/*z* [M + H]$^+$ calcd for $C_{90}H_{142}N_{29}O_{27}^+$: 2061.0624, found: 2061.0664.

H2B(82–97)K85Asuha (**12a**): t_R = 6.03 min (UPLC), 96% purity.
HRMS: *m*/*z* [M + H]$^+$ calcd for $C_{95}H_{149}N_{30}O_{30}^+$: 2190.1050, found: 2190.1042.

H2B(82–97) (**12b**): t_R = 5.92 min (UPLC), 92% purity.
HRMS: *m*/*z* [M + H]$^+$ calcd for $C_{93}H_{147}N_{30}O_{28}^+$: 2132.0996, found: 2132.1018.

H3(7–22)K18Asuha (**13a**): t_R = 5.16 min (UPLC), 98% purity.
HRMS: *m*/*z* [M + H]$^+$ calcd for $C_{85}H_{144}N_{29}O_{24}^+$: 1955.0934, found: 1955.0961.

H3(7–22) (**13b**): t_R = 5.20 min (UPLC), 99% purity.
HRMS: *m*/*z* [M + H]$^+$ calcd for $C_{83}H_{142}N_{29}O_{22}^+$: 1897.0879, found: 1897.0909.

H3(16–31)K27Asuha (**14a**): t_R = 14.2 min, 97% purity.
HRMS: *m*/*z* [M + 3H]$^{3+}$ calcd for $C_{88}H_{150}N_{29}O_{23}^{3+}$: 660.38143, found: 660.38113; [M + 4H]$^{4+}$ calcd for $C_{88}H_{151}N_{29}O_{23}^{4+}$: 495.53789, found: 495.53765.

H3(16–31) (**14b**): t_R = 10.8 min (C8 column), 99% purity.
HRMS: *m*/*z* [M + 3H]$^{3+}$ calcd for $C_{86}H_{148}N_{29}O_{21}^{3+}$: 641.04627; found: 641.04500; [M + 4H]$^{4+}$ calcd for $C_{86}H_{149}N_{29}O_{21}^{4+}$: 481.03652; found: 481.28658.

H3(16–31)K27ac (**14c**): t_R = 14.7 min, 97% purity.
HRMS: *m*/*z* [M + 2H]$^{2+}$ calcd for $C_{88}H_{149}N_{29}O_{22}^{2+}$: 982.07105, found: 982.57157; [M + 3H]$^{3+}$ calcd for $C_{88}H_{150}N_{29}O_{22}^{3+}$: 655.04979, found: 655.04894.

H3(31-46)K36Asuha (**15a**): t_R = 15.4 min, 96% purity.
HRMS: *m*/*z* [M + H]$^+$ calcd for $C_{91}H_{142}N_{29}O_{23}^+$: 2009.08279, found: 2009.08114.

H3(31-46) (**15b**): t_R = 13.1 min (C8 column), 98% purity.
HRMS: *m*/*z* [M + 2H]$^{2+}$ calcd for $C_{89}H_{141}N_{29}O_{21}^{2+}$: 976.04229; found: 976.04183; [M + 3H]$^{3+}$ calcd for $C_{89}H_{142}N_{29}O_{21}^{3+}$: 651.03062; found: 651.03059.

H3(31-46)K36ac (**15c**): t_R = 14.7 min, 99% purity.
HRMS: *m*/*z* [M + H]$^+$ calcd for $C_{91}H_{142}N_{29}O_{22}^+$: 1993.08787, found: 1993.08501.

H4(10–25)K16Asuha (**16a**): t_R = 14.2 min, 99% purity.
HRMS: *m*/*z* [M + 2H]$^{2+}$ calcd for $C_{91}H_{154}N_{34}O_{23}^{2+}$: 1045.59576, found: 1046.09820; [M + 4H]$^{4+}$ calcd for $C_{91}H_{154}N_{34}O_{23}^{3+}$: 697.39960, found: 697.73397.

H4(10–25) (**16b**): t_R = 10.8 min (C8 column), 99% purity.
HRMS: *m*/*z* [M + 2H]$^{2+}$ calcd for $C_{89}H_{152}N_{34}O_{21}^{2+}$: 1016.59302; found: 1016.59366; [M + 3H]$^{3+}$ calcd for $C_{89}H_{153}N_{34}O_{21}^{3+}$: 678.06444; found: 678.39864.

H4(10–25)K16ac (**16c**): t_R = 13.8 min, 99% purity.
HRMS: *m*/*z* [M + 3H]$^{3+}$ calcd for $C_{91}H_{155}N_{34}O_{22}^{3+}$: 692.06796, found: 692.06707; [M + 4H]$^{4+}$ calcd for $C_{91}H_{156}N_{34}O_{22}^{4+}$: 519.30279, found: 519.30220.

H4(64–79)K77Asuha (**17a**): t_R = 7.94 min (UPLC), 98% purity.
HRMS: *m*/*z* $[M + H]^+$ calcd for $C_{97}H_{153}N_{30}O_{28}^+$: 2186.1465, found: 2186.1458.

H4(64–79) (**17b**): t_R = 7.25 min (UPLC), 97% purity.
HRMS: *m*/*z* $[M + H]^+$ calcd for $C_{95}H_{151}N_{30}O_{26}^+$: 2128.1410, found: 2128.1408.

Compound Stock Solution Preparation. Peptide inhibitor and peptide substrate DMSO stock solutions were prepared from the lyophilized powder after preparative HPLC. Concentrations were determined based on absorbance measured on a Thermo Scientific NanoDrop^{C} instrument using the appropriate fluorophore extinction coefficient [ε_{280}(Trp) = 5690 $M^{-1}cm^{-1}$, ε_{326}(Ac-Lys-AMC) = 17783 $M^{-1}cm^{-1}$ [3], $\varepsilon_{260,calc.}$(Trp+thioacetamide) = 15323 $M^{-1}cm^{-1}$] [4,5].

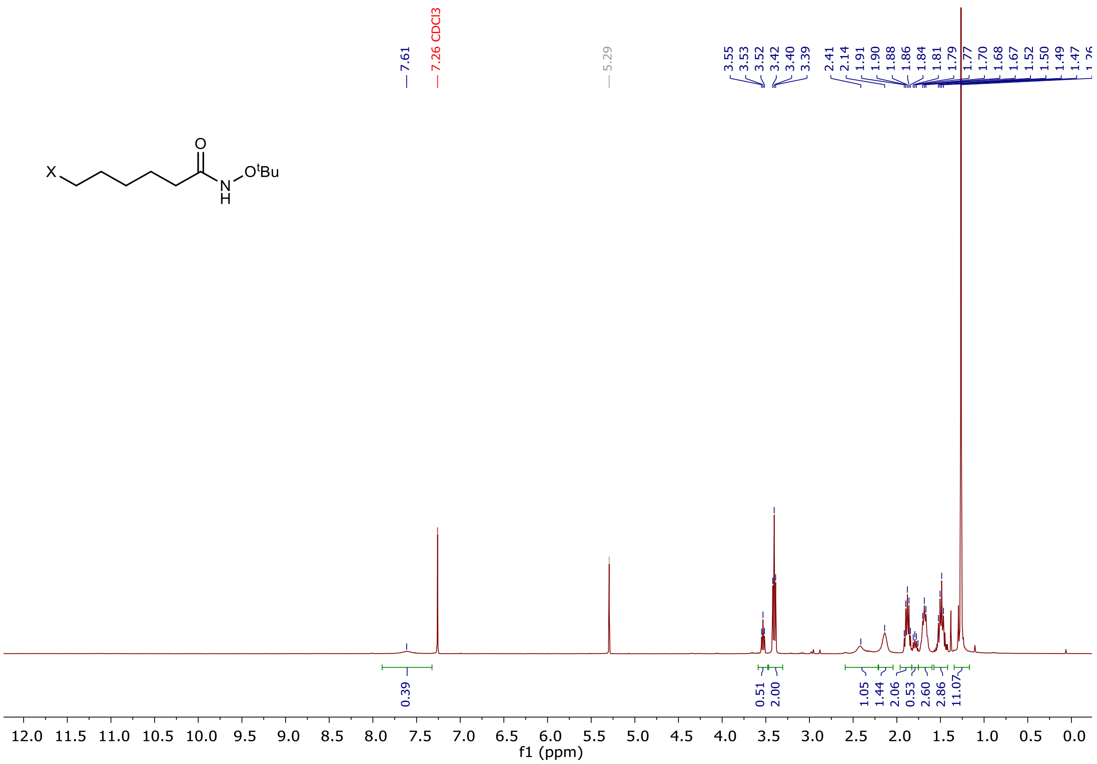

^1H NMR of 6-bromo/6-chloro-*N*-(*tert*-butoxy)hexanamide.

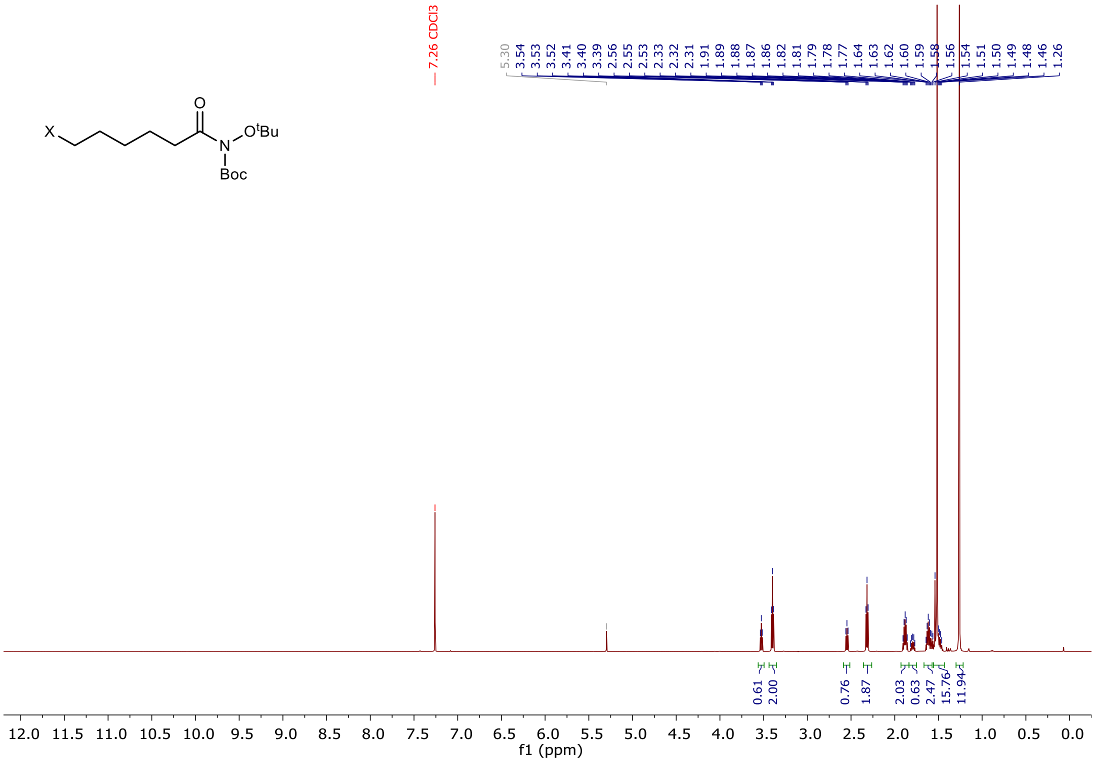

^{1}H NMR of **S1**/6-chloro-*N*-(*tert*-butoxy)-*N*-(*tert*-butoxycarbonyl)hexanamide.

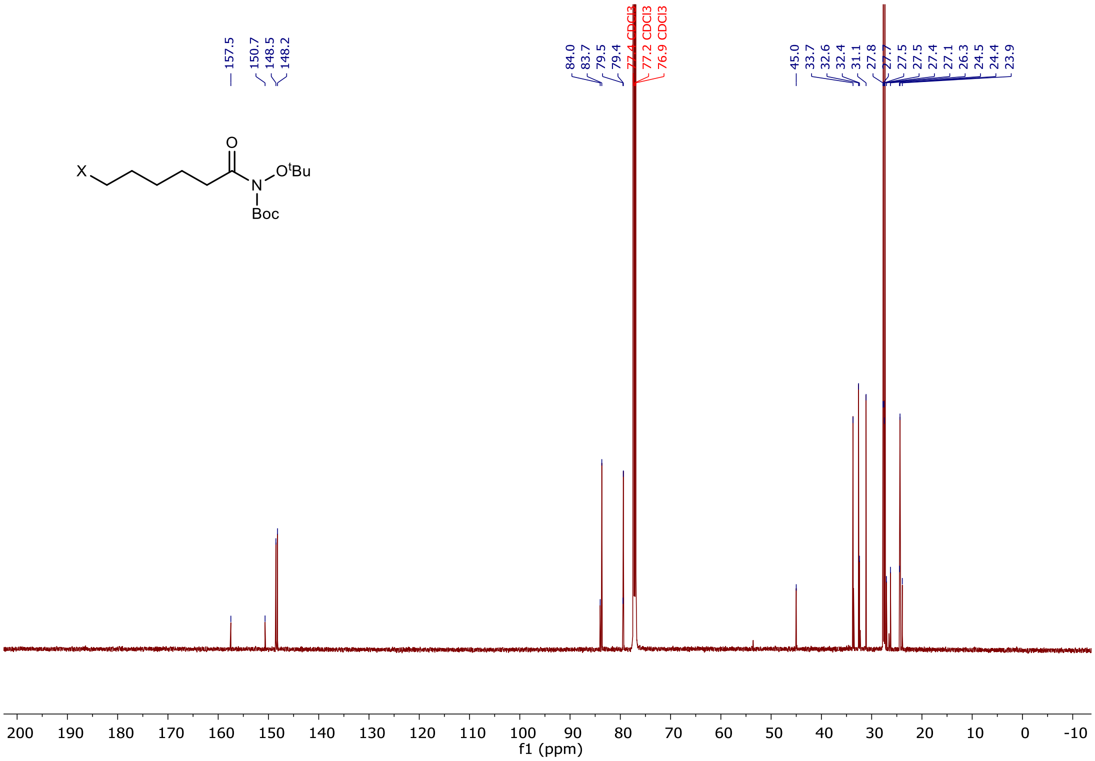

^{13}C NMR of **S1**/6-chloro-*N*-(*tert*-butoxy)-*N*-(*tert*-butoxycarbonyl)hexanamide.

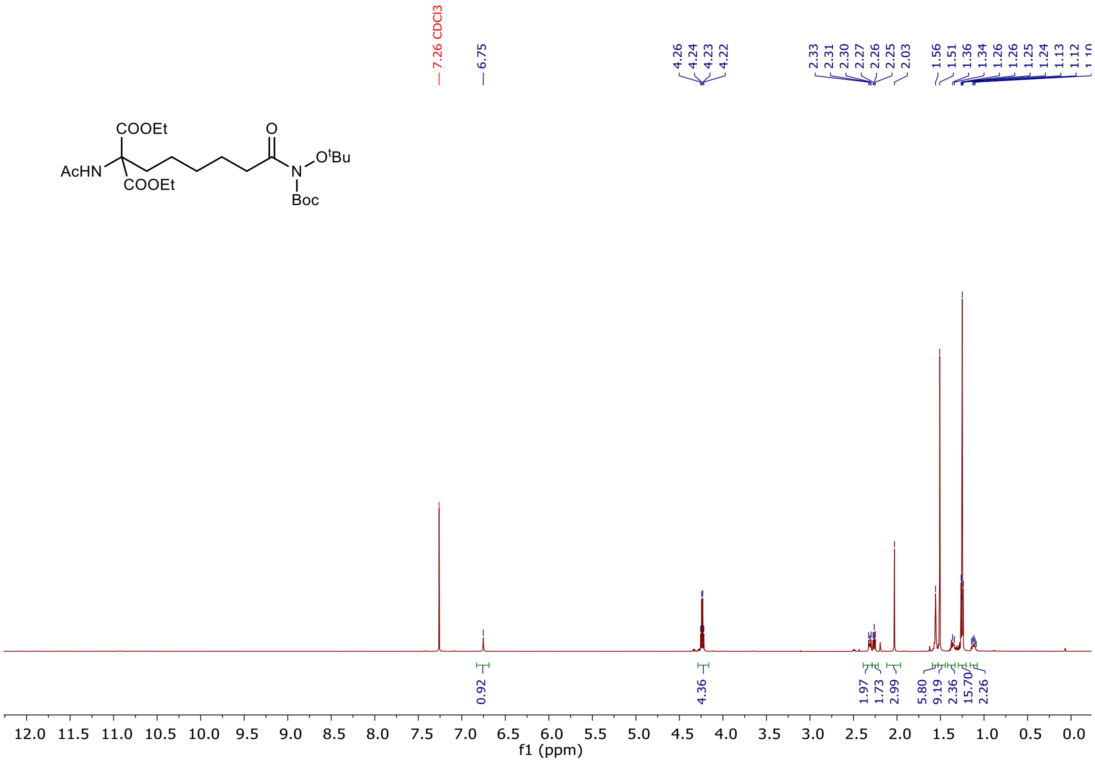

^{1}H NMR of compound **S3**.

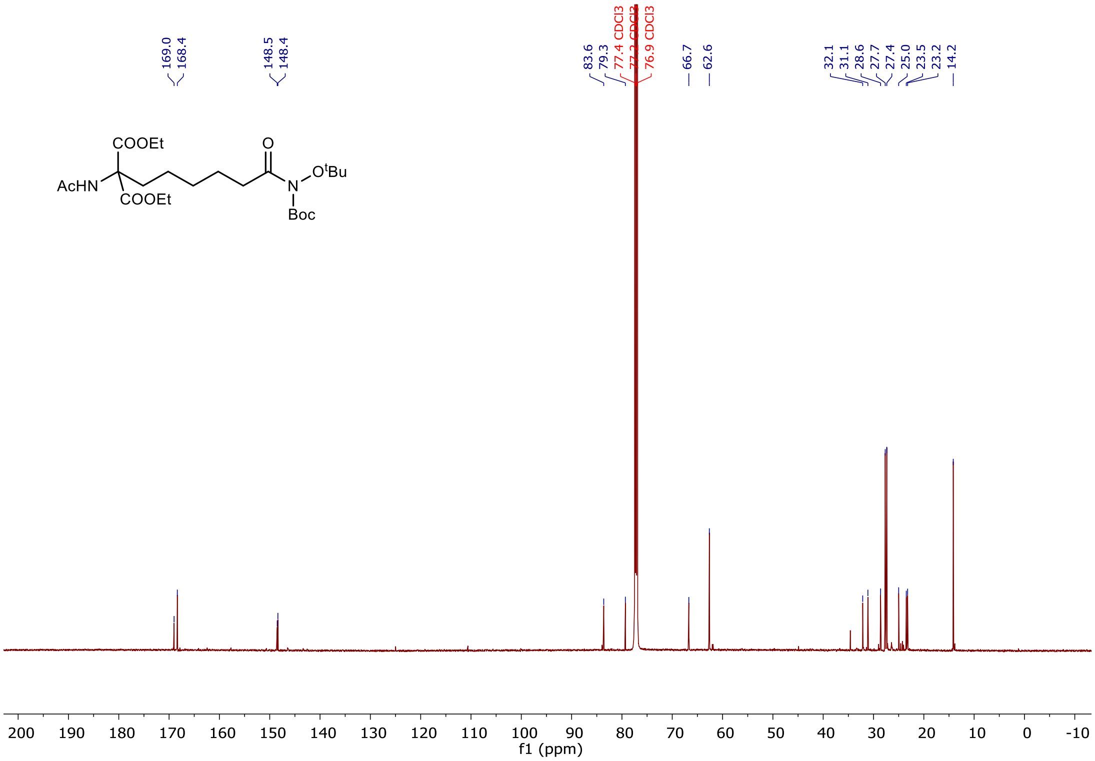

^{13}C NMR of compound **S3**.

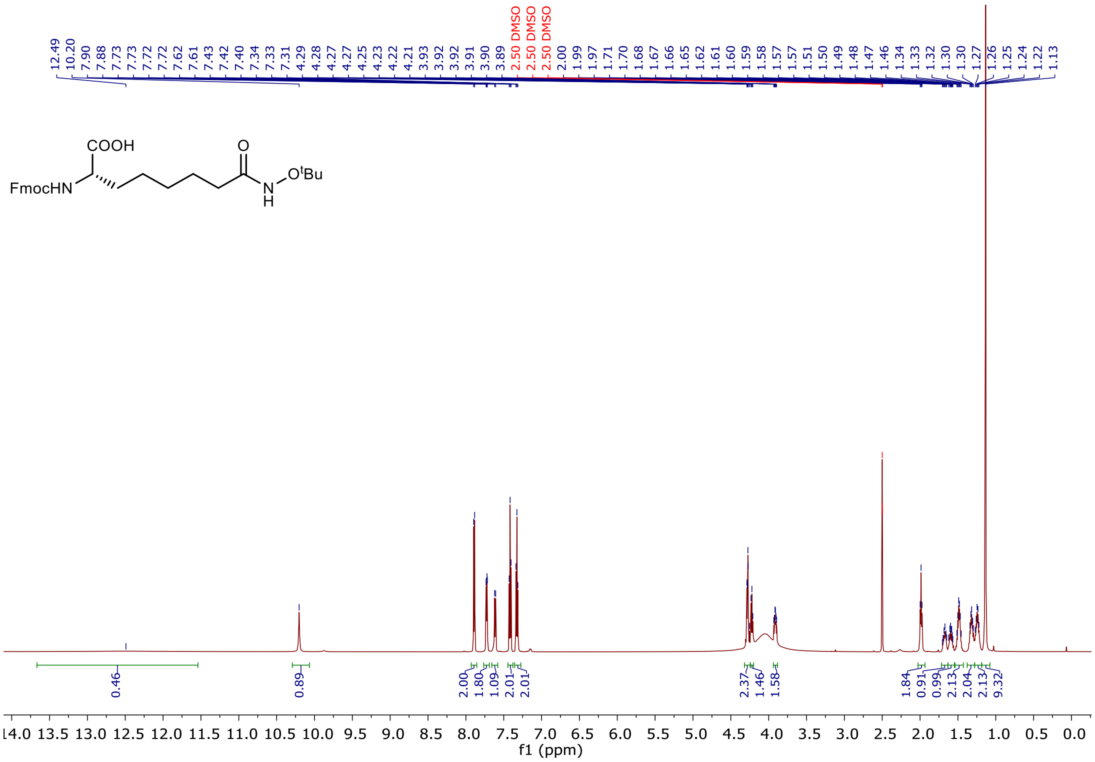

^{1}H NMR of compound **S5**.

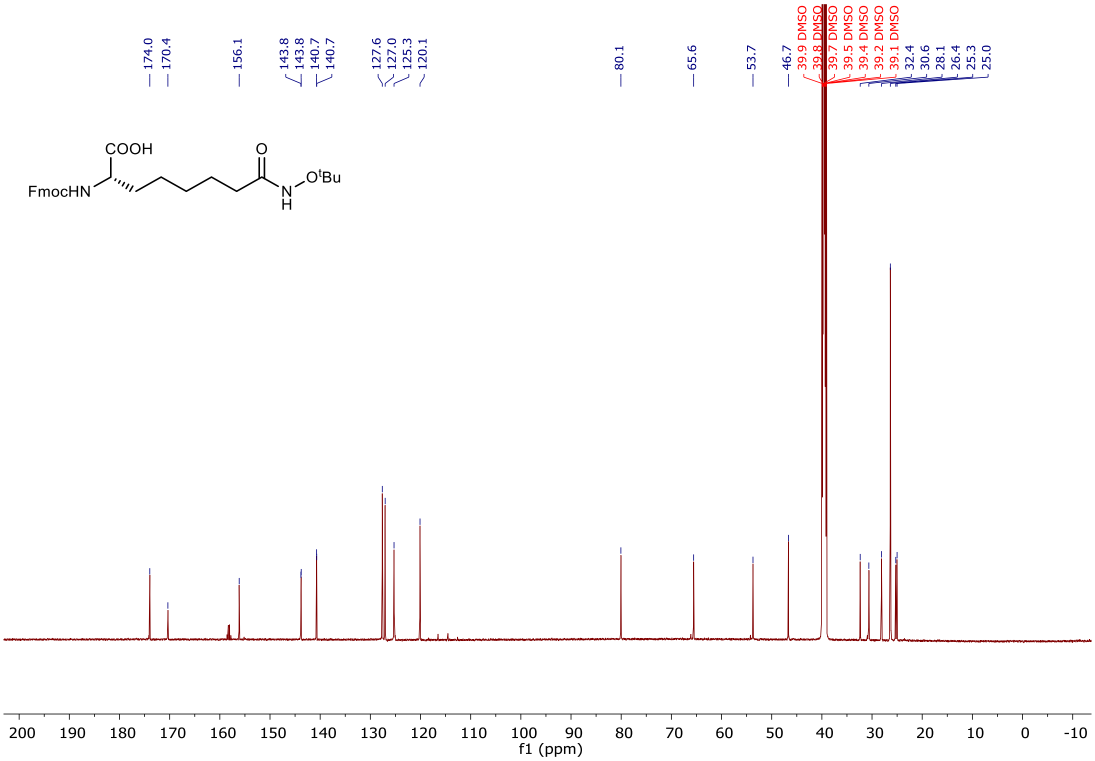

^{13}C NMR of compound **S5**.

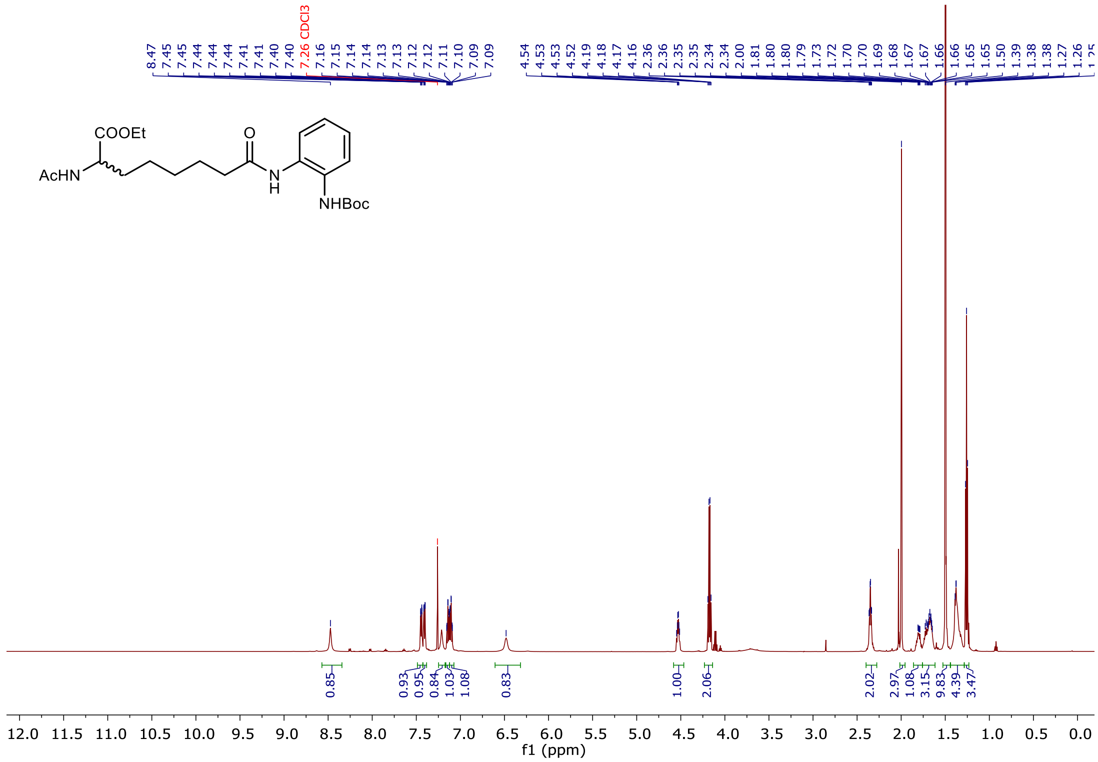

^{1}H NMR of compound **S6**.

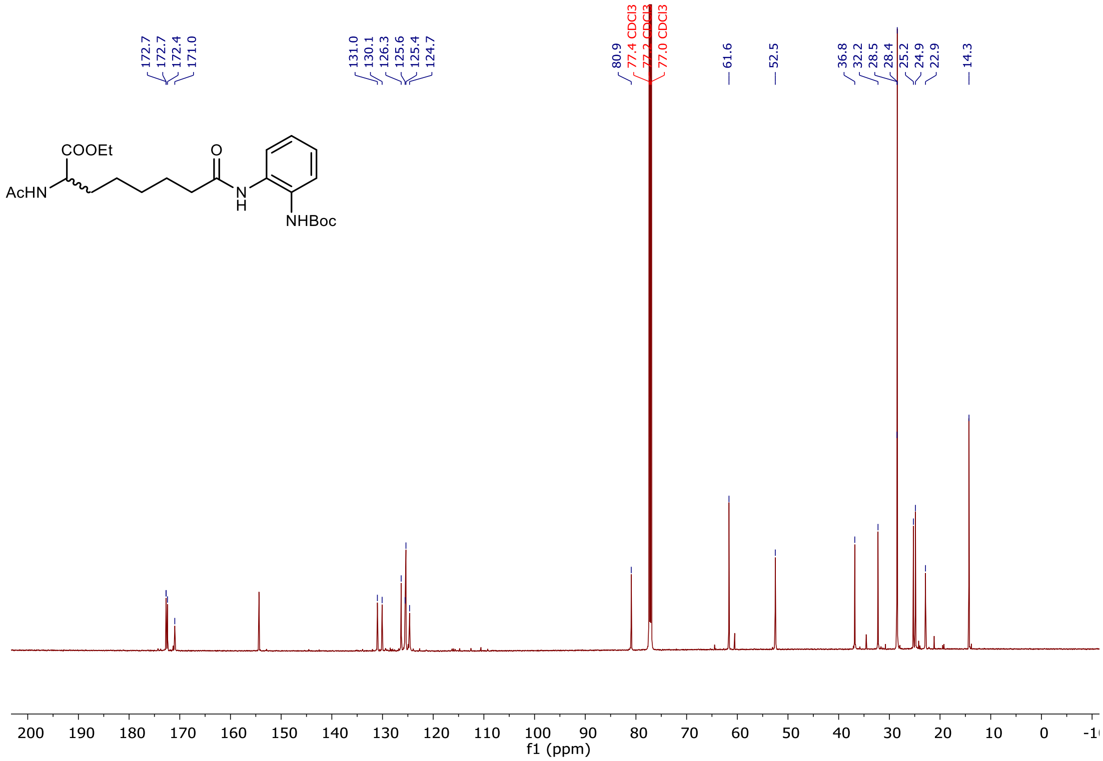

^{13}C NMR of compound **S6**.

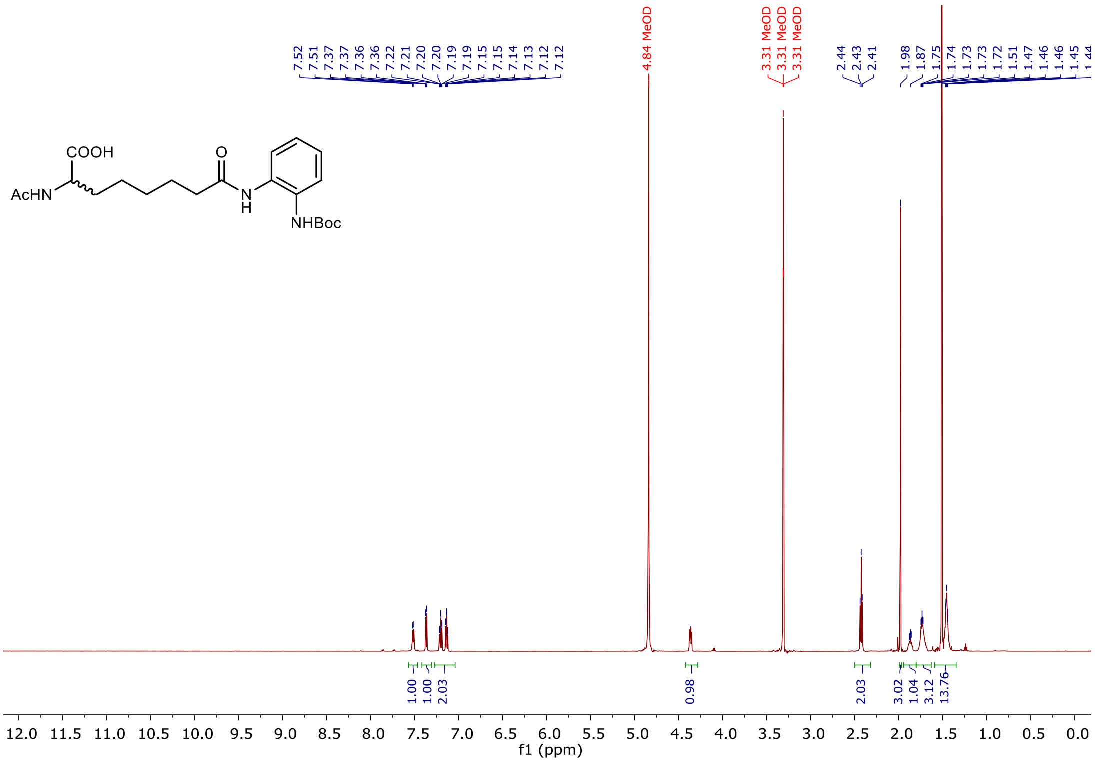

^{1}H NMR of compound **S7**.

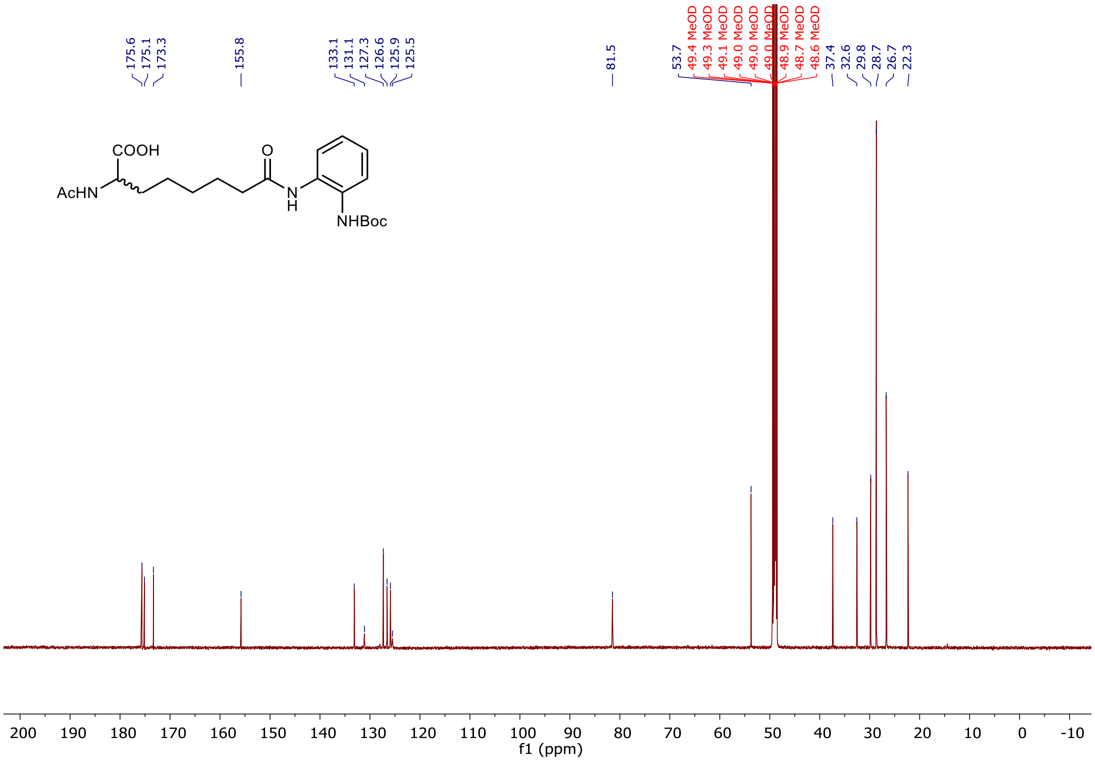

^{3}C NMR of compound **S7**.

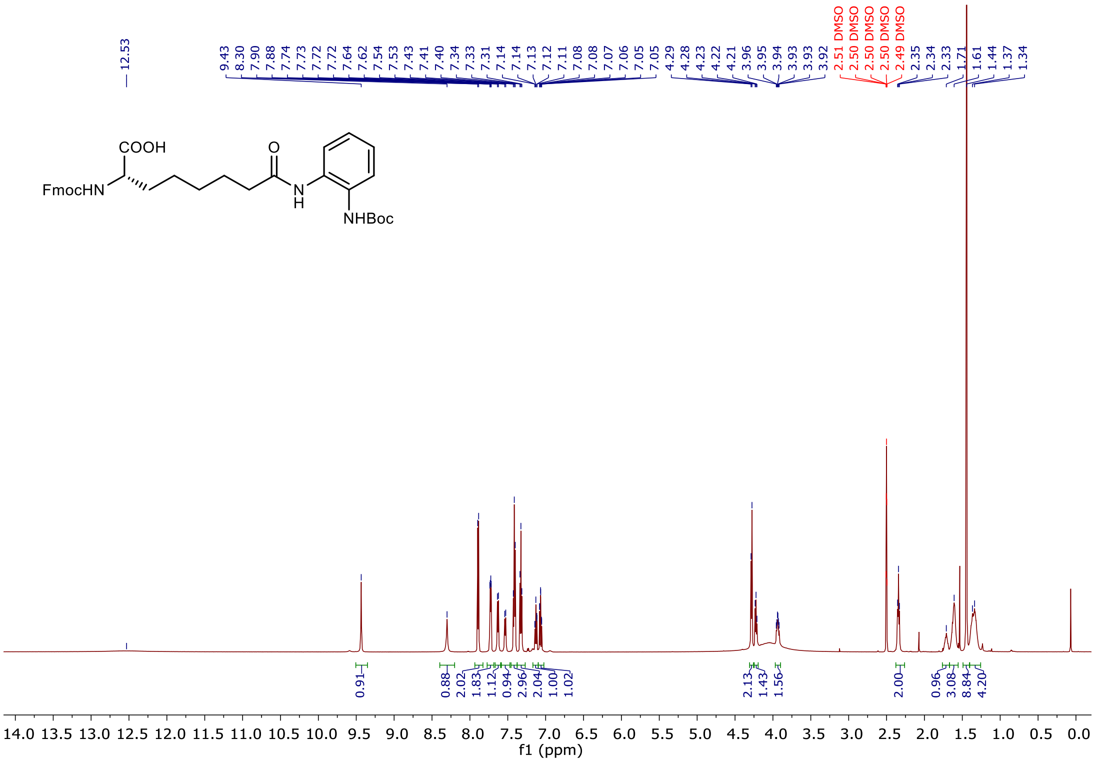

^{1}H NMR of compound **S8**.

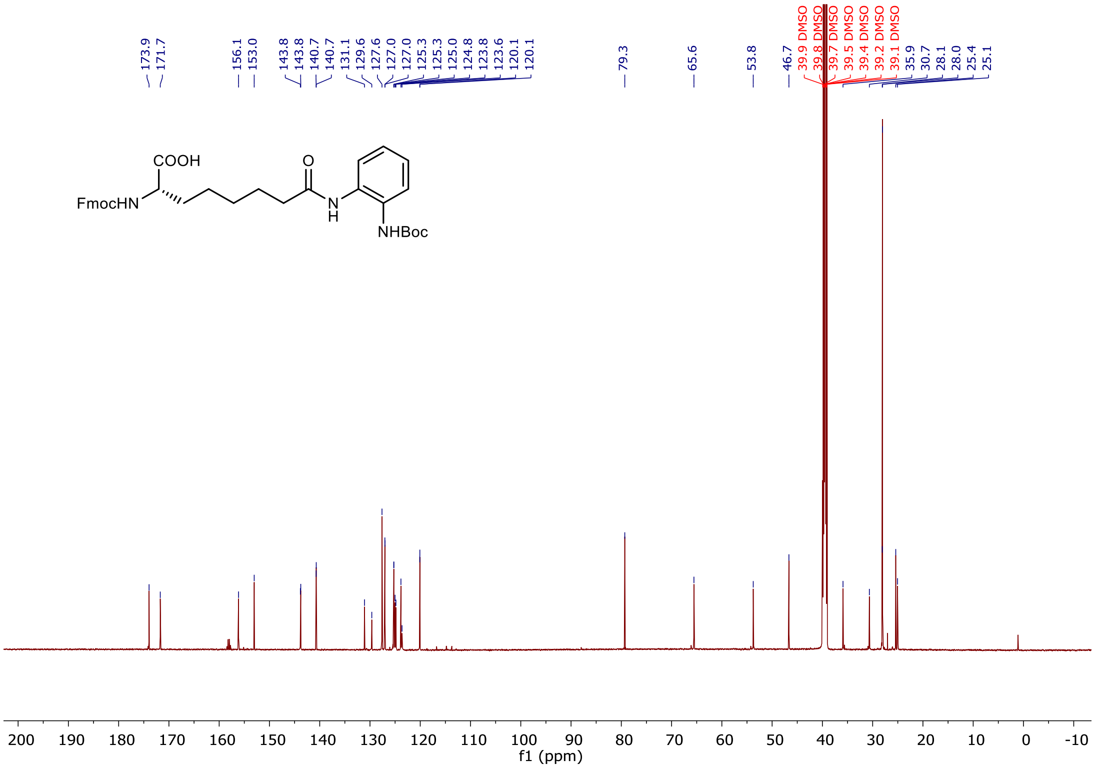

^{13}C NMR of compound **S8**.

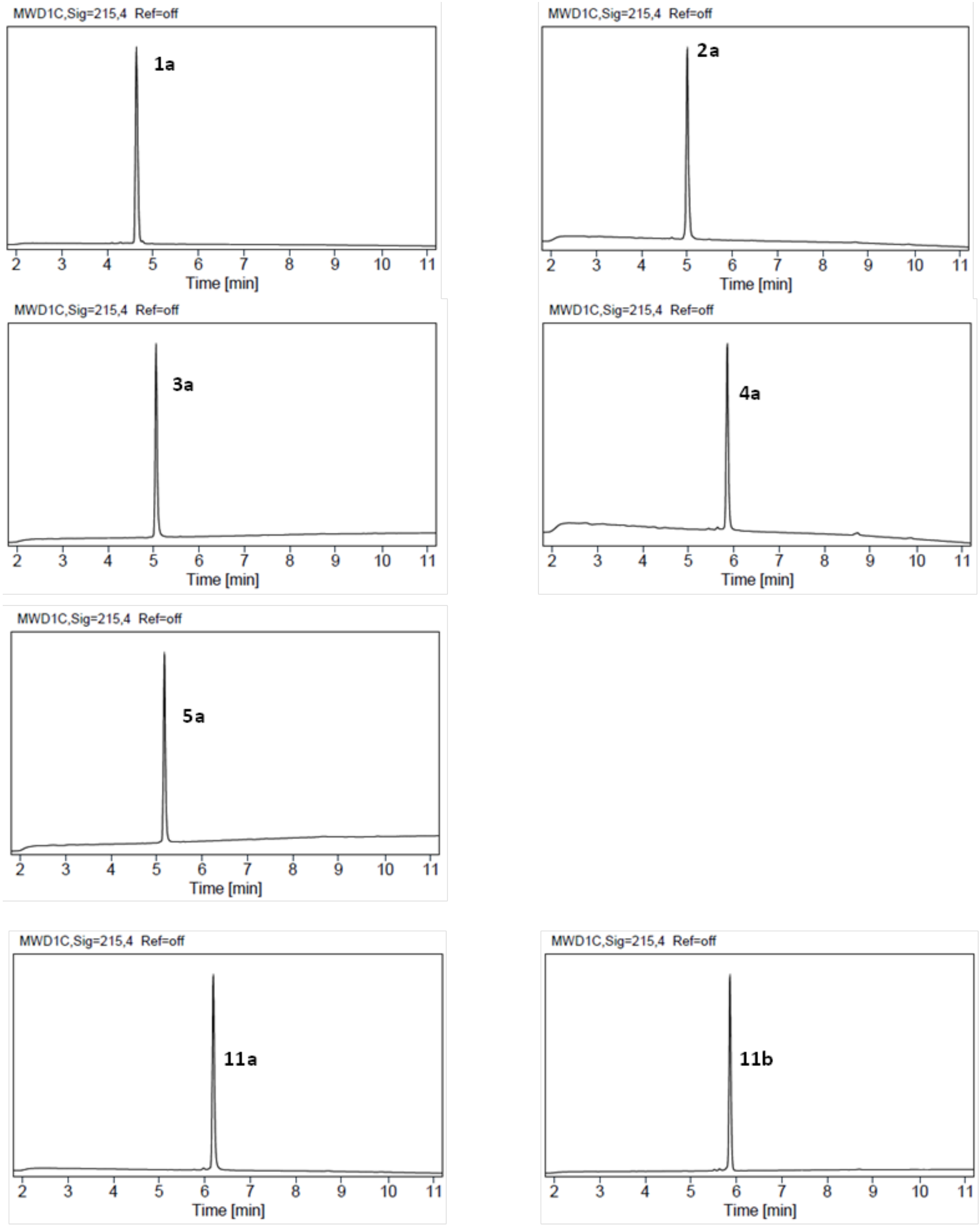

HPLC/UPLC peptide purity traces recorded at 210 or 215 nm.

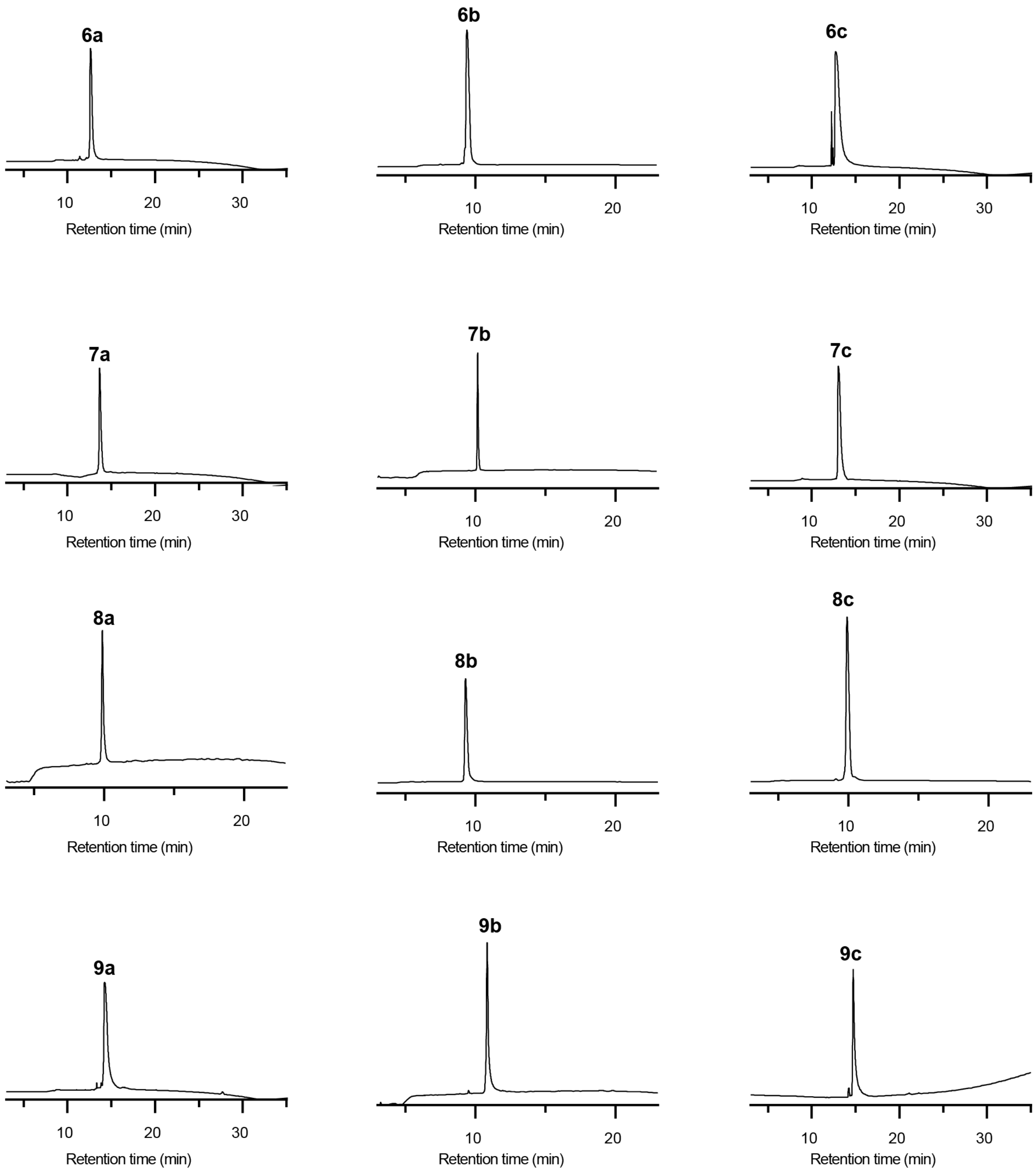

HPLC/UPLC peptide purity traces recorded at 210 or 215 nm.

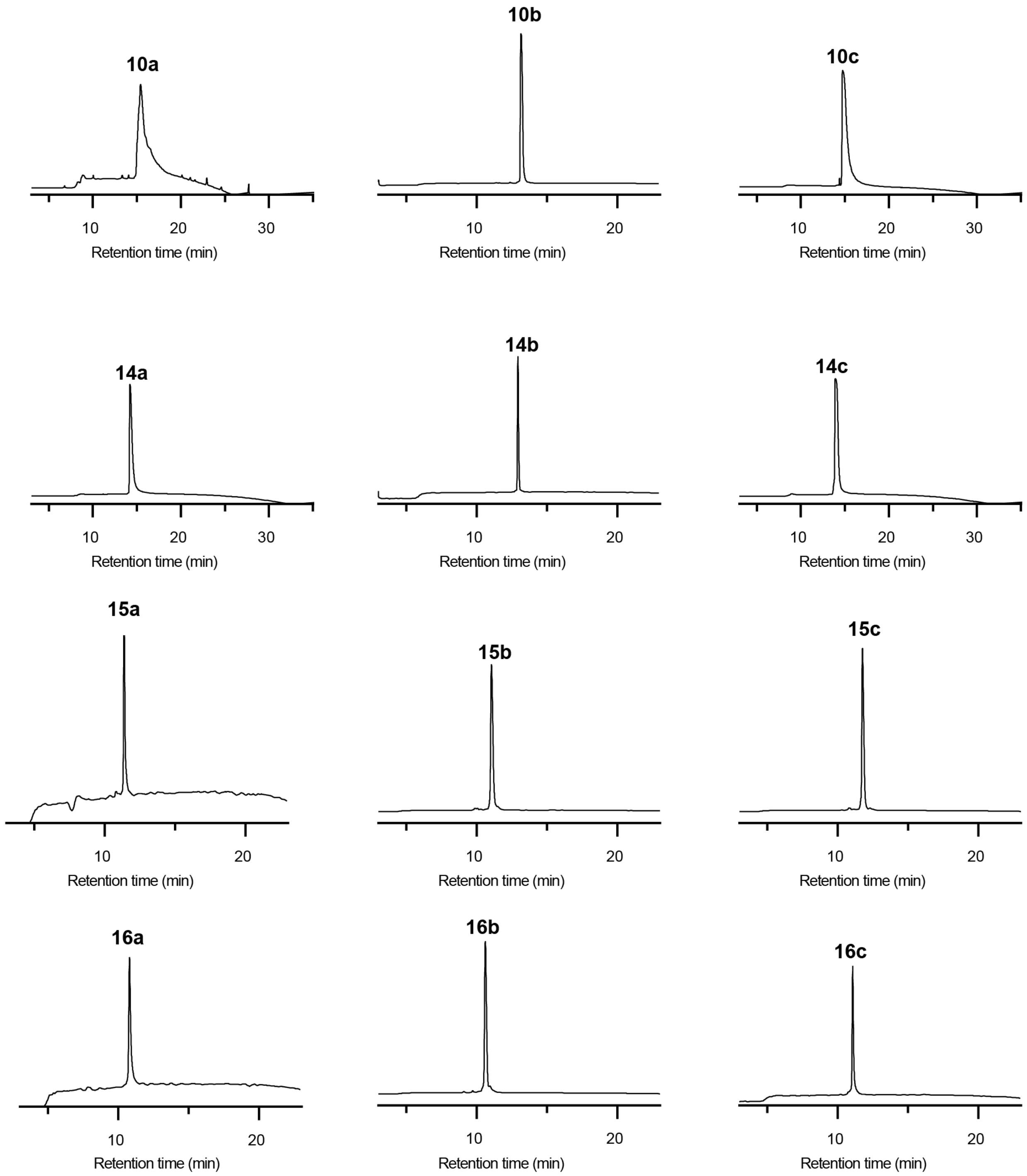

HPLC/UPLC peptide purity traces recorded at 210 or 215 nm.

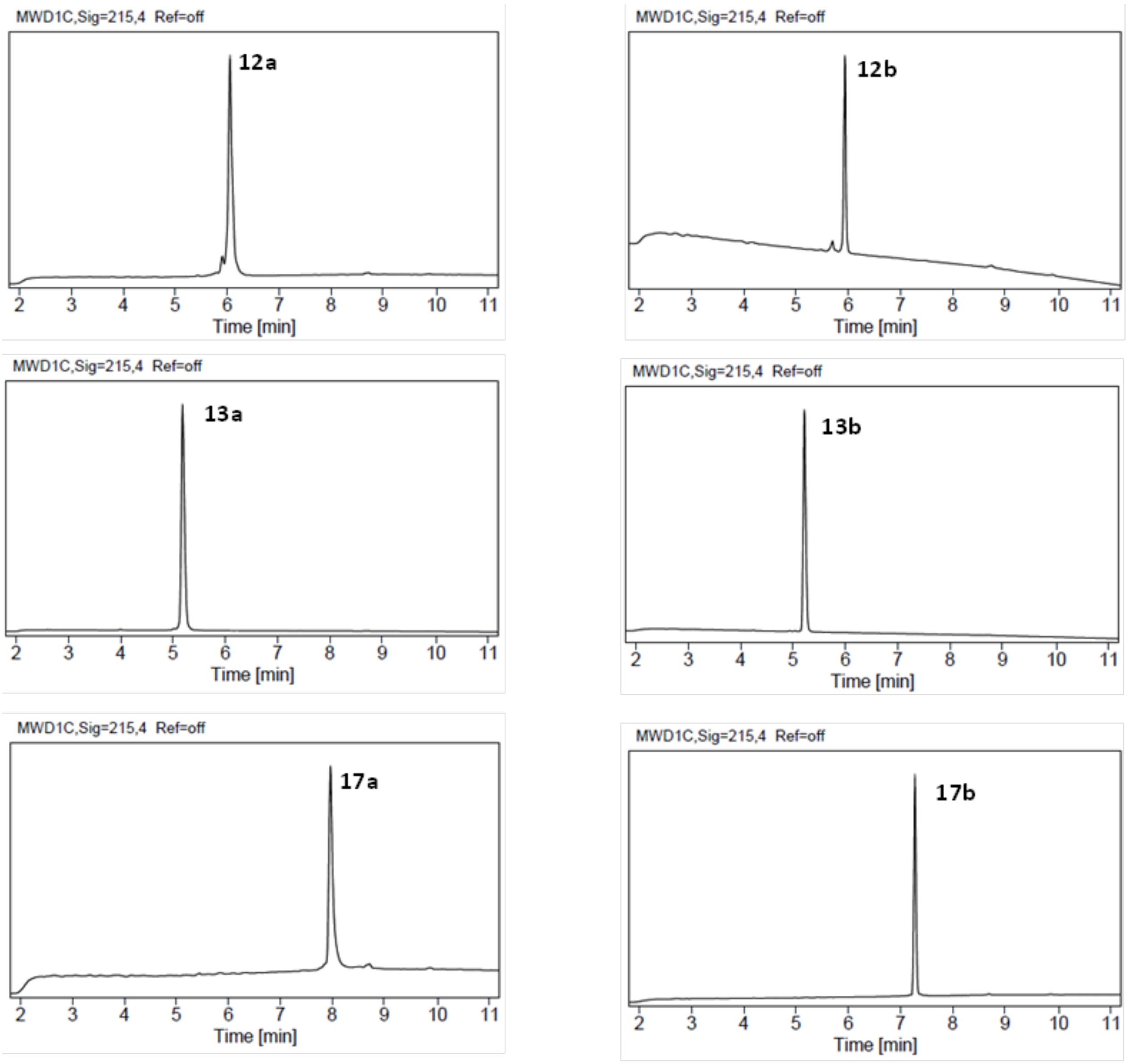

HPLC/UPLC peptide purity traces recorded at 210 or 215 nm.

Supplementary references

1 Thomsen, M. C. & Nielsen, M. Seq2Logo: a method for construction and visualization of amino acid binding motifs and sequence profiles including sequence weighting, pseudo counts and two-sided representation of amino acid enrichment and depletion. *Nucleic Acids Res.* **40**, 281-287 (2012).

2 Kahnberg, P. *et al.* Design, synthesis, potency, and cytoselectivity of anticancer agents derived by parallel synthesis from α-aminosuberic acid. *J. Med. Chem.* **49**, 7611-7622 (2006).

3 Lederer, M. O. Reactivity of lysine moieties toward γ-Hydroxy-α, β-unsaturated Epoxides: A model study on protein− lipid oxidation product interaction. *J. Agric. Food. Chem.* **44**, 2531-2537 (1996).

4 Moore, S. A., Ferhatoglu, Y., Jia, Y., Al-Jiab, R. A. & Scott, M. J. Structural and biochemical studies on the chromo-barrel domain of male specific lethal 3 (MSL3) reveal a binding preference for mono-or dimethyllysine 20 on histone H4. *J. Biol. Chem.* **285**, 40879-40890 (2010).

5 Budavari, S., O'Neil, M. & Smith, A. The Merck Index. Whitehouse Station, NJ: Merck & Co. *Inc* **1070**, 163 (1996).
