# Supplementary material for: Hydroxamic acid-modified peptide microarrays for profiling isozyme-selective interactions and inhibition of histone deacetylases
Source: Nat Commun. 2021 Jan 4;12:62. doi: 10.1038/s41467-020-20250-9 (PMC7782793; doi:10.1038/s41467-020-20250-9)
Supplement: Supplementary file 3 — Reporting Summary [file 41467_2020_20250_MOESM3_ESM.pdf]

nature research

Corresponding author(s): Hans M. Maric and Christian Adam Olsen

Last updated by author(s): Nov 18, 2020

Reporting Summary

Nature Research wishes to improve the reproducibility of the work that we publish. This form provides structure for consistency and transparency in reporting. For further information on Nature Research policies, see our Editorial Policies and the Editorial Policy Checklist.

Statistics

For all statistical analyses, confirm that the following items are present in the figure legend, table legend, main text, or Methods section.

n/a	Confirmed	
☐	☒	The exact sample size (*n*) for each experimental group/condition, given as a discrete number and unit of measurement
☐	☒	A statement on whether measurements were taken from distinct samples or whether the same sample was measured repeatedly
☒	☐	The statistical test(s) used AND whether they are one- or two-sided<br>*Only common tests should be described solely by name; describe more complex techniques in the Methods section.*
☒	☐	A description of all covariates tested
☒	☐	A description of any assumptions or corrections, such as tests of normality and adjustment for multiple comparisons
☐	☒	A full description of the statistical parameters including central tendency (e.g. means) or other basic estimates (e.g. regression coefficient) AND variation (e.g. standard deviation) or associated estimates of uncertainty (e.g. confidence intervals)
☒	☐	For null hypothesis testing, the test statistic (e.g. *F*, *t*, *r*) with confidence intervals, effect sizes, degrees of freedom and *P* value noted<br>*Give P values as exact values whenever suitable.*
☒	☐	For Bayesian analysis, information on the choice of priors and Markov chain Monte Carlo settings
☒	☐	For hierarchical and complex designs, identification of the appropriate level for tests and full reporting of outcomes
☒	☐	Estimates of effect sizes (e.g. Cohen's *d*, Pearson's *r*), indicating how they were calculated

Our web collection on statistics for biologists contains articles on many of the points above.

Software and code

Policy information about availability of computer code

Data collection	Microarray data was collected using Array Analyze software (Active Motif, v1), except for the assay linearity test, which was measured using FIJI (open source) with the Microarray Profile addon (OptiNav, v1).
Data analysis	Data analysis and representation was performed with GraphPad Prism software, versions 7 and 8.

For manuscripts utilizing custom algorithms or software that are central to the research but not yet described in published literature, software must be made available to editors and reviewers. We strongly encourage code deposition in a community repository (e.g. GitHub). See the Nature Research guidelines for submitting code & software for further information.

Data

Policy information about availability of data

All manuscripts must include a data availability statement. This statement should provide the following information, where applicable:

- Accession codes, unique identifiers, or web links for publicly available datasets
- A list of figures that have associated raw data
- A description of any restrictions on data availability

The authors declare that the data supporting the findings of this study are available within the paper and its supplementary information files. Source data for Figs. 2–7 and Supplementary Figs. 3–7, 9, 10 and 12 are provided with the paper.

Field-specific reporting

Please select the one below that is the best fit for your research. If you are not sure, read the appropriate sections before making your selection.

- [x] Life sciences
- [ ] Behavioural & social sciences
- [ ] Ecological, evolutionary & environmental sciences

For a reference copy of the document with all sections, see nature.com/documents/nr-reporting-summary-flat.pdf

Life sciences study design

All studies must disclose on these points even when the disclosure is negative.

Sample size	No sample-size calculation was performed. Sample sizes of n = 4 for microarray assays were based on previous studies (https://doi.org/10.1021/acs.jmedchem.9b01087). Sample sizes of n = 2 for HDAC substrate and inhibitor assays were based on our own reproducibility experience (doi: 10.1021/acs.biochem.7b00725; https://doi.org/10.1016/j.chembiol.2018.04.007) and that of others (doi: 10.1038/nchembio.313). Sample size of n = 4 for cellular assays was based on our own reproducibility experience (doi: 10.1021/acschemneuro.9b00281) and that of others (doi: 10.1021/acs.jmedchem.8b01936).
Data exclusions	Non-sigmoidal microarray dose-response curves were curated manually, classified as N.D. (not determined) and are included in the Source Data file for transparency. Additional values were excluded when top values were outside of the 0.8-1.2 range and/or pEC50 values were below the studied enzyme concentration range, as indicative of incomplete dose-response data within the studied range. The corresponding peptides were considered weak binders. These criteria were established during preliminary experiments.
Replication	All microarray experiments were reproduced on at least two slides. All inhibitor and substrate experiments were reproduced at least twice and with different batches of enzyme, and data on multiple inhibitors and substrates were replicated once by a different author. Western blots were reproduced on four independent biological replicates.
Randomization	The experiments were not randomized, since no clinical trials or population studies were conducted.
Blinding	The experiments were not blinded, since no clinical trials or populations studies were conducted.

Reporting for specific materials, systems and methods

We require information from authors about some types of materials, experimental systems and methods used in many studies. Here, indicate whether each material, system or method listed is relevant to your study. If you are not sure if a list item applies to your research, read the appropriate section before selecting a response.

Materials & experimental systems

n/a	Involved in the study	
[ ]	[x]	Antibodies
[ ]	[x]	Eukaryotic cell lines
[x]	[ ]	Palaeontology and archaeology
[x]	[ ]	Animals and other organisms
[x]	[ ]	Human research participants
[x]	[ ]	Clinical data
[x]	[ ]	Dual use research of concern

Methods

n/a	Involved in the study	
[x]	[ ]	ChIP-seq
[x]	[ ]	Flow cytometry
[x]	[ ]	MRI-based neuroimaging

Antibodies

Antibodies used	anti-6x-His tag HRP-linked antibody (His.H8): ThermoFisher, MA1-21315-HRP; anti-6x-His tag antibody: ThermoFisher, MA1-21315, lot: UF283577; anti-acetylated a-tubulin: Santa Cruz Biotechnology, 6-11B-1, sc-23950; anti-vinculin: Cell Signaling Technology, E1E9V XP, 13901, lot: 6; anti-acetyl-histone H3 (K36): Cell Signaling Technology, D9T5Q, 27683, lot: 1; anti-acetyl-histone H3 (K27): Cell Signaling Technology, 4353, lot: 1; anti-rabbit HRP-linked antibody: Cell Signaling Technology, 7074S; anti-mouse HRP-linked antibody: Cell Signaling Technology, 7076S; anti-mouse HRP-linked antibody: ThermoFisher, 31430, lot: UJ293428.
Validation	All antibodies were validated by the manufacturer and accompanied by peer-reviewed publications showing their use.<br>ThermoFisher: His tag antibodies were validated by Western blot on purified His-tagged proteins and bacterial and human cell lysates from transfected cells. ThermoFisher: secondary anti-mouse antibody was validated by Western blot, immunohistochemistry, immunoprecipitation and ELISA using multiple ThermoFisher primary antibodies. Santa Cruz Biotechnology: Ac-tubulin antibody was validated by Western blot on lysates from multiple cell lines, immunofluorescence staining on human cells, and immunoperoxidase staining on human tissue. Cell Signaling Technology: vinculin antibody was validated by Western blot on lysates from multiple human cell lines and immunohistochemical analysis of multiple human tissues. Cell Signaling Technology: acetyl-histone antibodies were validated by Western blot on lysates from human cell lines, and chromatin immunoprecipitation analysis of human cells (from the respecive websites: "This antibody has been validated using SimpleChIP® Enzymatic Chromatin IP Kits"). Cell Signaling Technology: secondary anti-rabbit and anti-mouse antibodies had the following statement on the vendor's website: "This product is thoroughly

validated with CST primary antibodies and will work optimally with the CST western immunoblotting protocol, ensuring accurate and reproducible results."

Eukaryotic cell lines

Policy information about cell lines

Cell line source(s)	HEK293T cells (ATCC) were provided by the Pless lab at the Department of Drug Design and Pharmacology, University of Copenhagen.
Authentication	None of the cell lines used were authenticated in our lab.
Mycoplasma contamination	All cell lines were tested negative for mycoplasma contamination.
Commonly misidentified lines (See ICLAC register)	No commonly misindentified cell lines were used in the study.
